# Supplementary material for: An LNA-amide modification that enhances the cell uptake and activity of phosphorothioate exon-skipping oligonucleotides
Source: Nat Commun. 2022 Jul 12;13:4036. doi: 10.1038/s41467-022-31636-2 (PMC9276774; doi:10.1038/s41467-022-31636-2)
Supplement: Supplementary file 1 — Supplementary Information [file 41467_2022_31636_MOESM1_ESM.pdf]

Supplementary Information

An LNA-amide modification that enhances the cell uptake and activity of phosphorothioate exon-skipping oligonucleotides

Ysobel R. Baker[1], Cameron Thorpe[1], Jinfeng Chen[1], Laura M. Poller[1], Lina Cox[1], Pawan Kumar[1], Wooi F. Lim[2], Lillian Lie[1], Graham McClorey[2], Sven Epple[1], Daniel Singleton[3], Michael A. McDonough[1], Jack S. Hardwick[1], Kirsten E. Christensen[1], Matthew J. A. Wood[2], James P. Hall[4], Afaf H. El-Sagheer[1,5], Tom Brown[1*].

1. Department of Chemistry, University of Oxford, Chemistry Research Laboratory, 12 Mansfield Road, Oxford, OX1 3TA, UK.

2. Department of Paediatrics, University of Oxford, LGC building, South Parks Road, Oxford, OX1 3QX, UK.

3. ATDBio Ltd, School of Chemistry, University of Southampton, Highfield, Southampton SO17 1BJ, UK.

4. Department of Pharmacy, Chemistry and Pharmacy Building, University of Reading, Whiteknights, Reading, RG6 6AD, UK.

5. Department of Science and Mathematics, Suez University, Faculty of Petroleum and Mining Engineering, Suez 43721, Egypt.

* Corresponding author, e-mail: tom.brown@chem.ox.ac.uk

Table of Contents

Supplementary notes **4**

Supplementary tables **4**

Supplementary figures **8**

Small molecule synthesis supplementary figures 8
UPLC and MS analysis of oligonucleotides with LNA-amide linkages 10
Representative UV melting curves 15
Nuclease resistance supplementary figure 20
XRD supplementary figures 20
Supplementary cell assay figures 25
UPLC-MS traces of LNA-amide modified oligonucleotides 27

Supplementary small molecule crystallography information **38**

Supplementary Methods **41**

Small molecule synthesis **41**

General 41
4-*C*-(Methanesulfonyloxymethyl)-5-*O*-methanesulfonyl-1,2-*O*-isopropylidene-α-D-ribofuranose **2** 42
4-*C*-(Methanesulfonyloxymethyl)-5-*O*-methanesulfonyl-1,2-*O*-isopropylidene-α-D-erythro-pentofuranos-3-ulose **3** 42
Ethyl 2-((3aR,6aR)-2,2-dimethyl-5,5-bis(((methylsulfonyl)oxy)methyl)dihydrofuro[2,3-d][1,3]dioxol-6(5H)-ylidene)acetate **4** 43
Ethyl 2-((3aR,6S,6aR)-2,2-dimethyl-5,5-bis(((methylsulfonyl)oxy)methyl)tetrahydrofuro[2,3-d][1,3]dioxol-6-yl)acetate **5** 44
(3R,4S)-4-(2-Ethoxy-2-oxoethyl)-5,5-bis(((methylsulfonyl)oxy)methyl)tetrahydrofuran-2,3-diyl diacetate **6** 45
Thymine LNA acid precursor **7a** 45
N^4-Benzoylcytosine LNA acid precursor **7b** 46
N^4-Benzoyl 5-methylcytosine LNA precursor **7c** 47
N^6-Benzoyladenine LNA acid precursor **7d** 47
N^2-Isobutyrylguanine LNA acid precursor **7e** 48
Thymine LNA acid **8a** 49
N^4-Benzoylcytosine LNA acid **8b** 49
N^4-Benzoyl 5-methylcytosine LNA acid **8c** 50
N^6-Benzoyladenine LNA acid **8d** 51
N^2-Isobutyrylguanine LNA acid **8e** 51
5′-*O*-DMT thymine LNA acid **9a** 52
5′-*O*-DMT N^4-benzoylcytosine LNA acid **9b** 53
5′-*O*-DMT N^4-benzoyl methylcytosine LNA acid **9c** 53
5′-*O*-DMT N^6-benzoyladenine LNA acid **9d** 54
5′-*O*-DMT N^2-isobutyrylguanine LNA acid **9e** 55
N_3 Thymine LNA **S14**[4] 56
Amino thymine LNA **S11**[4] 56
5′-*N*-MMT thymine LNA **S12**[3] 56
5′-*N*-MMT thymine LNA phosphoramidite **10**[3] 57

Oligonucleotide synthesis **58**

DNA synthesis and cleavage 58
RNA synthesis and cleavage 58
2′OMe phosphodiester oligonucleotide synthesis and cleavage 58
Phosphodiester oligonucleotide purification 58
Phosphorothioate oligonucleotide synthesis, cleavage and purification 59
Oligonucleotide analysis 59

LNA-amide modified oligonucleotide synthesis **61**

Oligonucleotide segment synthesis 61
Amino monomer addition 61
Amide bond formation on resin (peptide coupling) 61

Cleavage of oligonucleotides from resin, deprotection and purification ... 61

Biophysical studies ... 61

UV melting experiments ... 61

Oligonucleotide X-Ray crystallography ... 62

Crystallisation ... 62

Data collection and processing ... 62

Structure solution, model building and refinement ... 62

Biological assays ... 63

Evaluation of stability in fetal bovine serum (FBS) ... 63

Cell culture ... 63

Transfection with Lipofectamine 2000 ... 63

Gymnosis experiments ... 63

Luciferase assay ... 63

WST-1 cell viability assay ... 63

NMR spectra of compounds 2-10 ... 65

Supplementary References ... 87

Supplementary notes

Supplementary tables

Supplementary Table 1. List of oligonucleotides (ONs) used in this study with calculated and found mass spectrometry (MS) data. UPLC-MS traces for all ONs are given (Supplementary Fig. 3-7 and Fig. 26-36).

ON	Sequence (5´ ® 3´)	Backbone	Expected	Found
Single amide addition UV melting studies				
ON1$^{\text{DNA-Am-DNA}}$	CGACGCT*TGCAGC	DNA	3896.7	3897.0
ON2$^{\text{LNA-Am-DNA}}$	CGACGCT*TGCAGC	DNA	3924.8	3925.0
ON3$^{\text{DNA-Am-LNA}}$	CGACGCT*TGCAGC	DNA	3924.8	3925.0
ON4$^{\text{LNA-Am-LNA}}$	CGACGCT*TGCAGC	DNA	3952.9	3953.0
ON5$^{\text{LNA-LNA}}$	CGACGCTTGCAGC	DNA	3995.5	3992.0
ON6$^{\text{DNA-DNA}}$	CGACGCTTGCAGC	DNA	3935.5	3936.0
Single amide addition UV melting targets				
ON7	GCTGCAAGCGTCG	DNA	3975.5	3976.0
ON8	GCUGCAAGCGUCG	RNA	4155.2	4156.5
ON9	GCUGCACGCGUCG	RNA	4131.2	4132.0
ON10	GCUGCCAGCGUCG	RNA	4131.2	4132.0
ON11	GCUGCAGGCGUCG	RNA	4171.2	4171.5
ON12	GCUGCGAGCGUCG	RNA	4171.2	4172.4
Multiple amide addition in different backbones				
ON13 $^{\text{DNA/1LAL/16PO}}$	CCTCTTACC*TCAGTTACA	DNA, PO	5386.8	5387.5
ON14 $^{\text{DNA/4LAL/13PO}}$	CC*TCT*TACC*TCAGT*TACA	DNA, PO	5438.7	5439.0
ON15$^{\text{DNA/17PO}}$	CCTCTTACCTCAGTTACA	DNA, PO	5369.6	5370.0
ON16$^{\text{2´OMe/4LAL/13PO}}$	C^{Me}C*TCT*TAC^{Me}C*TCAGT*TACA	2'OMe, PO	5766.9	5767.0
ON17$^{\text{2´OMe/17PO}}$	CCUCUUACCUCAGUUACA	2´OMe, PO	5824.7	5825.0
ON18$^{\text{2´OMe/4LAL/13PS}}$	C^{Me}C*TCT*TAC^{Me}C*TCAGT*TACA	2´OMe, PS	5976.2	5975.5
ON19$^{\text{2´OMe/8LNA/17PS}}$	C^{Me}CTCTTAC^{Me}CTCAGTTACA	2'OMe, PS	6193	6195.7
ON20$^{\text{2´OMe/17PS}}$	CCUCUUACCUCAGUUACA	2´OMe, PS	6096.3	6097.6
Multiple amide addition in different backbones UV melting targets				
ON21	TGTAACTGAGGTAAGAGG	DNA	5627.6	5628.5
ON22	UGUAACUGAGGUAAGAGG	RNA	5858.9	5859.0
ON23	AGGTAAGAGG	DNA	3141.0	3141.5
ON24	AGGUAAGAGG	RNA	3286.8	3288.0
Additional digestion oligonucleotide				
ON25$^{\text{DNA/8LNA/17PO}}$	C^{Me}CTCTTAC^{Me}CTCAGTTACA	DNA	5621.6	5622.8
XRD oligonucleotides				
ON26^{xDNA}	CTTTTCTTTG	DNA	2974.9	2976.0
ON27^{xRNA}	CAAAGAAAAG	RNA	3238.0	3240.0
ON28$^{\text{xDNA-Am-DNA}}$	CTT*TTCTTTG	DNA	2936.1	2937.0
ON29$^{\text{xLNA-Am-DNA}}$	CTT*TTCTTTG	DNA	2964.2	2964.5
ON30$^{\text{xLNA-Am-LNA}}$	CTT*TTCTTTG	DNA	2992.3	2992.5
Scrambled control splice-switching oligonucleotide				
ON31$^{\text{2´OMe/17PS scrambled}}$	CCUCAUUCACUCGAUUCA	2´OMe, PS	6096.3	6099.7
Oligonucleotides in the supplementary information				
ONS1	CGACGCC*TGCAGC	DNA	3937.8	3938.5
ONS2	CGACGC^{Me}C*TGCAGC	DNA	3951.9	3953.0
ONS3	CGACGCA*TGCAGC	DNA	3961.9	3963.0
ONS4	CGACGCG*TGCAGC	DNA	3977.9	3978.5

Underlined red bases indicates a locked sugar; * is an amide bond in place of a phosphodiester, underlined blue bases indicates the position of the mismatch. Backbone denotes to the chemistry of inter-sugar linkages and the sugars not flanking an amide bond.

Supplementary Table 2. Comparison of the melting temperatures of duplexes containing a single amide substitution of the phosphodiester backbone flanked by LNA on the 5´, 3´ or both sides within a DNA backbone hybridised to DNA or RNA.

ON	Sequence	DNA match T_m (ΔT_m) ON7	RNA match T_m (ΔT_m) ON8	RNA 5´ C mismatch T_m (ΔT_m) ON9	RNA 3´ C mismatch T_m (ΔT_m) ON10	RNA 5´ G mismatch T_m (ΔT_m) ON11	RNA 3´ G mismatch T_m (ΔT_m) ON12
**ON1**$^{DNA\text{-}Am\text{-}DNA}$	CGACGCT*TGCAGC	61.2 (-2.3)	61.0 (-0.4)	47.0 (-14.1)	50.2 (-10.8)	57.3 (-3.7)	56.6 (-3.4)
**ON2**$^{LNA\text{-}Am\text{-}DNA}$	CGACGC**T***TGCAGC	63.1 (-0.3)	64.4 (+3.0)	50.2 (-14.2)	51.0 (-13.4)	58.5 (-5.9)	60.3 (-4.1)
**ON3**$^{DNA\text{-}Am\text{-}LNA}$	CGACGCT***T**GCAGC	60.9 (-2.5)	63.9 (+2.5)	50.1 (-13.8)	52.5 (-11.4)	58.8 (-5.1)	56.9 (-7.0)
**ON4**$^{LNA\text{-}Am\text{-}LNA}$	CGACGC**T***__T__GCAGC	63.4 (-0.1)	66.5 (+5.1)	53.3 (-13.2)	55.1 (-11.4)	59.8 (-6.7)	60.5 (-6.0)
**ON5**$^{LNA\text{-}LNA}$	CGACGC**TT**GCAGC	69.6 (+6.1)	74.1 (+12.7)	59.7 (-14.4)	59.9 (-14.2)	68.6 (-5.5)	67.3 (-2.3)
**ON6**^{DNAcontrol}	CGACGCTTGCAGC	63.5	61.4	46.2 (-15.2)	51.2 (-10.2)	57.4 (-4.0)	57.4 (-4.0)

T_m values were measured using 3.0 µM concentrations of each oligonucleotide strand in 10 mM phosphate buffer (pH 7.0) containing 200 mM NaCl. **T** indicates a locked sugar and * is an amide bond in place of a phosphodiester. T_m values were calculated as the maximum of the first-derivative of the melting curve (A_{260} vs T) and reported as the average of at least two independent experiments. ΔT_m for matched sequences = modified – ON6^{DNAcontrol}; ΔT_m for mismatched = Match - RNA mismatch. Target ON sequences, where X denotes the mismatch. ON7 = GCTGCAAGCGTCG; ON8 = GCUGCAAGCGUCG; ON9 = GCUGCACGCGUCG; ON10 = GCUGCCAGCGUCG; RNA ON11 = GCUGCAGGCGUCG. ON12 = GCUGCGAGCGUCG. Representative melting curves are given (Supplementary Fig. S8-13).

Supplementary Table 3. Comparison of the relative melting temperatures of duplexes containing 0, 1 or 4 amide linkages flanked by LNA on both sides hybridised to DNA or RNA.

ON	Sequence (5´ → 3´)	*backbone*	DNA T_m (ΔT_m)	RNA T_m (ΔT_m)
**a**				
ON13 $^{DNA/1LAL/16PO}$	CCTCTTACC*TCAGTTACA	DNA, PO	60.9 (+1.8)	65.8 (+2.2)
ON14 $^{DNA/4LAL/13PO}$	CC*TCT*TACC*TCAGT*TACA	DNA, PO	64.2 (+5.1)	76.6 (+13.0)
ON15$^{DNA/17PO}$	CCTCTTACCTCAGTTACA	DNA, PO	59.1	63.6
**b**				
ON14 $^{DNA/4LAL/13PO}$	CC*TCT*TACC*T_cagt*taca_	DNA, PO	46.5 (+7.0)	64.3 (+17.5)
ON15$^{DNA/17PO}$	CCTCTTACCT_cagttaca_	DNA, PO	39.5	46.8
**c**				
ON16$^{2'OMe/4LAL/13PO}$	C^{Me}C*TCT*TAC^{Me}C*T_cagt*taca_	2'OMe, PO	53.2 (+19.1)	72.3 (+9.3)
ON17$^{2'OMe/17PO}$	CCUCUUACCU_caguuaca_	2´OMe, PO	34.1	63.0
**d**				
ON18$^{2'OMe/4LAL/13PS}$	C^{Me}C*TCT*TAC^{Me}C*T_cagt*taca_	2´OMe, PS	48.1 (+25.4)	67.4 (+10.3)
ON19$^{2'OMe/8LNA/17PS}$	C^{Me}CTCTTAC^{Me}CT_cagttaca_	2'OMe, PS	59.0 (+36.3)	>80
ON20$^{2'OMe/17PS}$	CCUCUUACCU_caguuaca_	2´OMe, PS	22.7	57.1

Experimental conditions as in Supplementary Table 2. **a** = comparison of DNA ONs against full length targets, **b** = comparison of DNA ONs against 10-mer targets, **c** = comparison of 2´OMe/PO ONs, **d** = comparison of 2´OMe/PS ONs. Backbone: PO = phosphodiester, PS = phosphorothioate, DNA = deoxyribose sugars, 2´OMe = 2´OMe RNA sugars. **X** indicate a locked sugar and * indicates an amide bond in place of a phosphodiester, LAL indicates the number of LNA-flanked amide bonds. DNA target (ON21) = TGTAACTGAGGTAAGAGG; RNA target (ON22) = UGUAACUGAGGUAAGAGG. Truncated DNA target (ON23) = AGGTAAGAGG. Truncated RNA target (ON24) = AGGUAAGAGG. ΔT_m = modified – control. Bases in lower case italic remain single stranded on duplex formation and do not contribute to T_m. Representative melting curves are given (Supplementary Fig. 14-17).

Supplementary Table 4. Sequences of oligonucleotides used in crystallographic studies.

ON	Sequence (5´ → 3´)	*backbone*
ON26^{xDNA}	CTTTTCTTTG	DNA
ON27^{xRNA}	CAAAGAAAAG	RNA
ON28$^{xDNA-Am-DNA}$	CTT*TTCTTTG	DNA
ON29$^{xLNA-Am-DNA}$	CTT*TTCTTTG	DNA
ON30$^{xLNA-Am-LNA}$	CTT*TTCTTTG	DNA

T indicates a locked sugar and * indicates an amide bond in place of a phosphodiester.

Supplementary Table 5. Summary of data processing for XRD structures of DNA:RNA hybrids containing amide and LNA modifications. Data was validated using pdb validation. Each dataset was collected from a single crystal, values shown in parenthesis are for the highest resolution shell.

Data collection	ON26^{xDNA}:ON27^{xRNA}	ON28$^{xDNA\text{-}Am\text{-}DNA}$: ON27^{xRNA}	ON29$^{xLNA\text{-}Am\text{-}DNA}$: ON27^{xRNA}	ON30$^{xLNA\text{-}Am\text{-}LNA}$: ON27^{xRNA}
PDB ID	*7NRP*	*7OOS*	*7OZZ*	*7OOO*
Space group	$P\ 6_1$	$P\ 6_1$	$P\ 6_1$	$P\ 3_221$
Cell dimensions				
a, b, c / Å	53.34, 53.34, 45.88	55.14, 55.14, 45.96	54.12, 54.12, 48.84	49.15, 49.15, 90.96
α, β, γ / °	90.00 90.00 120.00	90.00 90.00 120.00	90.00 90.00 120.00	90.00 90.00 120.00
Detector distance / cm	19.8	48.5	48.5	17.0
Exposure time per image / s	0.090	0.038	0.056	0.040
Resolution (Å)	27.17-2.67 (2.80-2.67)	47.75-2.60 (2.70-2.60)	27.06-2.70 (2.77-2.70)	31.08-2.56 (2.63-2.56)
No. unique reflections	2243	2517	2283	4398
R_{meas}	0.081 (1.776)	0.121 (1.124)	0.055 (1.816)	0.078 (3.673)
$I/\sigma(I)$	25.2 (1.7)	8.9 (2.2)	23.0 (1.1)	19.8 (0.8)
$CC_{1/2}$	0.996 (0.646)	0.943 (0.645)	0.999 (0.421)	0.999 (0.329)
Completeness /%	98.8 (97.0)	99.9 (100.0)	99.9 (92.6)	99.4 (94.2)
Multiplicity	19.3 (16.0)	7.5 (7.1)	9.7 (9.0)	18.7 (18.5)
Refinement				
Resolution / Å	27.17-2.67 (2.76-2.67)	47.75-2.60 (2.69-2.60)	27.06-2.70 (2.79-2.70)	31.08-2.56 (2.65-2.56)
No. reflections	2226	2502	2267	4349
R_{work} / R_{free}	0.170, 0.191	0.218, 0.234	0.200, 0.237	0.237, 0.261
No. atoms / ASU	422	419	416	837
DNA	197	196	198	434
RNA	217	217	217	400
Mg^{2+}	-	-	-	2
As	1	-	-	-
Sr^{2+}	-	2	-	-
K^{+}	-	-	1	-
Ligand atoms	7	4	-	1
Wilson *B* factor (Å^2)	75.0	68.0	102.1	95.1
r.m.s. deviations				
Bond lengths / Å	0.009	0.005	0.007	0.008
Bond angles / °	1.20	0.53	0.76	0.94
r.m.s.d Unmod / Å	-	0.45	0.42	2.51

Supplementary Table 6. DNA:RNA duplex crystallisation conditions.

Structure	Conditions	
	Drop solution	Well solution
Unmodified<br>ON26^{xDNA}:ON27^{xRNA}	0.5 mM duplex<br>12 mM $Mg(OAc)_2$<br>0.6 mM spermidine.HCl<br>0.075% β-octylglucoside<br>12 mM sodium cacodylate pH 5.8<br>12% MPD	MPD:H_2O, 1:1
Amide<br>ON28$^{xDNA\text{-}Am\text{-}DNA}$:ON27^{xRNA}	0.5 mM duplex<br>20 mM LiCl<br>40 mM $SrCl_2{\cdot}6H_2O$<br>20 mM sodium cacodylate trihydrate pH 7.0<br>15% (v/v) MPD<br>6.0 mM spermine tetrahydrochloride	double the concentration of the drop solution without the oligonucleotide duplex
LNA-amide<br>ON29$^{xLNA\text{-}Am\text{-}DNA}$:ON27^{xRNA}	0.5 mM duplex<br>40 mM NaCl<br>6.0 mM KCl<br>20 mM sodium cacodylate trihydrate pH 7.0<br>17.5% MPD<br>6.0 mM spermine tetrahydrochloride	double the concentration of the drop solution without the oligonucleotide duplex
LNA-Am-LNA<br>ON30$^{xLNA\text{-}Am\text{-}LNA}$:ON27^{xRNA}	0.5 mM duplex<br>40 mM NaCl<br>6.0 mM KCl<br>20 mM sodium cacodylate trihydrate pH 6.0<br>15% (v/v) MPD<br>6.0 mM spermine tetrahydrochloride	double the concentration of the drop solution without the oligonucleotide duplex

Supplementary figures

Small molecule synthesis supplementary figures

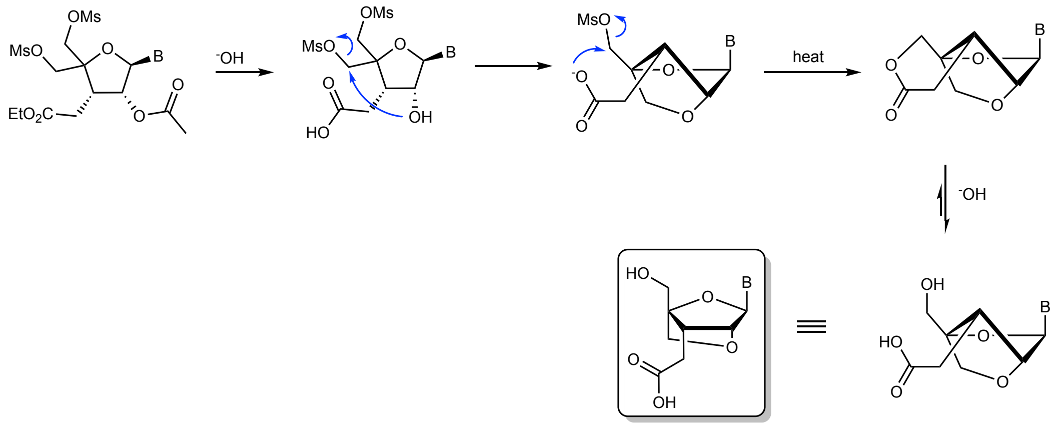

Supplementary Figure 1. Proposed neighbouring group participation accounting for the facile displacement of the 5´-mesyl by a hydroxide. Here the carboxylate displaces the 5´-mesyl forming a lactone which is subsequently opened by hydrolysis.

Obika route

36% yield in 12 steps from commercially available material

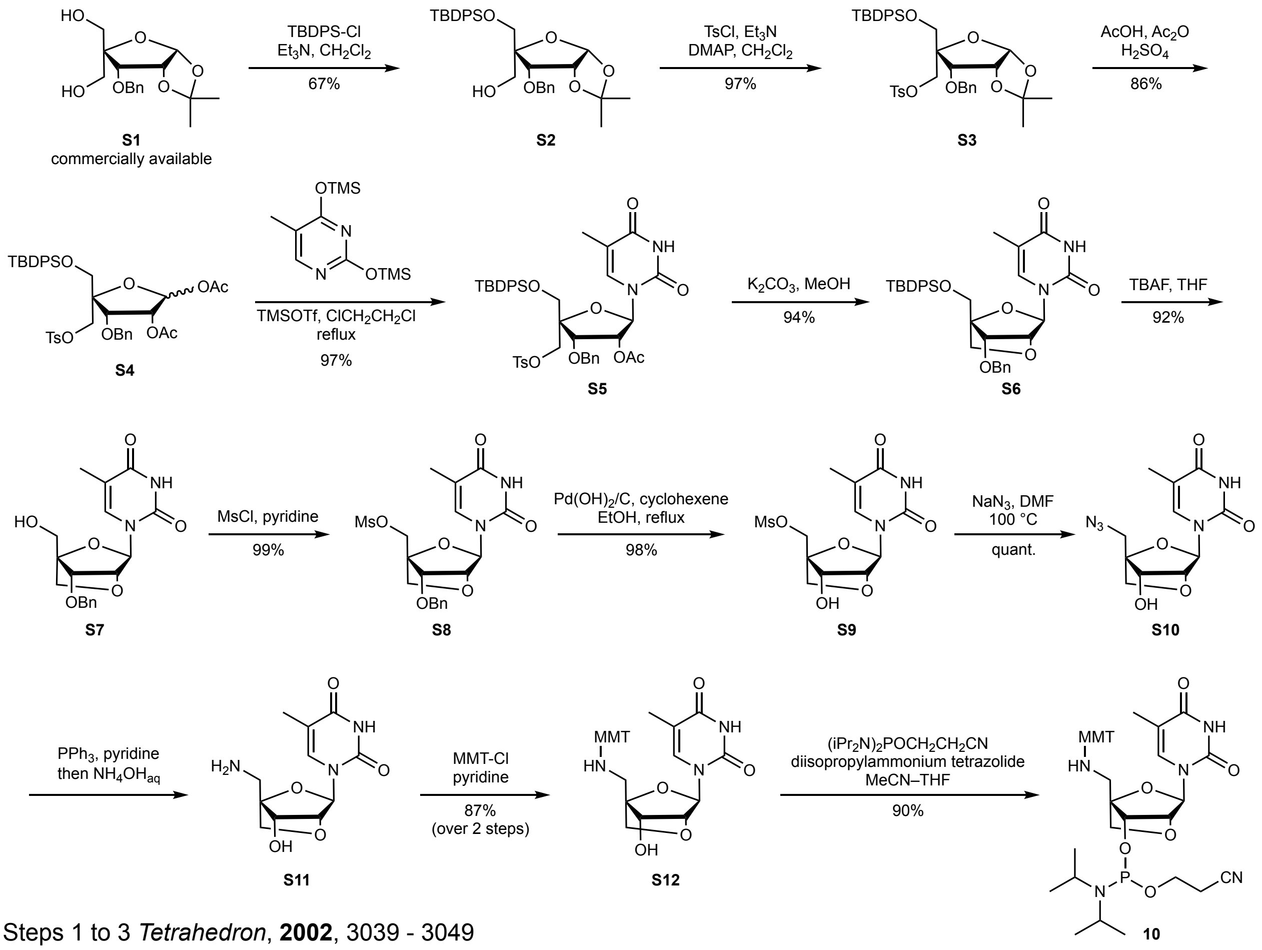

Steps 1 to 3 *Tetrahedron*, **2002**, 3039 - 3049

Steps 4 to 12 Chem Commmun., **2003**, 2202 - 2203

Our improved route via Koshkin route to intermediate S8

45% overall yield in 7 steps from commercially available starting material

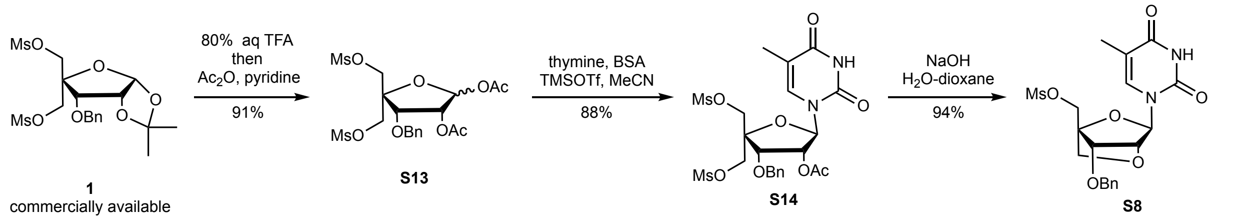

Steps 1 to 3 previously reported by Koshkin et al J. *Org. Chem.* **2001**, *66*, 8504 – 8512

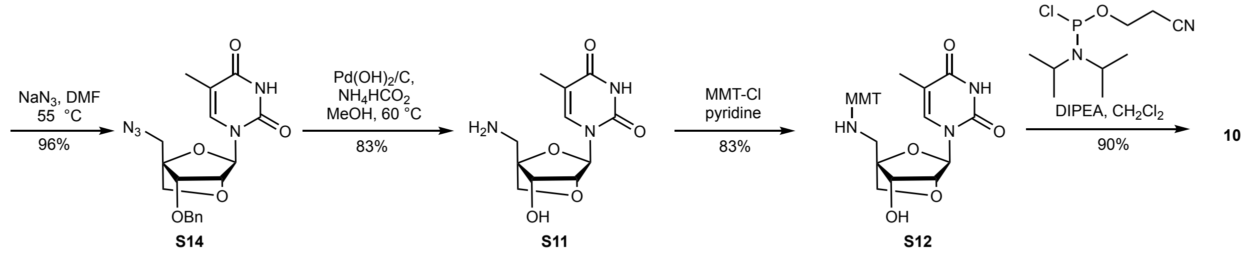

Supplementary Figure 2. Synthesis route used for 5´-amino LNA phosphoramidite **10** *via* the synthesis for **S8** reported by Koskin *et al.*[1] and how it compares with the route previously reported by Obika *et al.*[2,3] We have previously reported the synthesis of **S14** and **S11**[4], but have now reduced the equivalents of NaN_3 from 7 to 2 during the S_N2 step and have identified conditions to reduce the azide and cleave the benzyl in a single step, improving the scalability of the synthesis.

UPLC and MS analysis of oligonucleotides with LNA-amide linkages

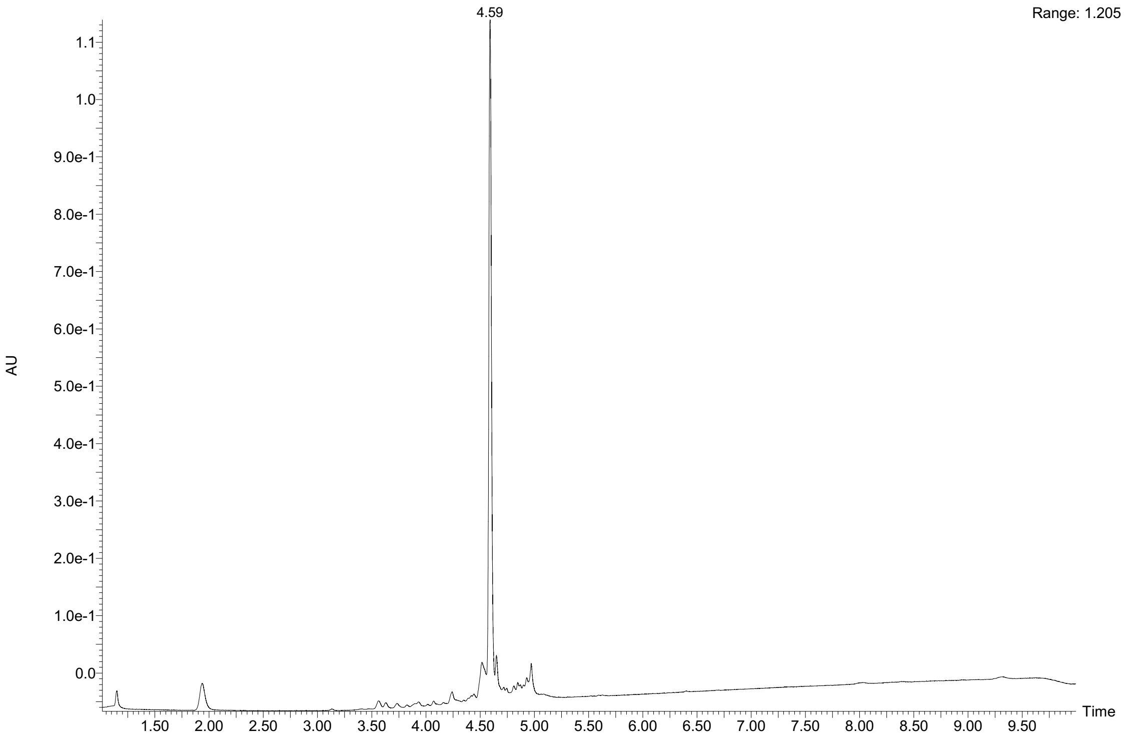

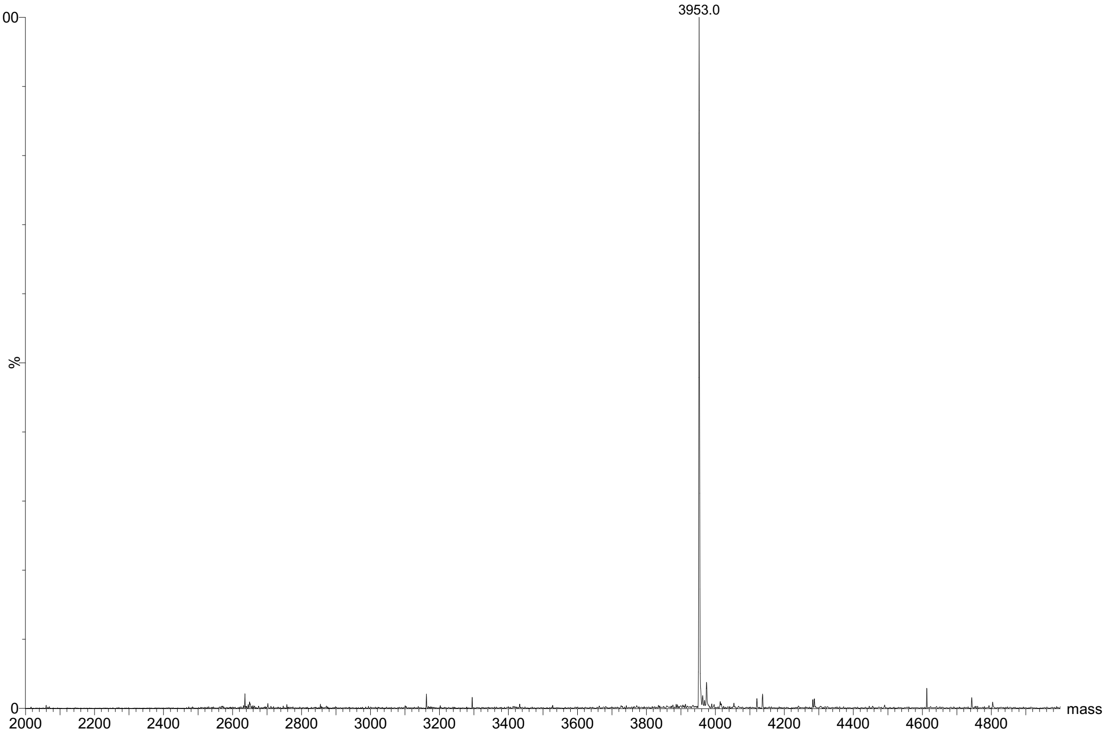

Supplementary Figure 3. Reverse-phase UPLC (UV absorbance at 260 nm vs time in min) and mass spectrum (ES^-) of $ON4^{LNA\text{-}Am\text{-}LNA}$ without purification. Required 3952.9 Da, found 3953.0 Da. CGACGC**T***<u>**T**</u>GCAGC. y-axis = relative intensity (%), x-axis = mass in Da. This demonstrates that compound **9a** is compatible with solid phase oligonucleotide synthesis.

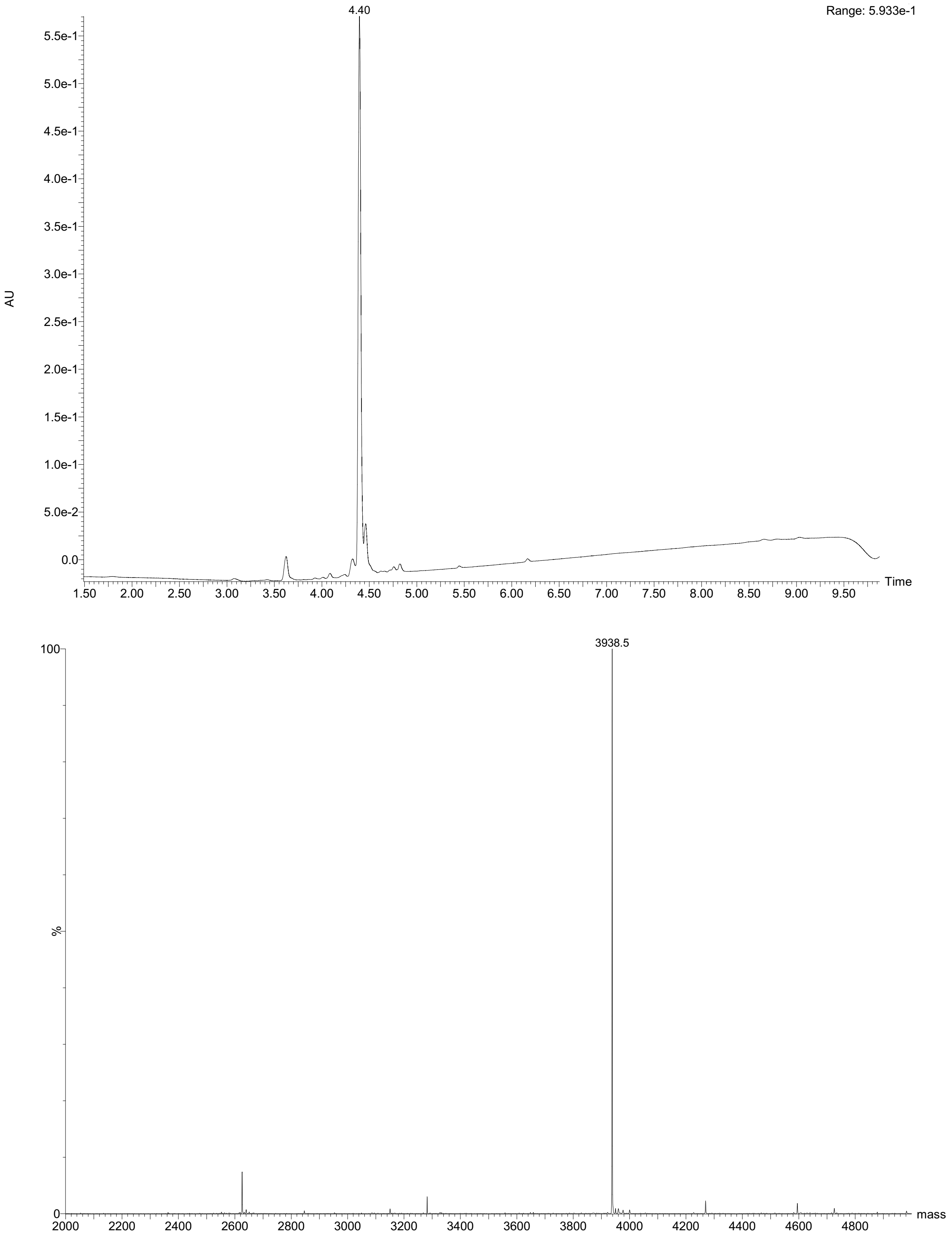

Supplementary Figure 4. Reverse-phase UPLC (UV absorbance at 260 nm vs time in min) and mass spectrum (ES^-) of ONS1 without purification. Required 3937.8 Da, found 3938.5 Da. CGACGCC*TGCAGC. y-axis = relative intensity (%), x-axis = mass in Da. This demonstrates that compound **9b** is compatible with solid phase oligonucleotide synthesis.

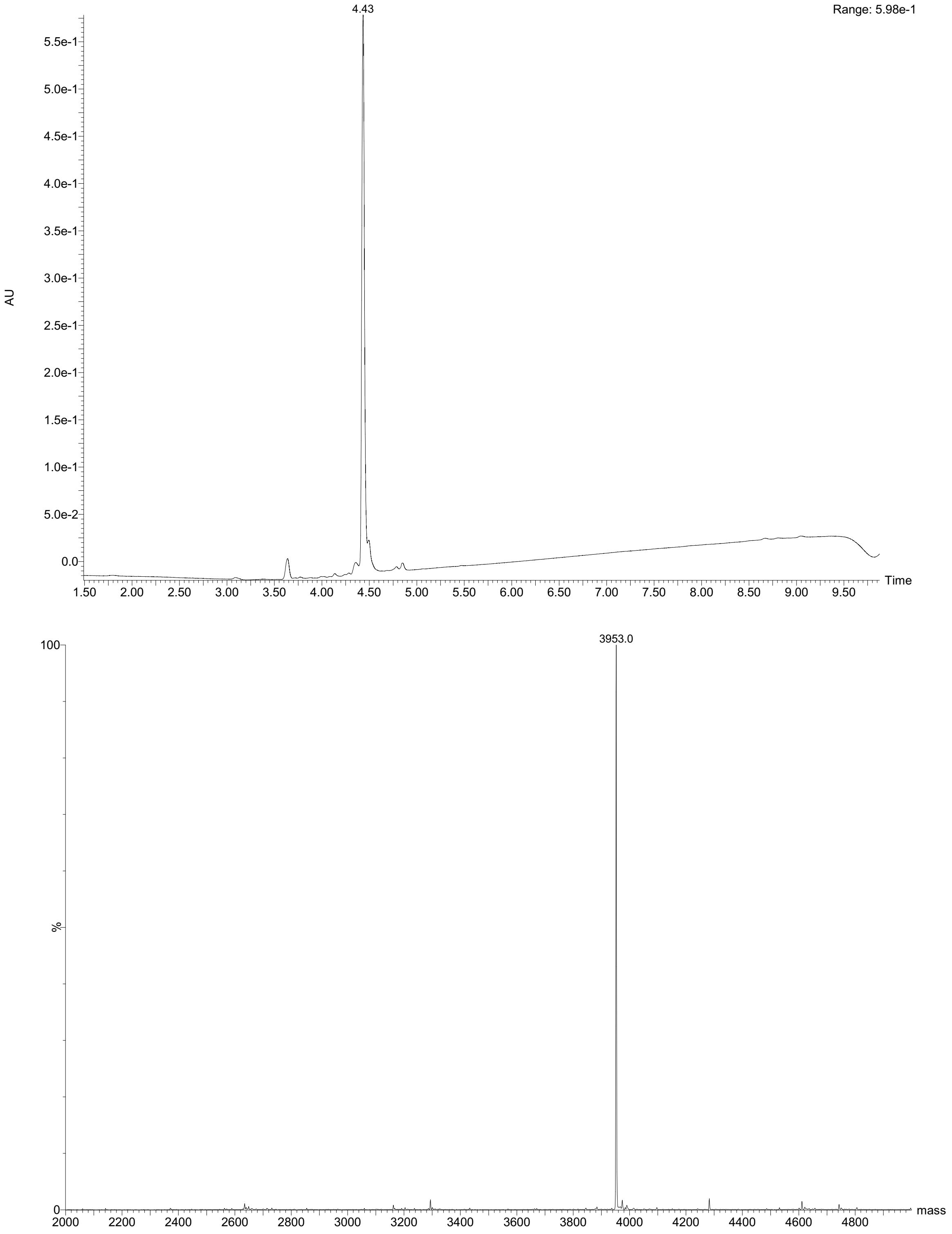

Supplementary Figure 5. Reverse-phase UPLC (UV absorbance at 260 nm vs time in min) and mass spectrum (ES$^-$) of ONS2 without purification. Required 3951.9 Da, found 3953.0 Da. CGACGC^{Me}C*TGCAGC. y-axis = relative intensity (%), x-axis = mass in Da. This demonstrates that compound **9c** is compatible with solid phase oligonucleotide synthesis.

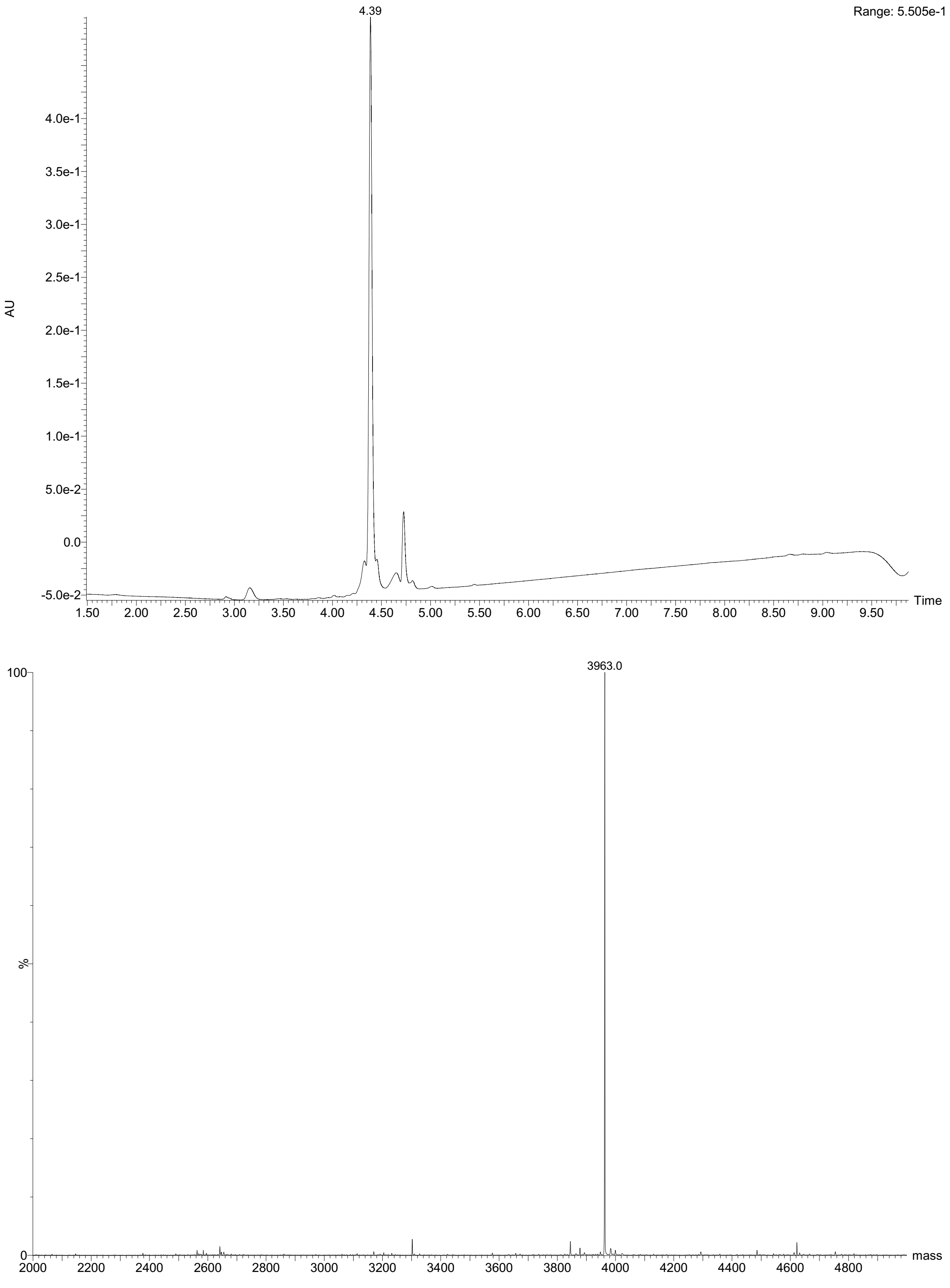

Supplementary Figure 6. Reverse-phase UPLC (UV absorbance at 260 nm vs time in min) and mass spectrum (ES$^-$) of ONS3 without purification. Required 3961.9 Da, found 3963.0 Da. CGACGCA*TGCAGC. y-axis = relative intensity (%), x-axis = mass in Da. This demonstrates that compound **9d** is compatible with solid phase oligonucleotide synthesis.

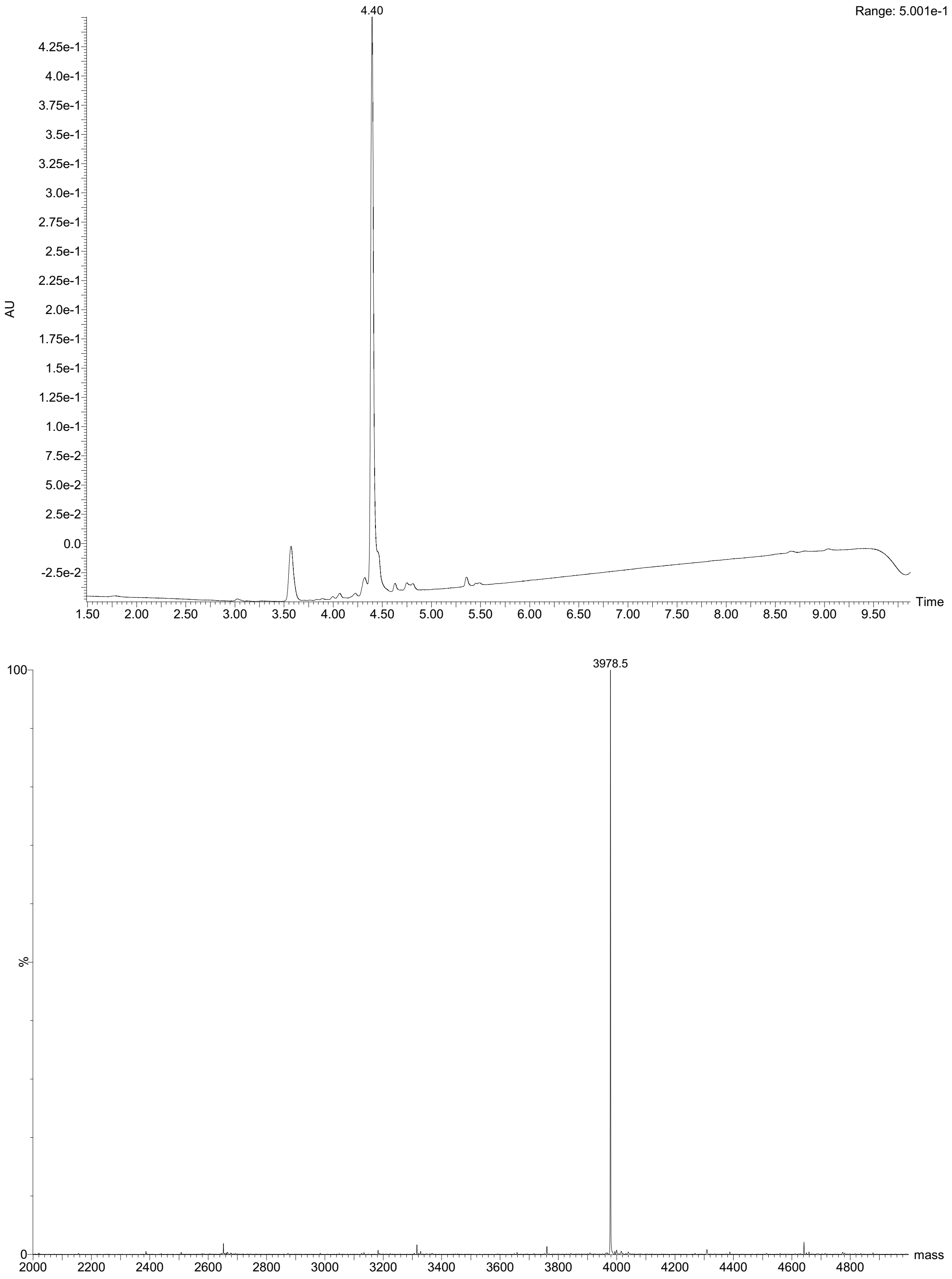

Supplementary Figure 7. Reverse-phase UPLC (UV absorbance at 260 nm vs time in min) and mass spectrum (ES^{-}) of ONS4 without purification. Required 3977.9 Da, found 3978.5 Da. CGACGCG*TGCAGC. y-axis = relative intensity (%), x-axis = mass in Da. This demonstrates that compound **9e** is compatible with solid phase oligonucleotide synthesis.

Representative UV melting curves

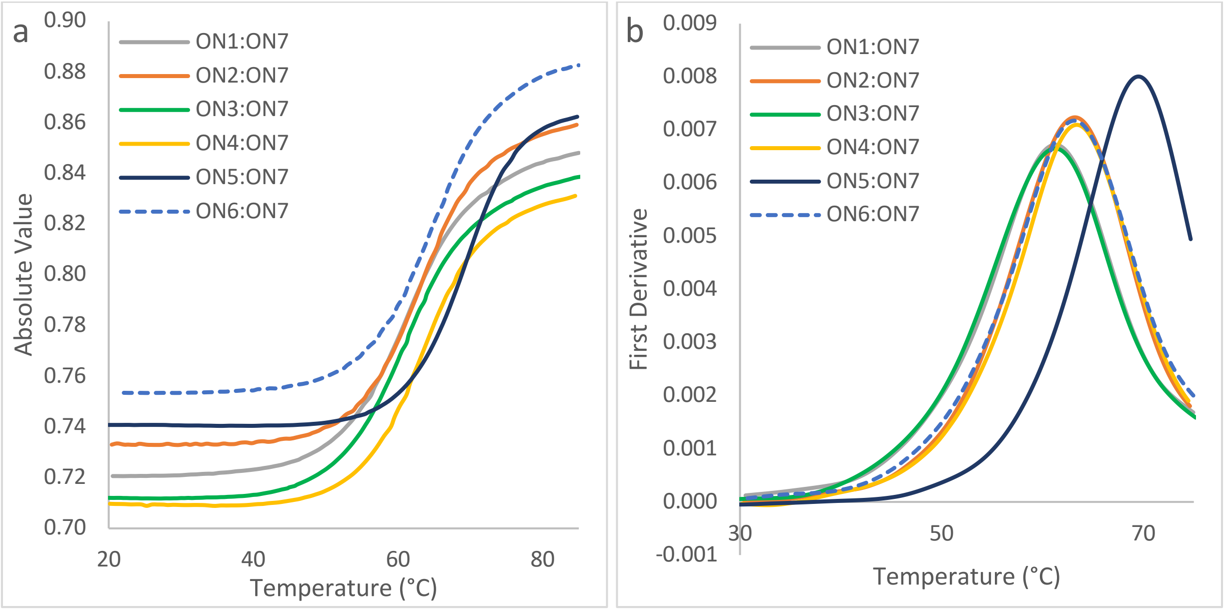

Supplementary Figure 8. UV melting studies for modified ONs against complementary DNA (ON7) (Supplementary Table 2). **a.** Representative UV melting curves measured using 3 µM of each ON in pH 7.0 phosphate buffer containing 200 mM NaCl; **b.** 1st derivative of melting curves. The curves shown are representative of three independent repeats, each consisting of at least three technical repeats.

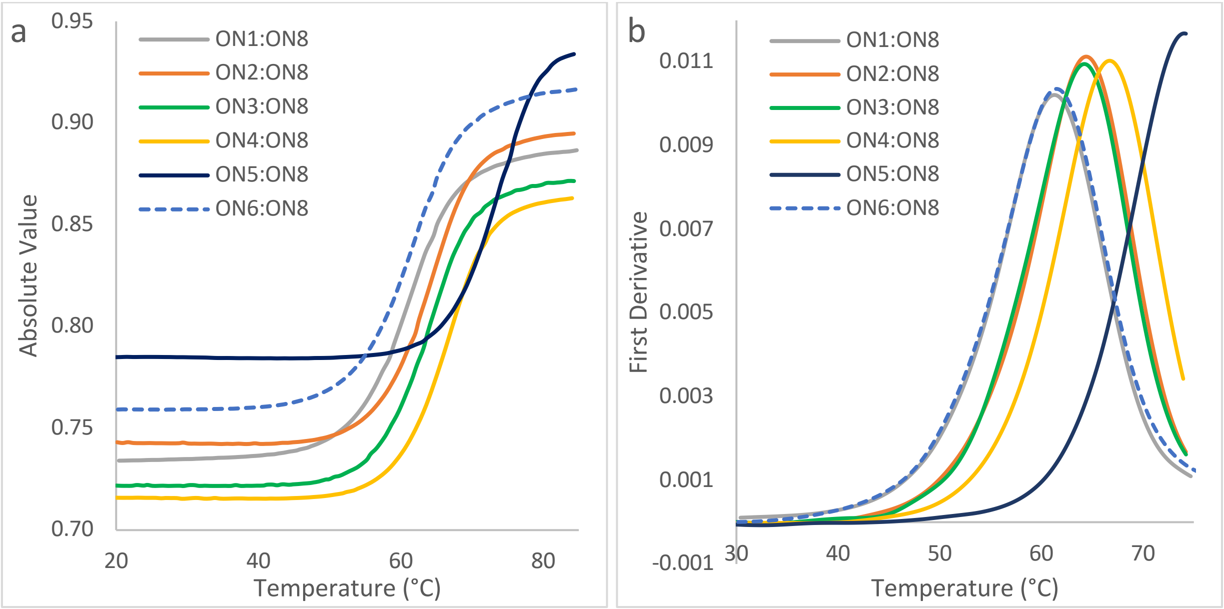

Supplementary Figure 9. UV melting studies for modified ONs against complementary RNA (ON8) (Supplementary Table 2). **a.** Representative UV melting curves measured using 3 µM of each ON in pH 7.0 phosphate buffer containing 200 mM NaCl; **b.** 1st derivative of melting curves. The curves shown are representative of three independent repeats, each consisting of at least three technical repeats.

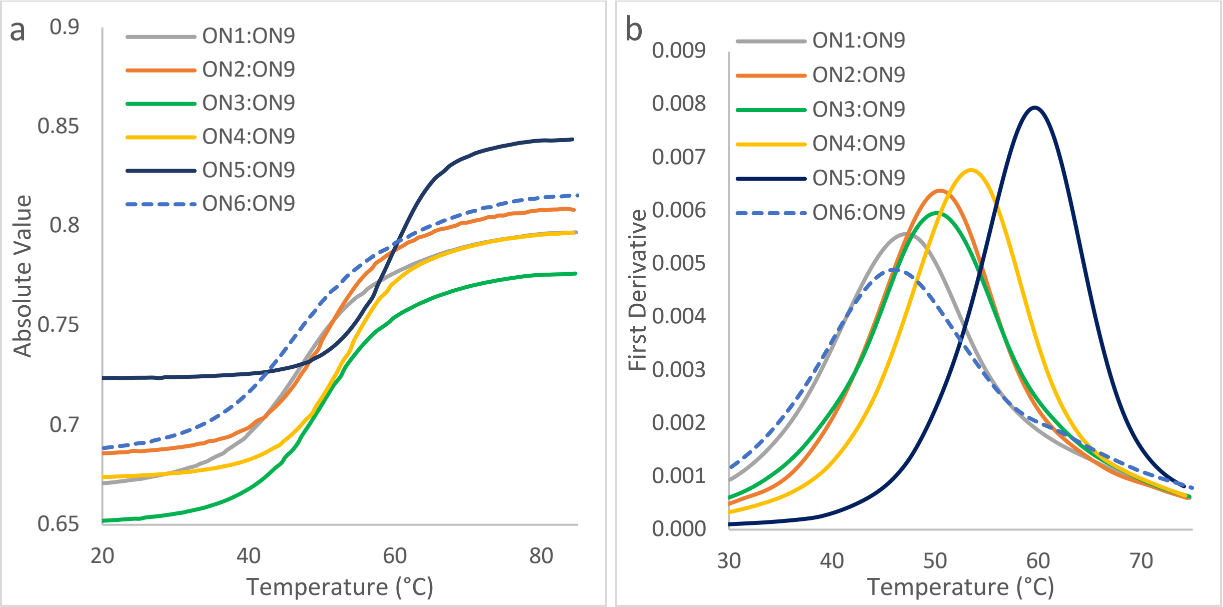

Supplementary Figure 10. UV melting studies for modified ONs against RNA with a C mismatch 5′ of the amide (ON9) (Supplementary Table 2). **a.** Representative UV melting curves measured using 3 µM of each ON in pH 7.0 phosphate buffer containing 200 mM NaCl; **b.** 1st derivative of melting curves. The curves shown are representative of three independent repeats, each consisting of at least three technical repeats.

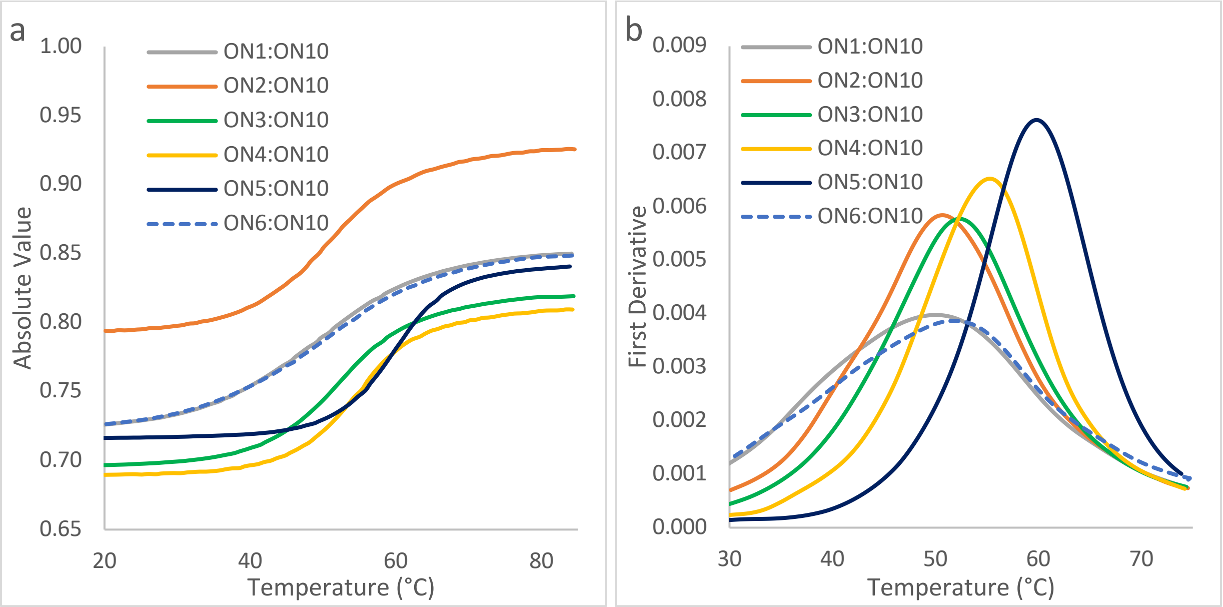

Supplementary Figure 11. UV melting studies for modified ONs against RNA with a C mismatch 3′ of the amide (ON10) (Supplementary Table 2). **a.** Representative UV melting curves measured using 3 µM of each ON in pH 7.0 phosphate buffer containing 200 mM NaCl; **b.** 1st derivative of melting curves. The curves shown are representative of three independent repeats, each consisting of at least three technical repeats.

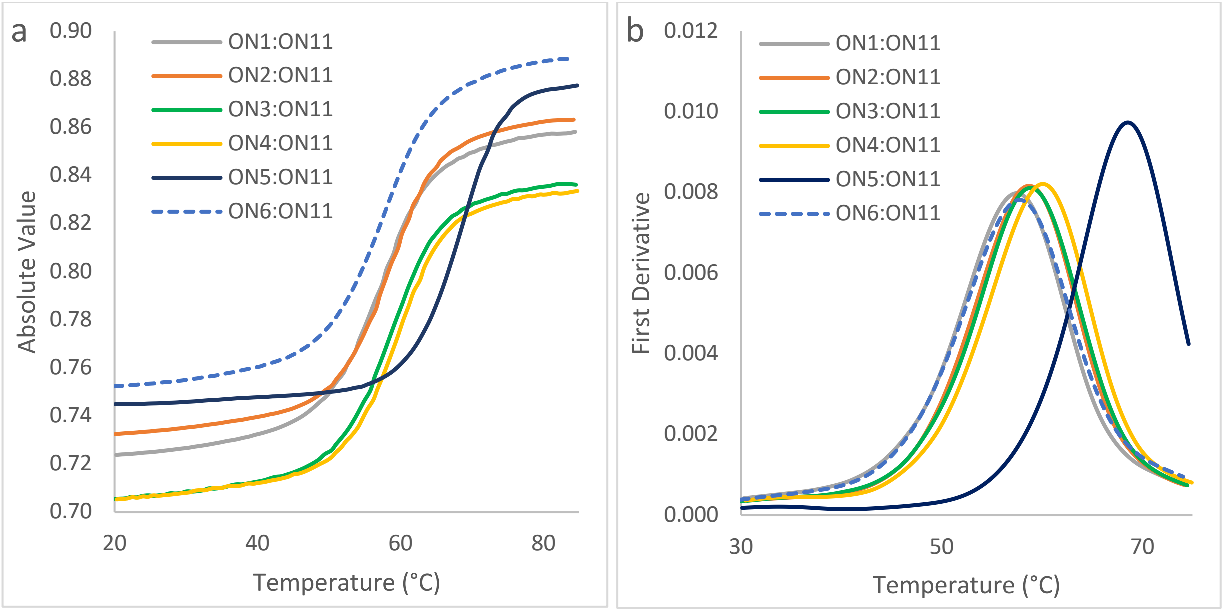

Supplementary Figure 12. UV melting studies for modified ONs against RNA with a G mismatch 5′ of the amide (ON11) (Supplementary Table 2). **a.** Representative UV melting curves measured using 3 µM of each ON in pH 7.0 phosphate buffer containing 200 mM NaCl; **b.** 1st derivative of melting curves. The curves shown are representative of three independent repeats, each consisting of at least three technical repeats.

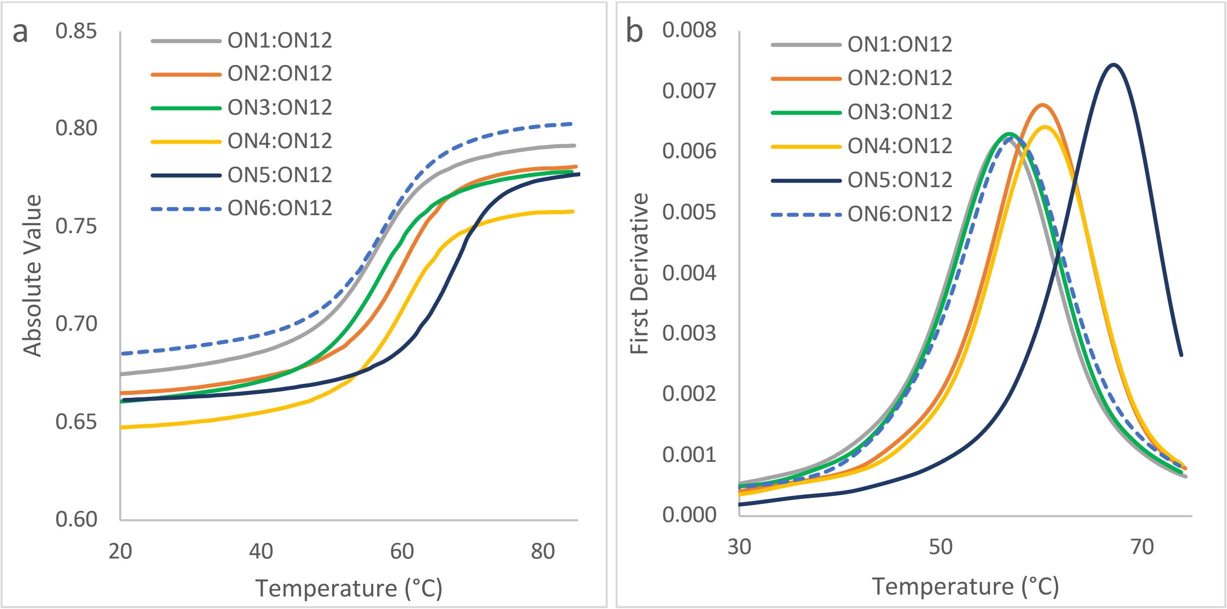

Supplementary Figure 13. UV melting studies for modified ONs against RNA with a G mismatch 3′ of the amide (ON12) (Supplementary Table 2). **a.** Representative UV melting curves measured using 3 µM of each ON in pH 7.0 phosphate buffer containing 200 mM NaCl; **b.** 1st derivative of melting curves. The curves shown are representative of three independent repeats, each consisting of at least three technical repeats.

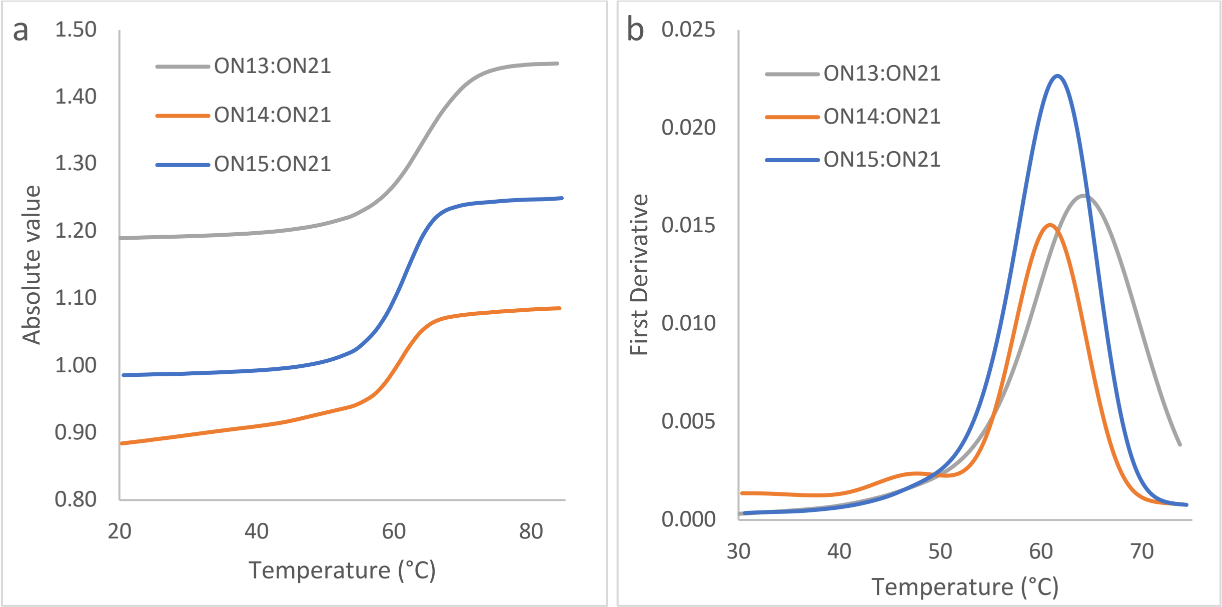

Supplementary Figure 14. UV melting studies comparing 0, 1 and 4 additions of LNA-amide against a complementary DNA target (ON21) (Supplementary Table 3). **a.** Representative UV melting curves measured using 3 µM of each ON in pH 7.0 phosphate buffer containing 200 mM NaCl; **b.** 1st derivative of melting curves. The curves shown are representative of three independent repeats, each consisting of at least three technical repeats.

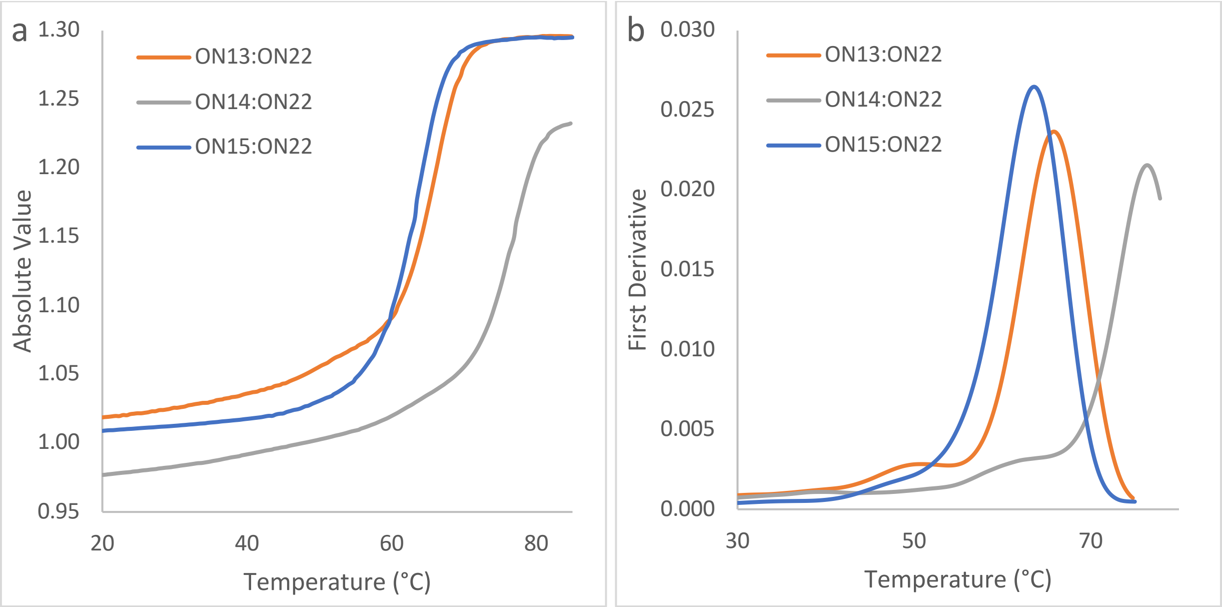

Supplementary Figure 15. UV melting studies comparing 0, 1 and 4 additions of LNA-amide against a complementary RNA target (ON22) (Supplementary Table 3). **a.** Representative UV melting curves measured using 3 µM of each ON in pH 7.0 phosphate buffer containing 200 mM NaCl; **b.** 1st derivative of melting curves. The curves shown are representative of three independent repeats, each consisting of at least three technical repeats.

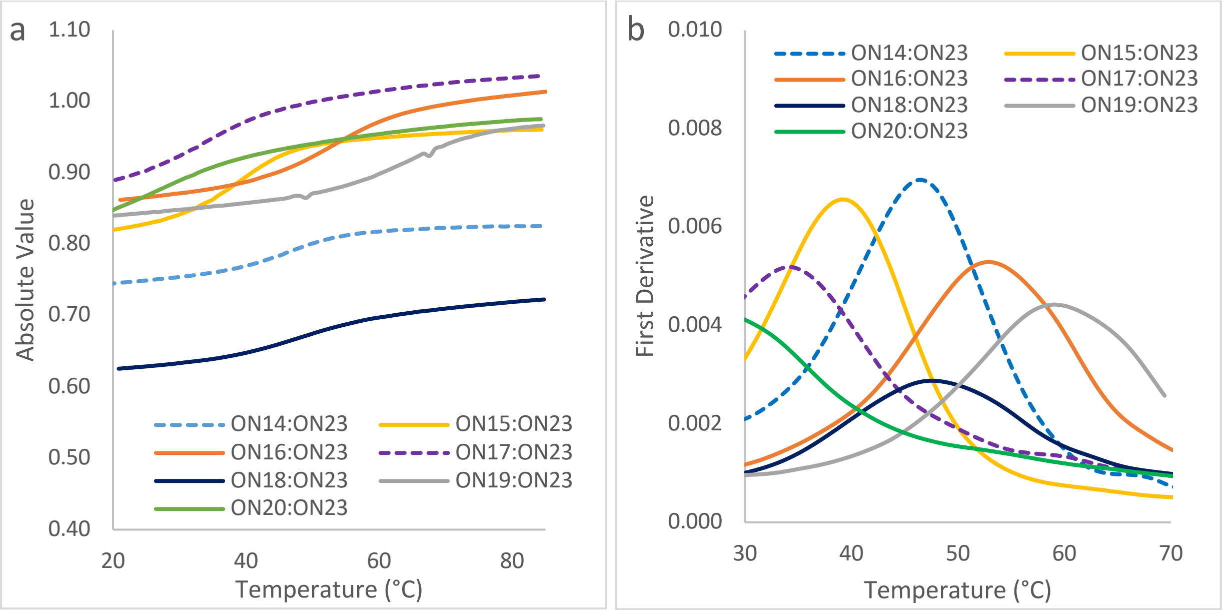

Supplementary Figure 16. UV melting studies for modified ONs against truncated DNA target ON23 (Supplementary Table 3). **a.** Representative UV melting curves measured using 3 µM of each ON in pH 7.0 phosphate buffer containing 200 mM NaCl; **b.** 1st derivative of melting curves. The complementary DNA target was truncated so that the melting temperatures were in the range of the instrument. The truncated DNA strand covers the critical modified region of the modified ONs. The curves shown are representative of three independent repeats, each consisting of at least three technical repeats.

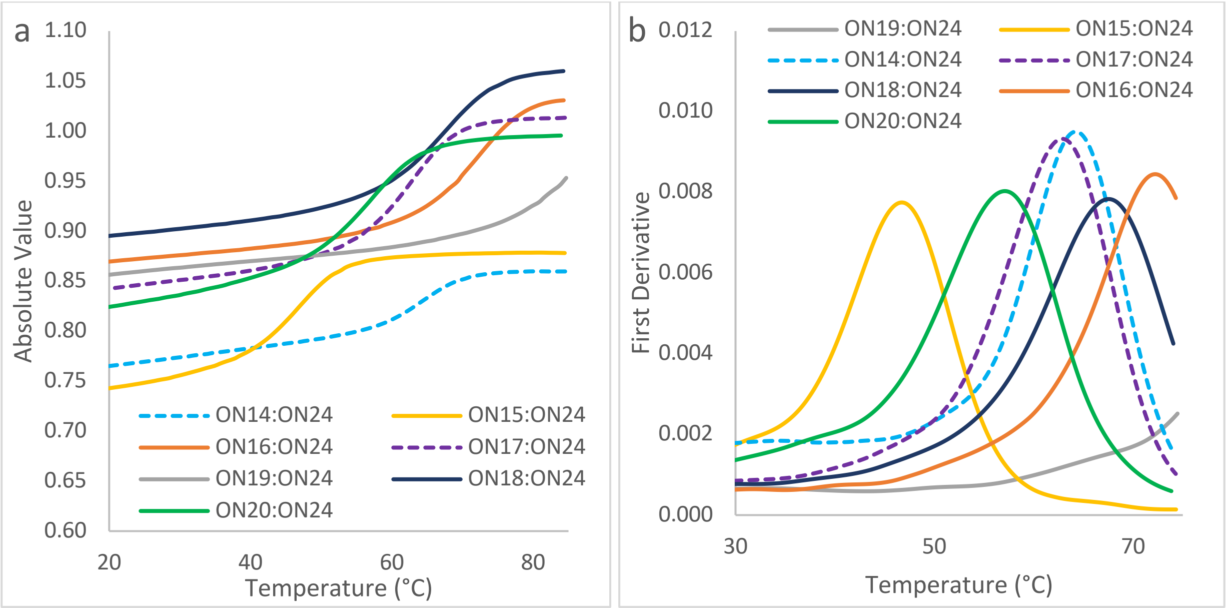

Supplementary Figure 17. UV melting studies for modified ONs against truncated RNA target ON24 (Supplementary Table 3). **a.** Representative UV melting curves measured using 3 µM of each ON in pH 7.0 phosphate buffer containing 200 mM NaCl; **b.** 1st derivative of melting curves. The complementary RNA target was truncated so that the melting temperatures were in the range of the instrument. The truncated RNA strand covers the critical modified region of the modified ONs. The curves shown are representative of three independent repeats, each consisting of at least three technical repeats.

Nuclease resistance supplementary figure

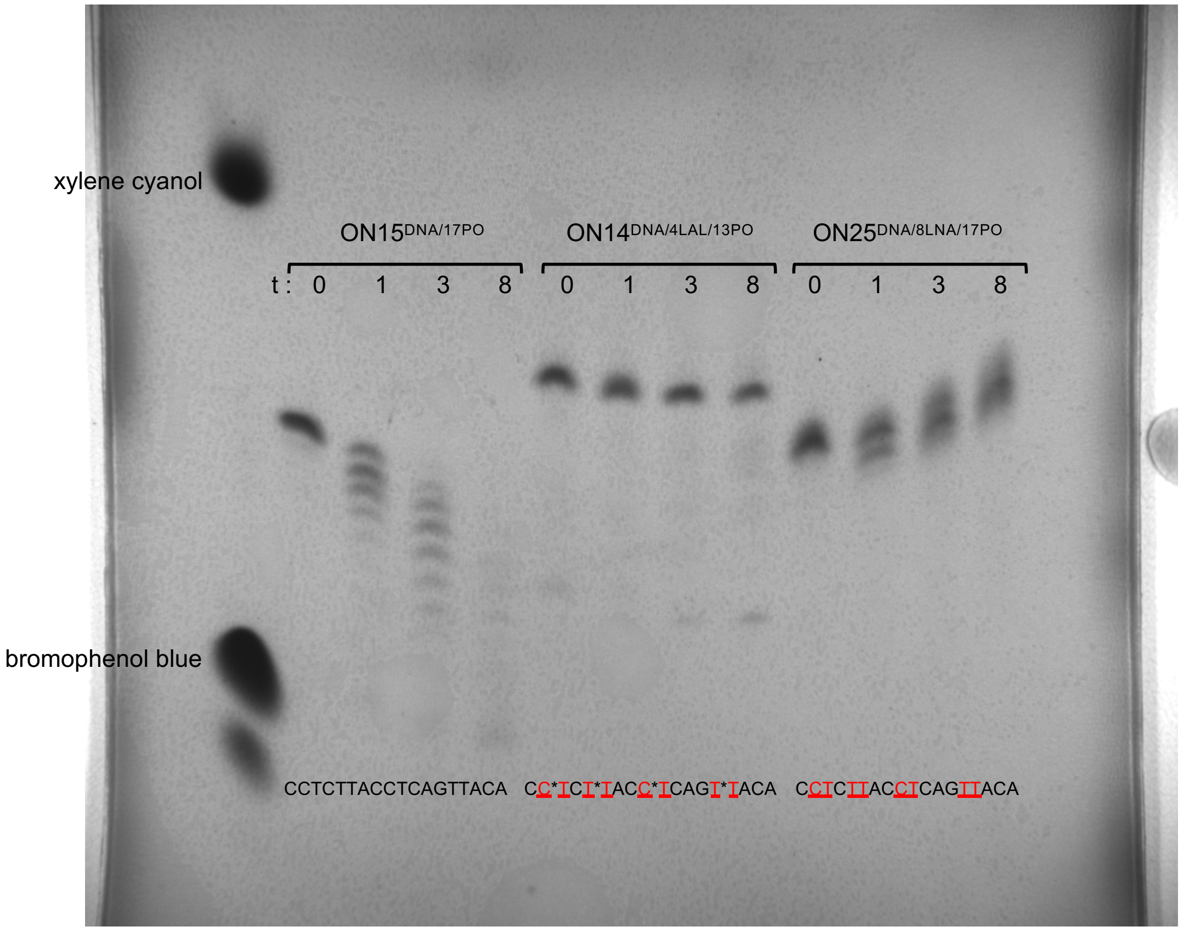

Supplementary Figure 18. Denaturing polyacrylamide gel electrophoresis (PAGE) analysis of modified ONs after incubation in FBS:PBS (1:1). t = incubation time in hours. Underlined red bases indicate a locked sugar and * is an amide bond in place of a phosphodiester. UV shadowing on a G:BOX gel Imager and GeneSnap 7.12 software (SynGene). This experiment was performed in duplicate with similar results.

XRD supplementary figures

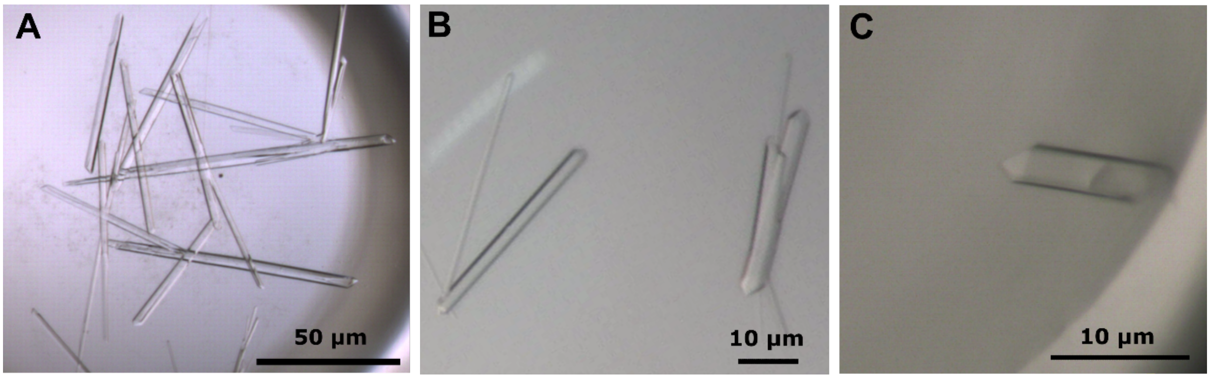

Supplementary Figure 19. Example of crystals obtained from vapor diffusion. A) ON26^{xDNA}:ON27^{xRNA}, B) ON29$^{xLNA\text{-}Am\text{-}DNA}$:ON27^{xRNA}, C) ON30$^{xLNA\text{-}Am\text{-}LNA}$:ON27^{xRNA}. These experiments were not replicated.

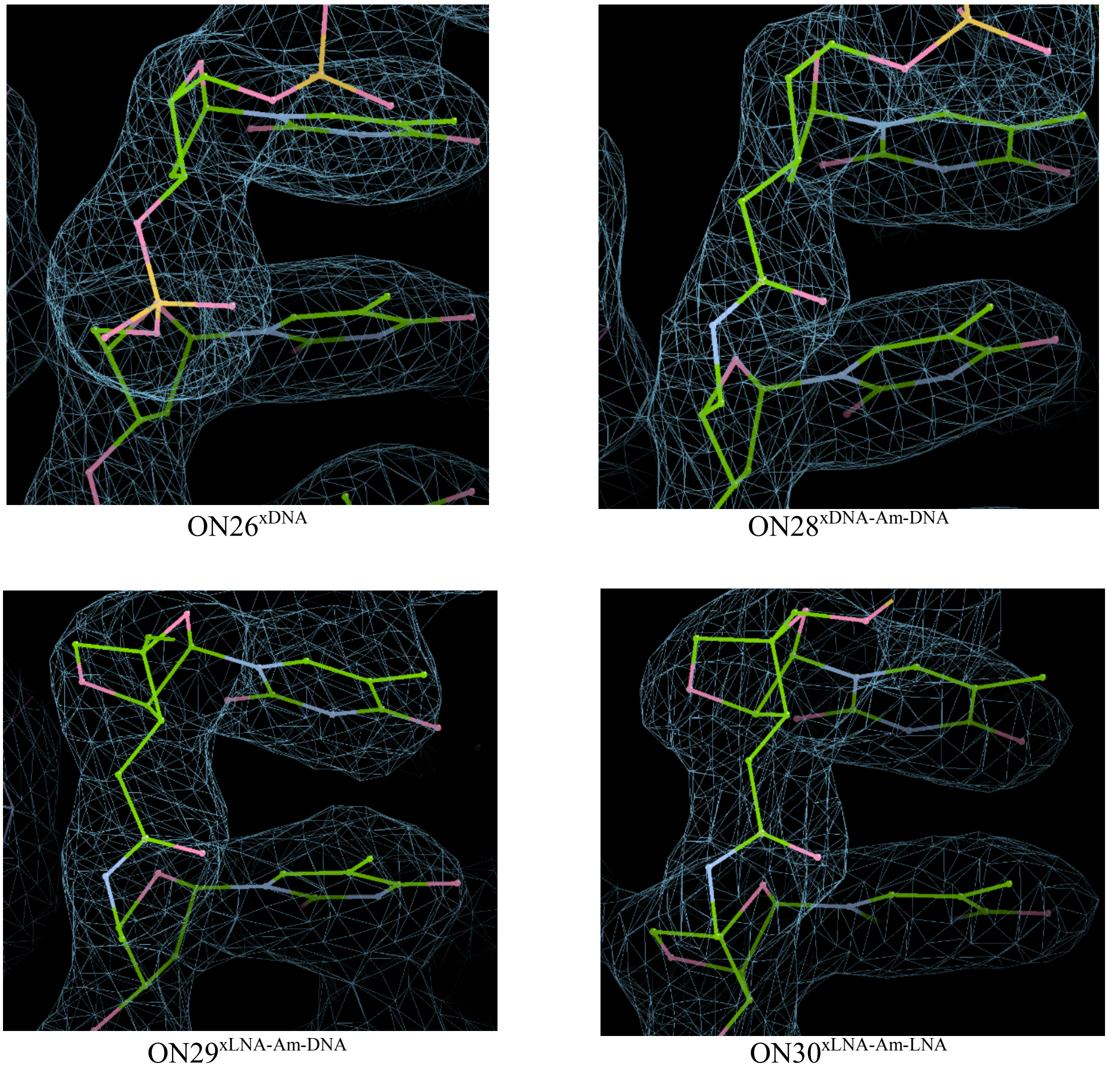

Supplementary Figure 20. $2F_o$-F_c electron density map (blue), at the 1σ contour level, centred on the unmodified phosphate (top left) or amide linkage for the four nucleic acid crystal structures reported in this manuscript. Atoms are coloured according to type with carbon in green, nitrogen in light blue and oxygen in red.

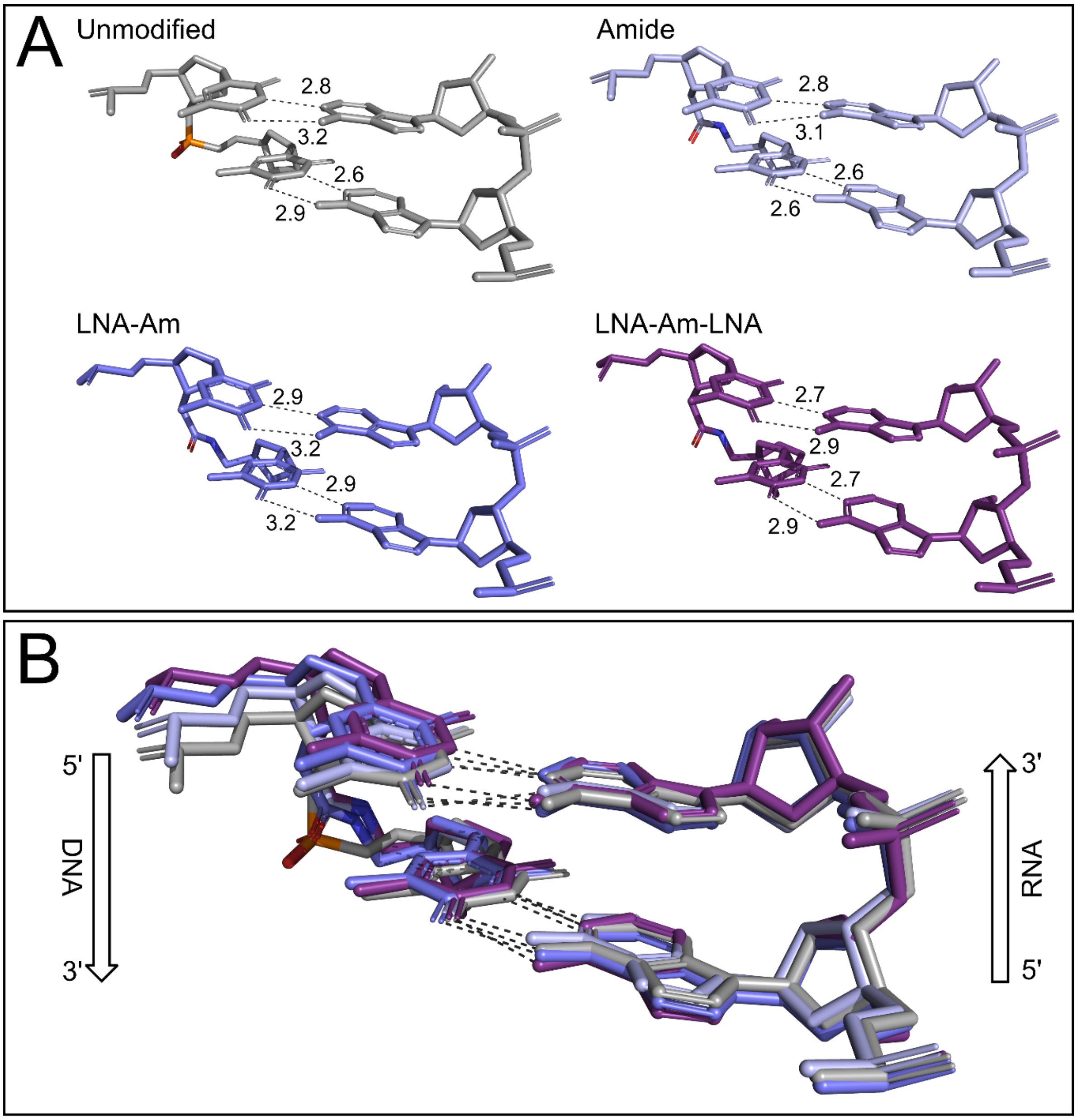

Supplementary Figure 21. A) Hydrogen-bonding interactions of the 7 A-T, 8 A-T base pair steps surrounding the site of modification. Unmodified ($ON26^{xDNA}$) = grey, amide ($ON28^{xDNA\text{-}Am\text{-}DNA}$) = light blue, LNA-Am ($ON29^{xLNA\text{-}Am\text{-}DNA}$) = dark blue and LNA-Am-LNA ($ON30^{xLNA\text{-}Am\text{-}LNA}$) = purple. Modifications are contained within the DNA strand (left side) and hydrogen bond distances between Watson-Crick base pairs are shown. B) Overlay of above structures showing base pair step structural similarity.

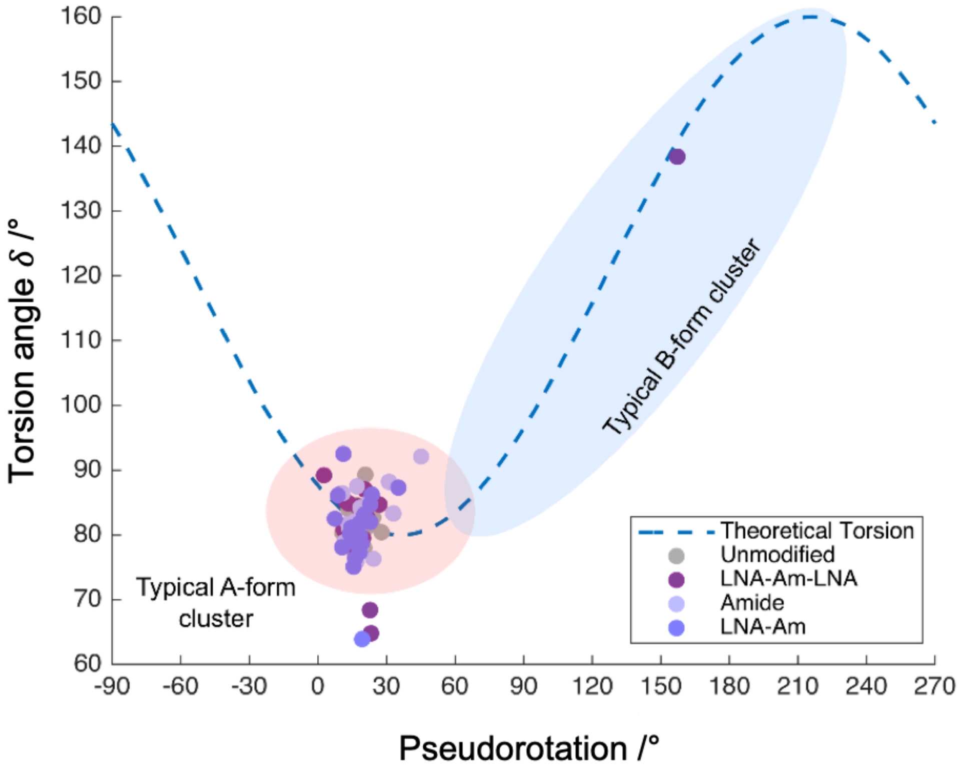

Supplementary Figure 22. Pseudorotation vs torsion angles of angle δ used to define sugar pucker conformations. Torsion data points were calculated using w3DNA 2.0 software and each point represents a single sugar conformation within a corresponding duplex. Each duplex has 20 data points and the clustering of these points can be interpreted to determine duplex form. The theoretical torsion angle is represented by equation ($\delta = 40\cos(P + 144) + 120$). In general, A-form duplexes have consistent pseudorotations 0-60° known as 3'-*endo* conformation. B-form duplexes have a larger distribution of pseudorotations 0-240°. Legend) Unmodified = ON26^{xDNA}:ON27^{xRNA}; LNA-Am-LNA = ON30$^{xLNA-Am-LNA}$:ON27^{xRNA}; Amide = ON28$^{xDNA-Am-DNA}$:ON27^{xRNA} ; LNA-Am = ON29$^{xLNA-Am-DNA}$:ON27^{xRNA}.

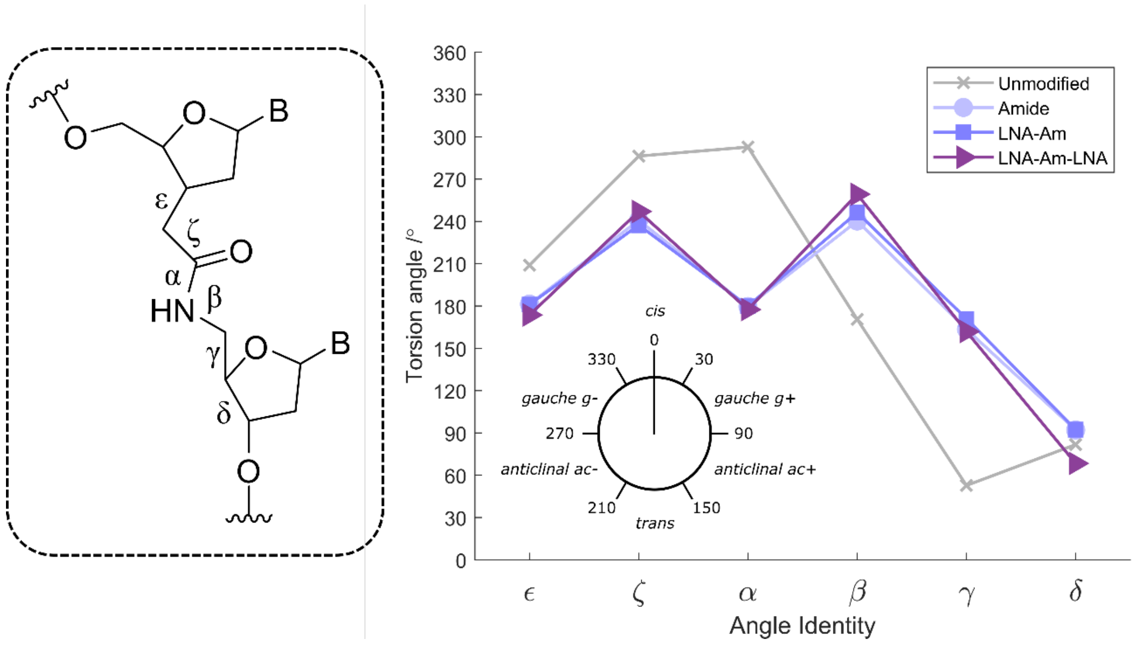

Supplementary Figure 23. Torsion angle comparison between amide modified backbones. Specific torsions are given by their respective symbol (ε→δ, right). Modified backbones are located at the 7A-T base pair step. Also included is the 7A-T phosphodiester step from the unmodified duplex. In general, all amide backbones, regardless of LNA modification have similar torsion angles. Legend) Unmodified = ON26^{xDNA}:ON27^{xRNA}; LNA-Am-LNA = ON30$^{xLNA\text{-}Am\text{-}LNA}$:ON27^{xRNA}; Amide = ON28$^{xDNA\text{-}Am\text{-}DNA}$:ON27^{xRNA} ; LNA-Am = ON29$^{xLNA\text{-}Am\text{-}DNA}$:ON27^{xRNA}.

Supplementary cell assay figures

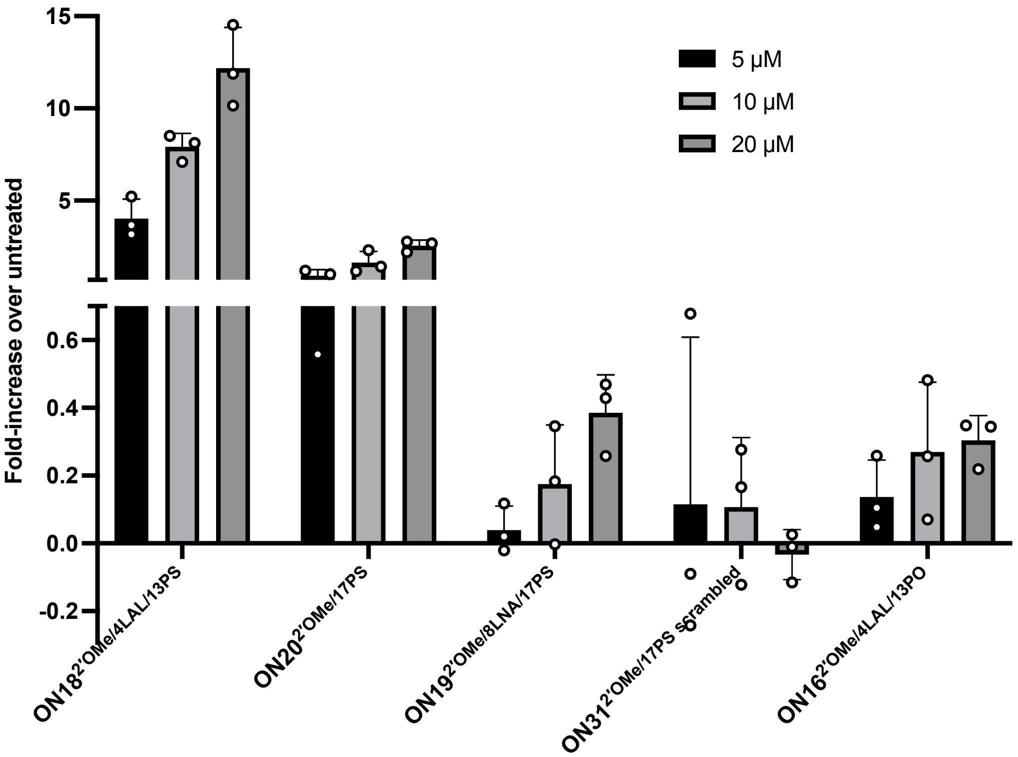

Supplementary Figure 24. LNA-flanked amides increase the gymnotic delivery and activity of ONs that contain PS linkages. Luciferase activity was measured and normalised to both protein quantity and untreated cells. Data are means ± standard deviation. Each dot represents one distinct biological replicate (n = 3). Source data are provided in the Source Data file.

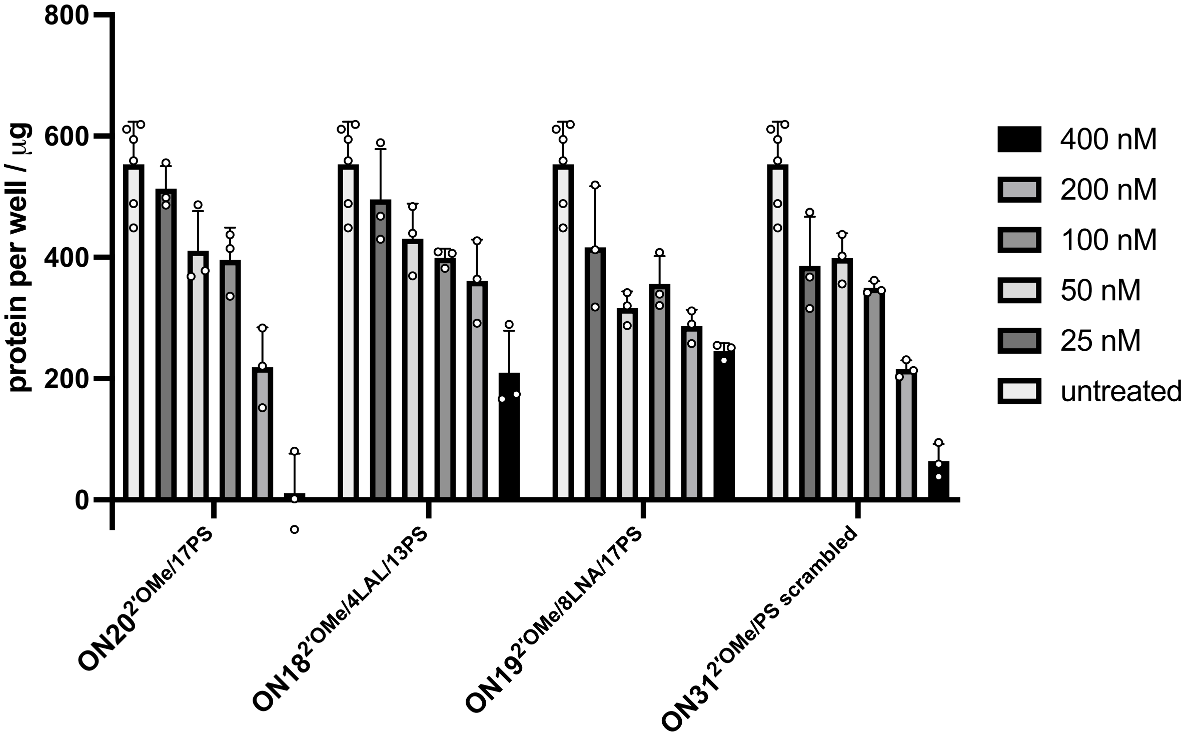

Supplementary Figure 25. Amount of protein per well 24 h after pLuc705 HeLa cells were transfected with ONs using lipofectamine 2000 (LF2000) at the concentrations indicated. A significant drop in protein was observed at higher concentrations indicating toxicity. Higher protein was observed for the amide modified ON, suggesting that LNA-flanked amides reduce toxicity of ONs transfected with LF2000. Data are means ± standard deviation. Each dot represents one distinct biological replicate (n = 3). Source data are provided in the Source Data file.

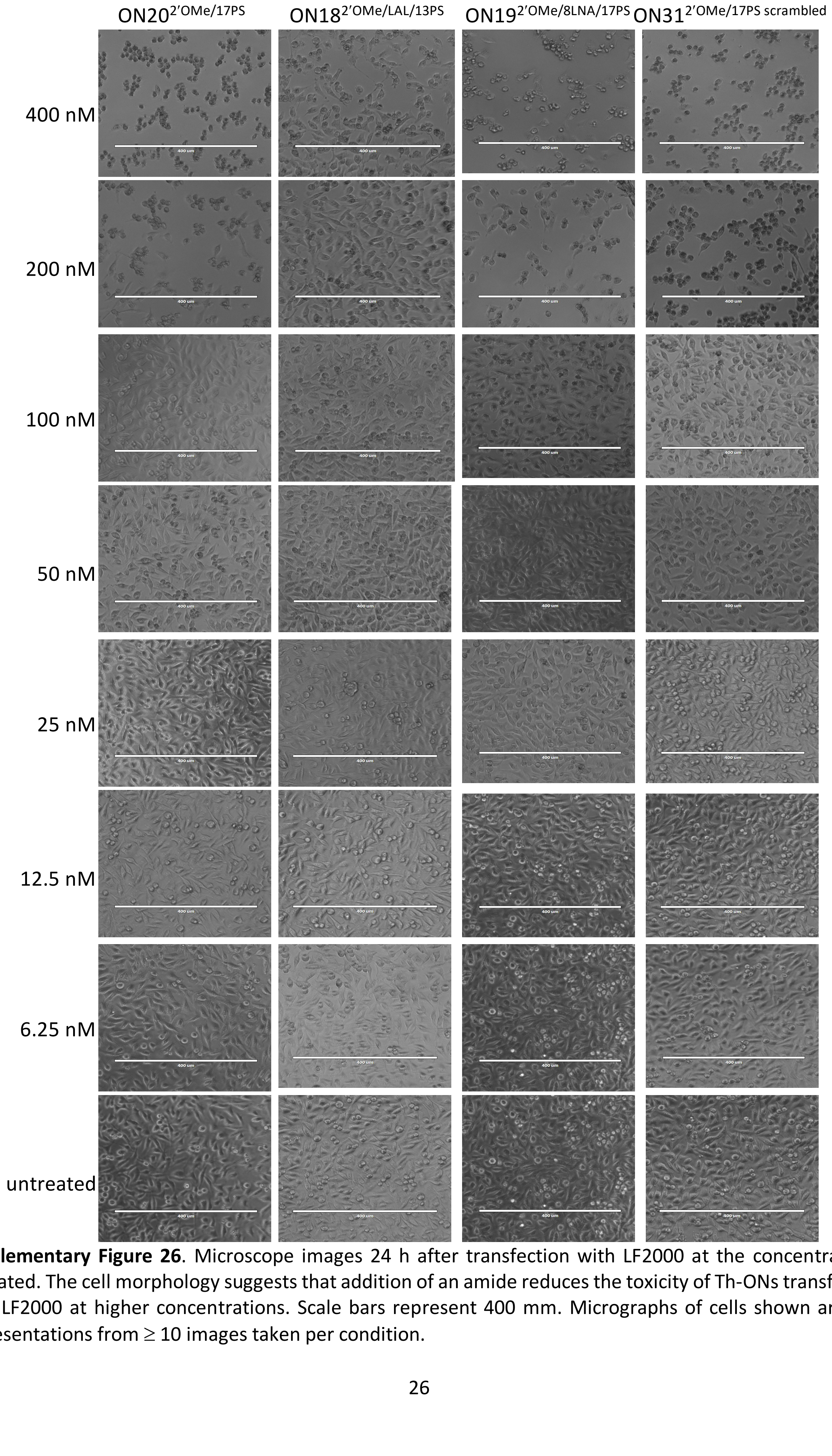

Supplementary Figure 26. Microscope images 24 h after transfection with LF2000 at the concentrations indicated. The cell morphology suggests that addition of an amide reduces the toxicity of Th-ONs transfected with LF2000 at higher concentrations. Scale bars represent 400 mm. Micrographs of cells shown are the representations from ≥ 10 images taken per condition.

UPLC-MS traces of LNA-amide modified oligonucleotides

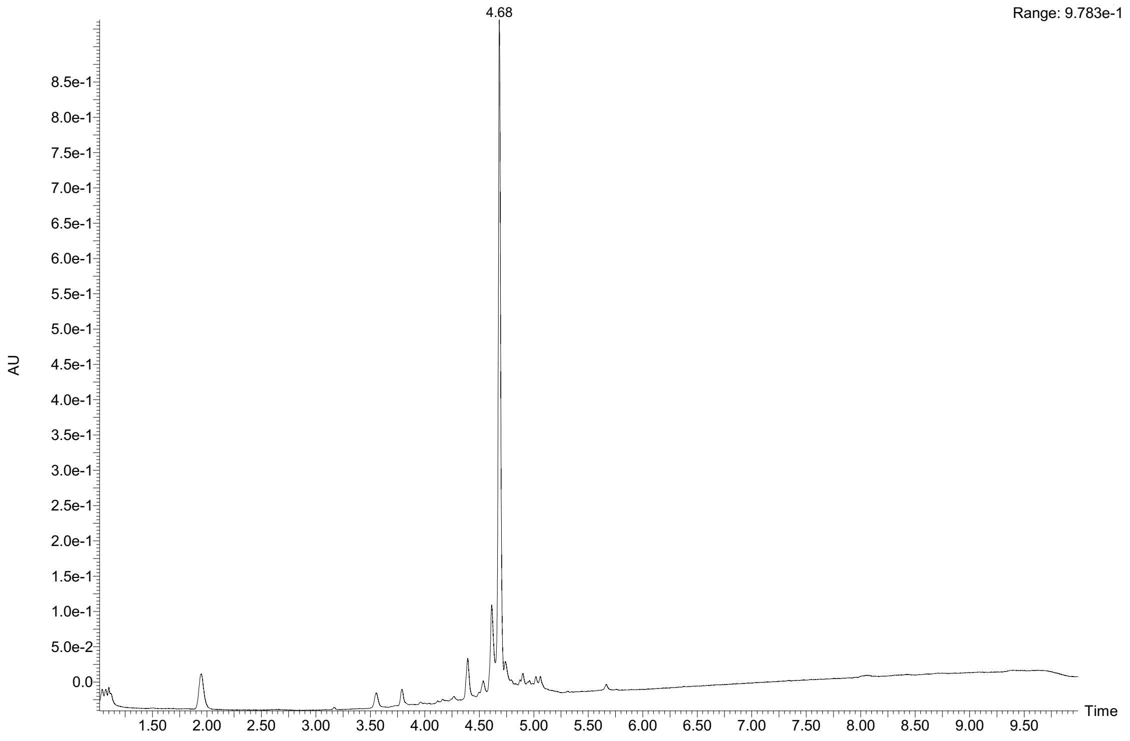

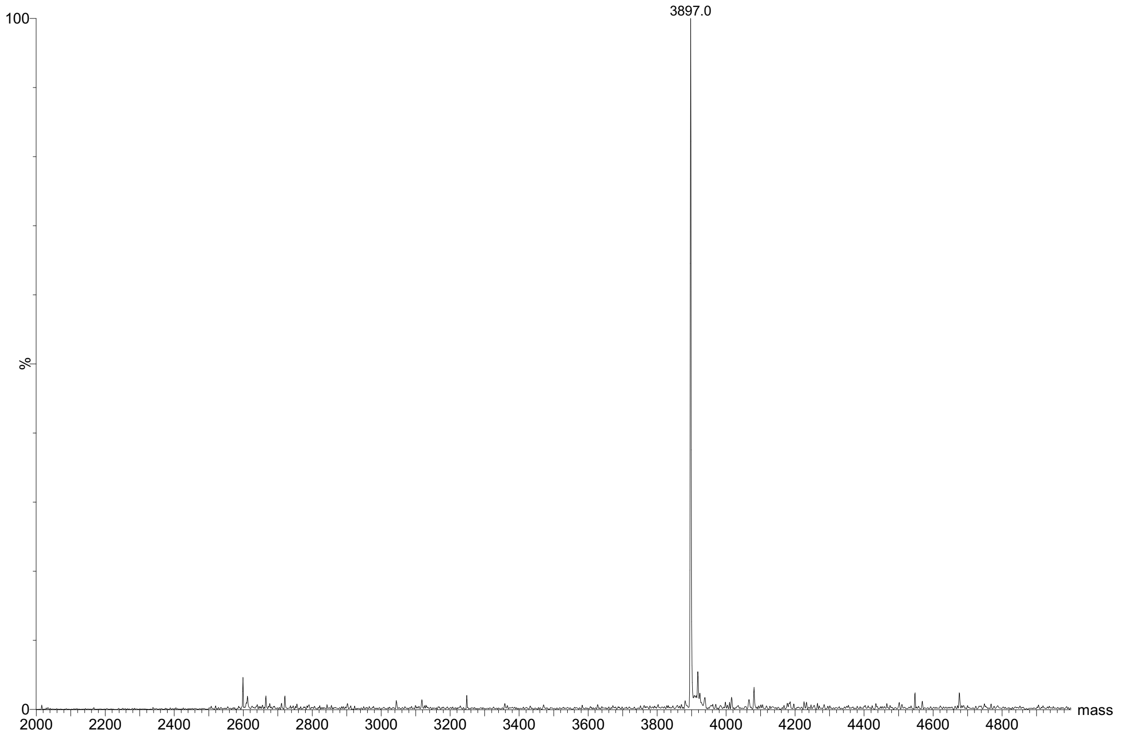

Supplementary Figure 27. Reverse-phase UPLC (UV absorbance at 260 nm vs time in min) and mass spectrum (ES$^-$) of ON1$^{DNA\text{-}Am\text{-}DNA}$ without purification. Required 3896.7 Da, found 3897.0 Da. CGACGCT*TGCAGC (DNA, phosphodiester backbone). y-axis = relative intensity (%), x-axis = mass in Da.

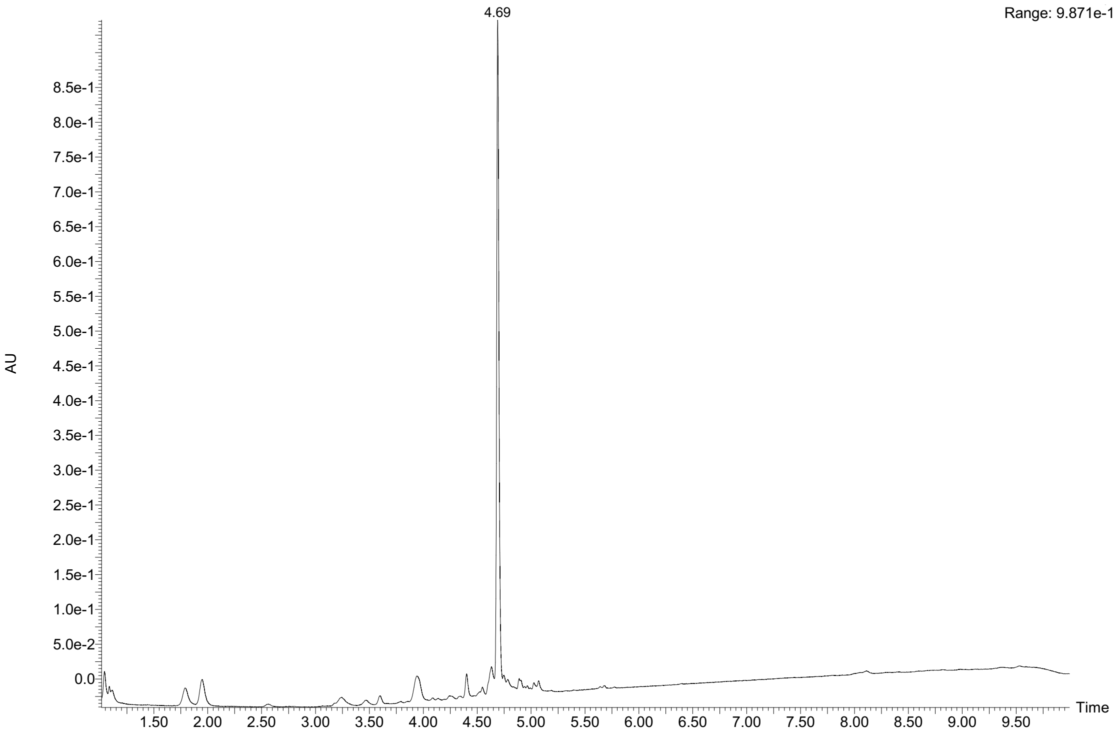

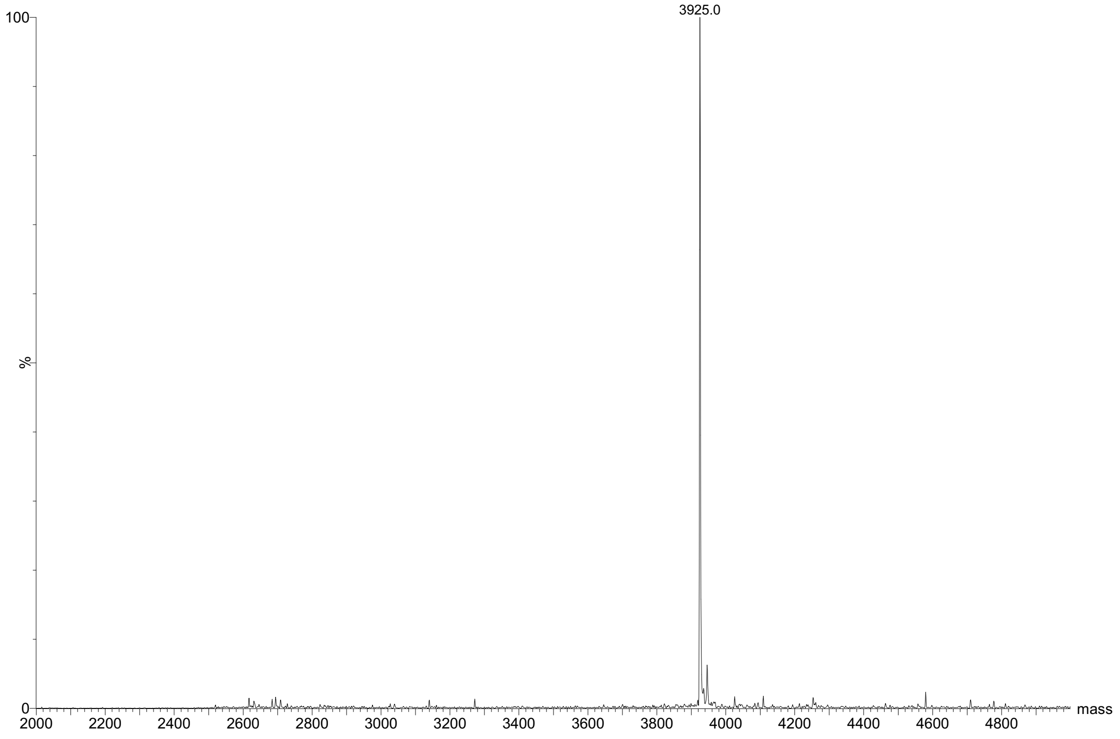

Supplementary Figure 28. Reverse-phase UPLC (UV absorbance at 260 nm vs time in min) and mass spectrum (ES$^-$) of ON2$^{LNA\text{-}Am\text{-}DNA}$ without purification. Required 3924.8 Da, found 3925.0 Da. CGACGC**T***TGCAGC (DNA, phosphodiester backbone). y-axis = relative intensity (%), x-axis = mass in Da.

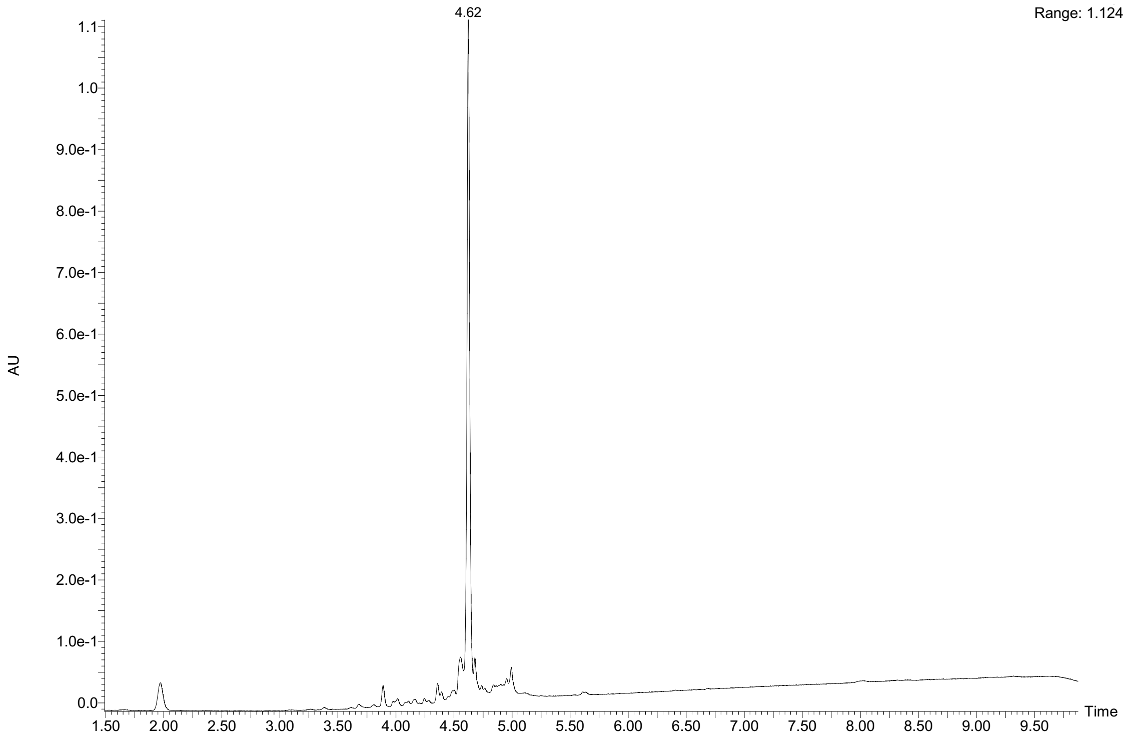

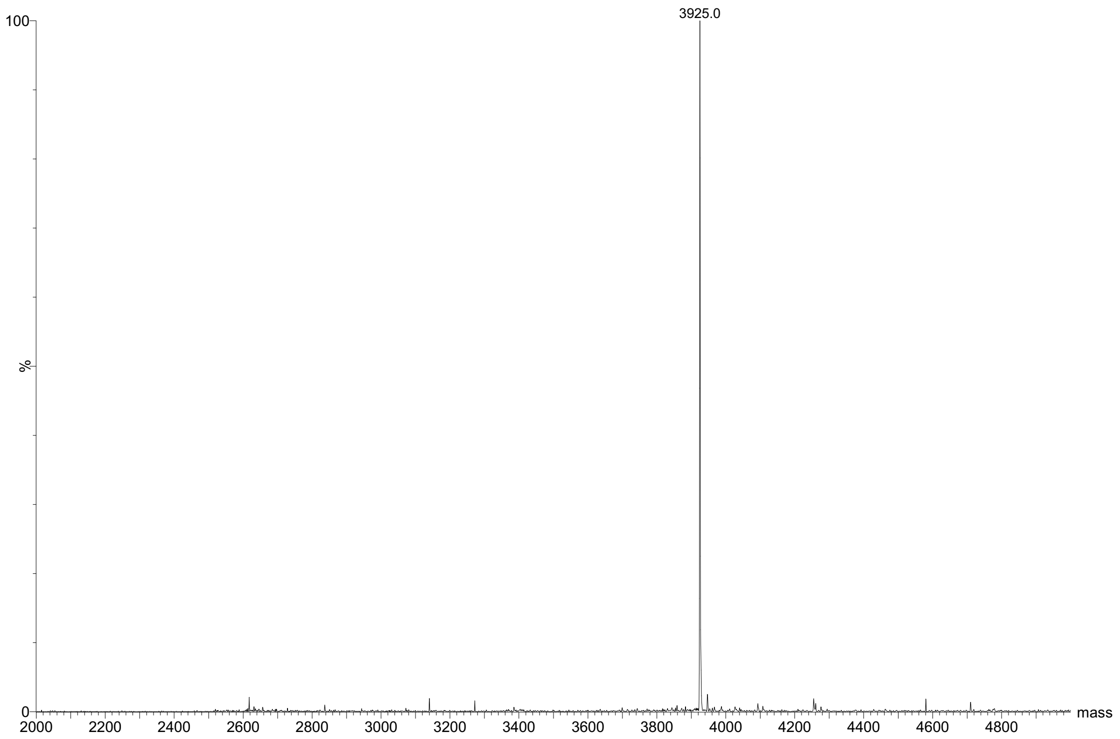

Supplementary Figure 29. Reverse-phase UPLC (UV absorbance at 260 nm vs time in min) and mass spectrum (ES^-) of ON3$^{DNA\text{-}Am\text{-}LNA}$ without purification. Required 3924.8 Da, found 3925.0 Da. CGACGCT*TGCAGC (DNA, phosphodiester backbone). y-axis = relative intensity (%), x-axis = mass in Da.

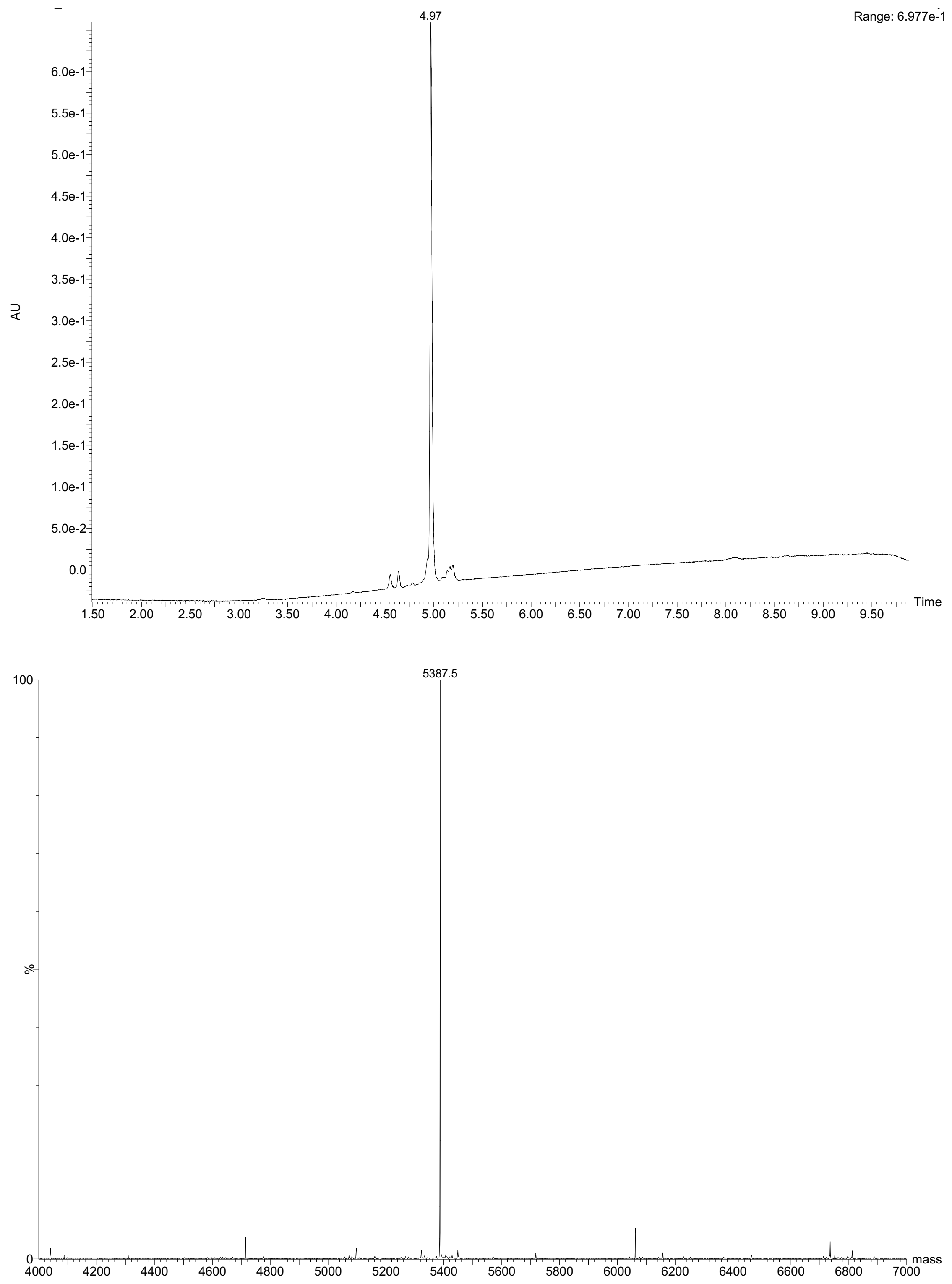

Supplementary Figure 30. Reverse-phase UPLC (UV absorbance at 260 nm vs time in min) and mass spectrum (ES^-) of ON13$^{DNA/1LAL/16PO}$ without purification. Required 5386.8 Da, found 5387.5 Da. CCTCTTACC*TCAGTTACA (DNA, phosphodiester backbone). y-axis = relative intensity (%), x-axis = mass in Da.

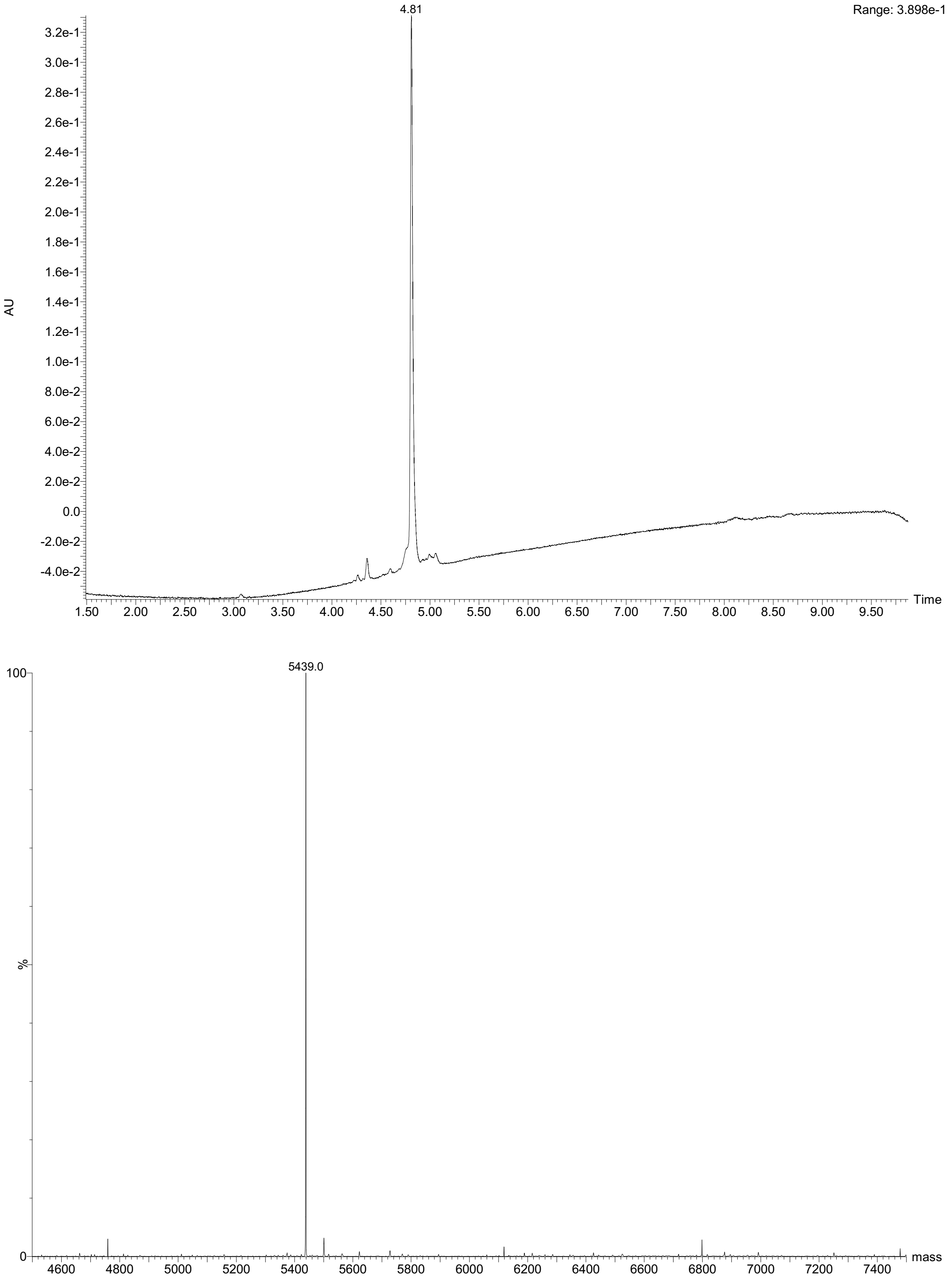

Supplementary Figure 31. Reverse-phase UPLC (UV absorbance at 260 nm vs time in min) and mass spectrum (ES^-) of ON14$^{DNA/4LA/13PO}$ without purification. Required 5438.7 Da, found 5439.0 Da. CC*TCT*TACC*TCAGT*TACA (DNA, phosphodiester backbone). y-axis = relative intensity (%), x-axis = mass in Da.

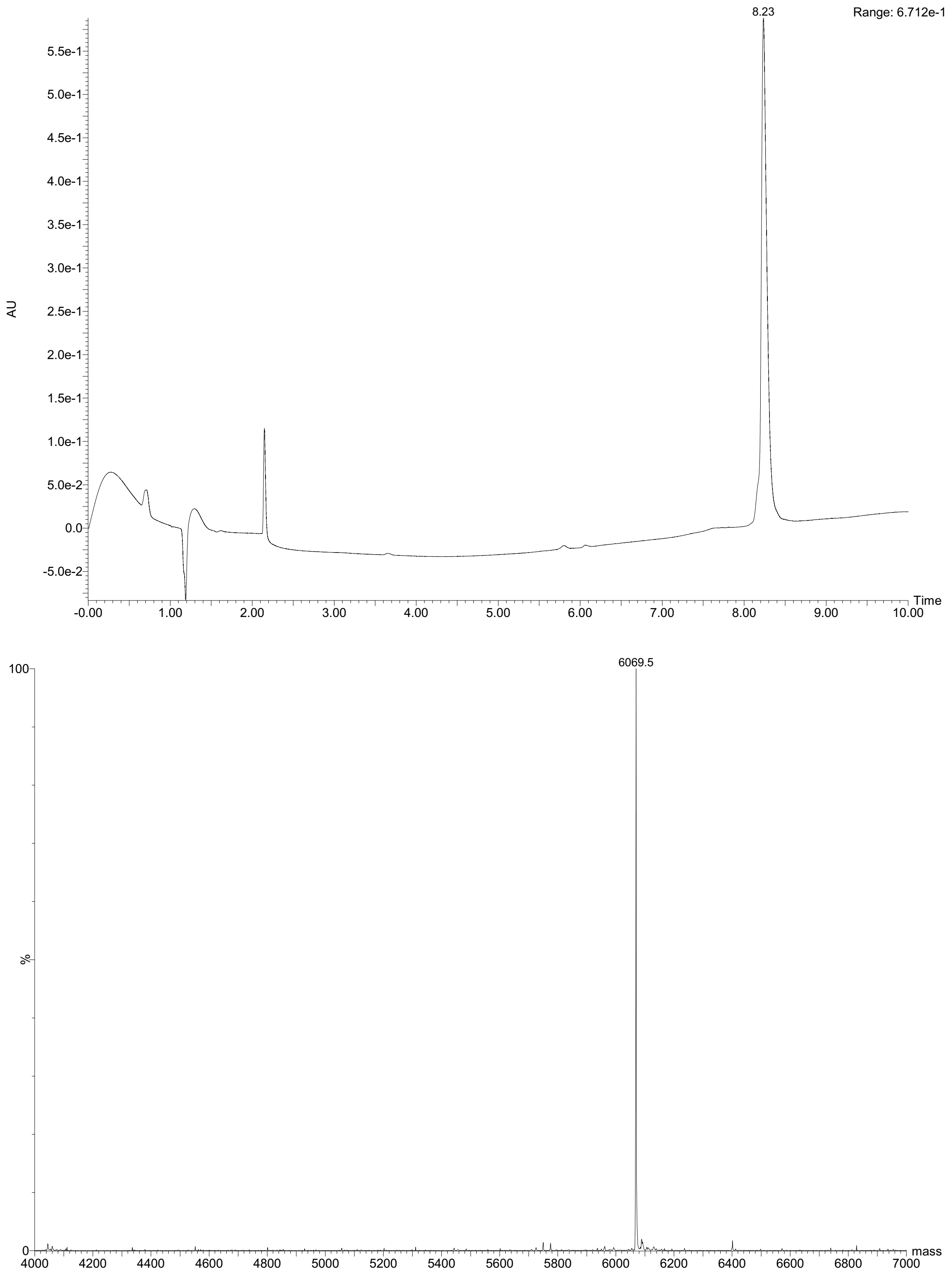

Supplementary Figure 32. Reverse-phase UPLC (UV absorbance at 260 nm vs time in min) and mass spectrum (ES^-) of 5´-DMT protected ON16$^{2´OMe/4LAL/13PO}$ after HPLC purification. Purification with DMT on yielded a higher purity oligonucleotide. Required 6069.3 Da, found 6069.5 Da**.** DMT-C^{Me}C*TCT*TAC^{Me}C*TCAGT*TACA (2´-OMe, phosphodiester backbone). y-axis = relative intensity (%), x-axis = mass in Da.

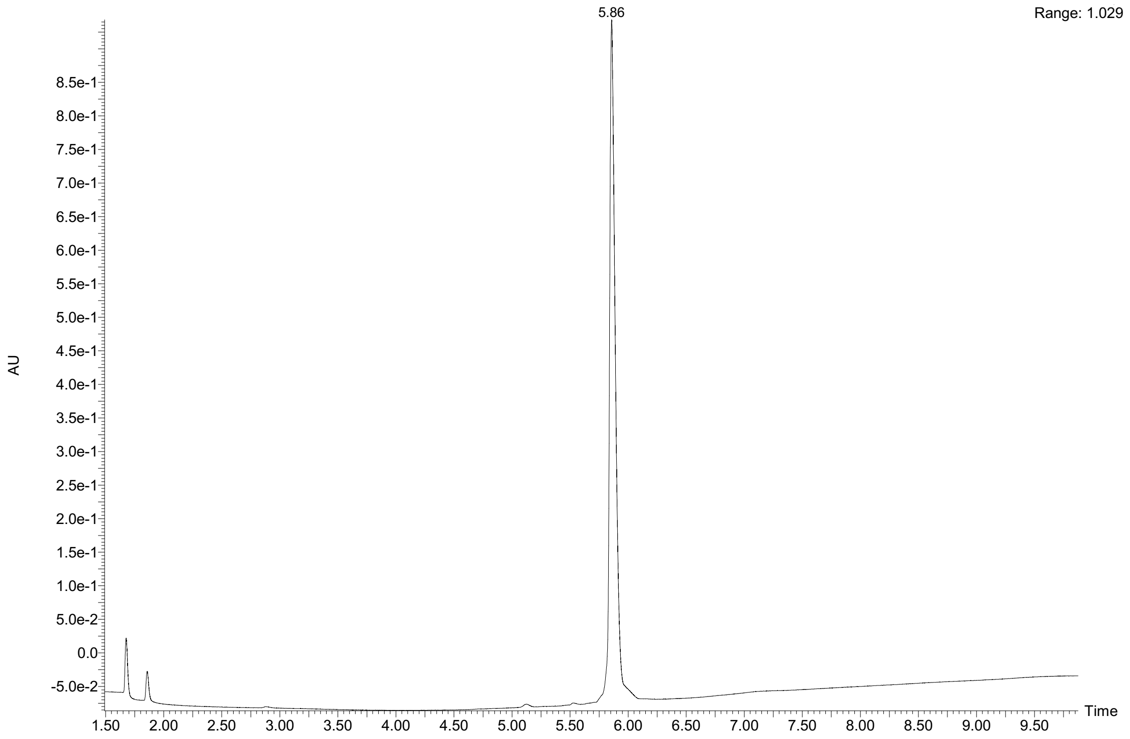

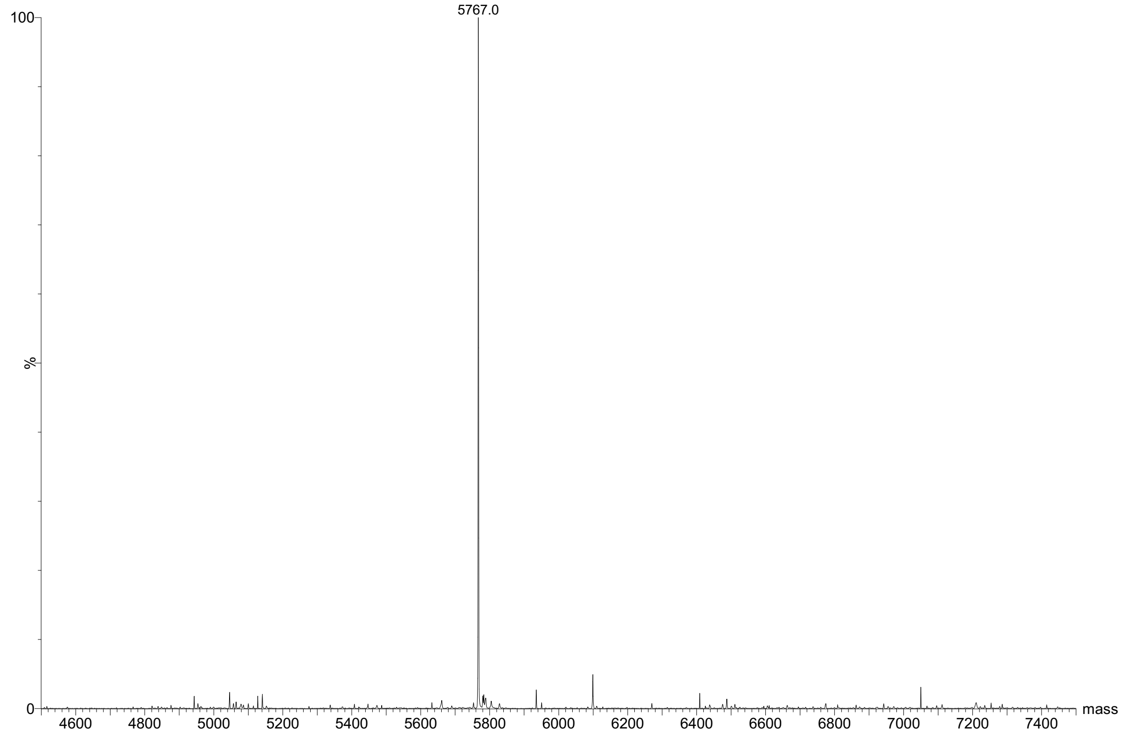

Supplementary Figure 33. Reverse-phase UPLC (UV absorbance at 260 nm vs time in min) and mass spectrum (ES$^-$) of ON16$^{2'OMe/4LAL/13PO}$ after HPLC purification. Required 5766.9 Da found, 5767.0 Da. C^{Me}C*TCT*TAC^{Me}C*TCAGT*TACA (2´-OMe, phosphodiester backbone). y-axis = relative intensity (%), x-axis = mass in Da.

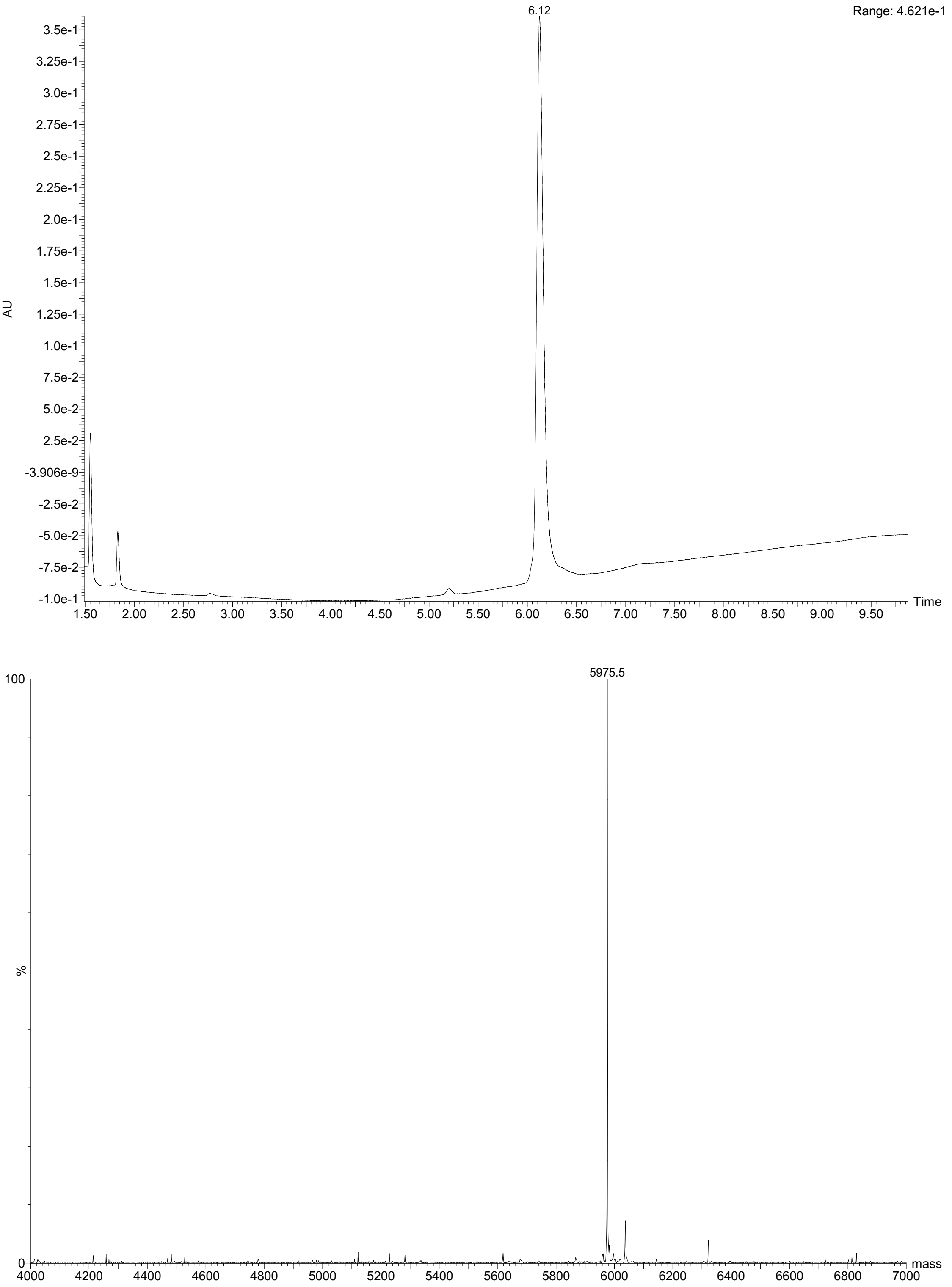

Supplementary Figure 34. Reverse-phase UPLC (UV absorbance at 260 nm vs time in min) and mass spectrum (ES$^-$) of ON18$^{2'OMe/4LAL/13PS}$ after HPLC purification. Required 5976.2 Da, found 5975.5 Da. C^{Me}C*TCT*TAC^{Me}C*TCAGT*TACA (2´-OMe, phosphorothioate backbone). y-axis = relative intensity (%), x-axis = mass in Da.

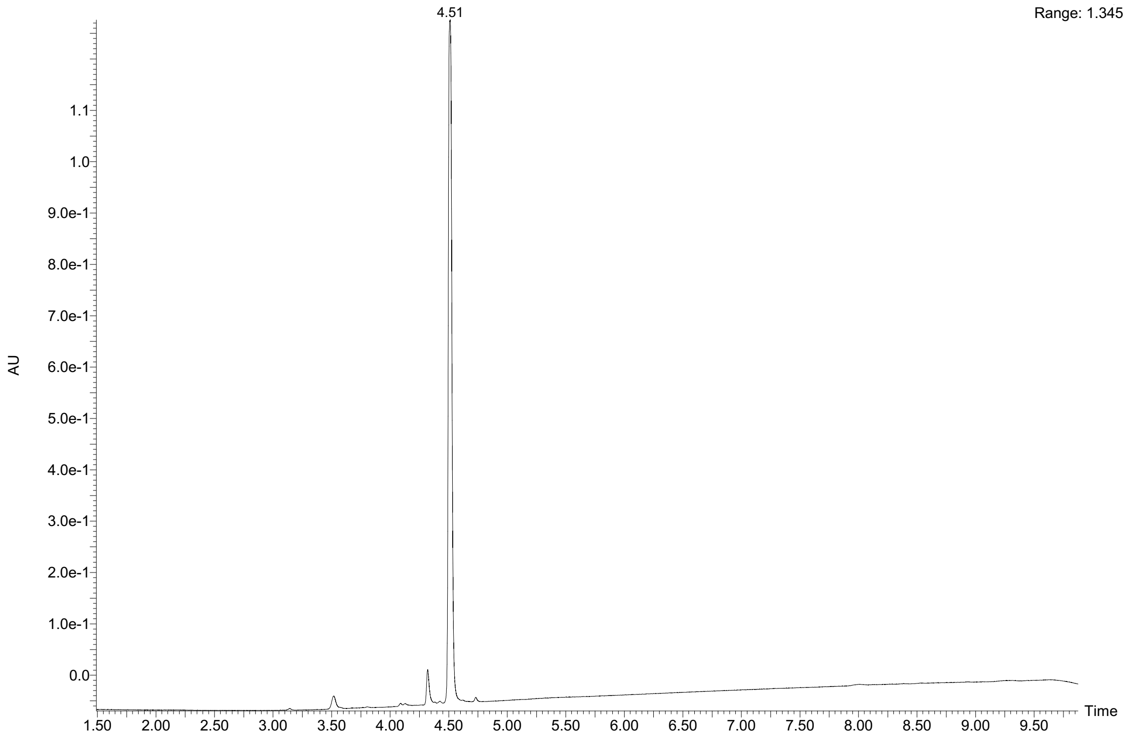

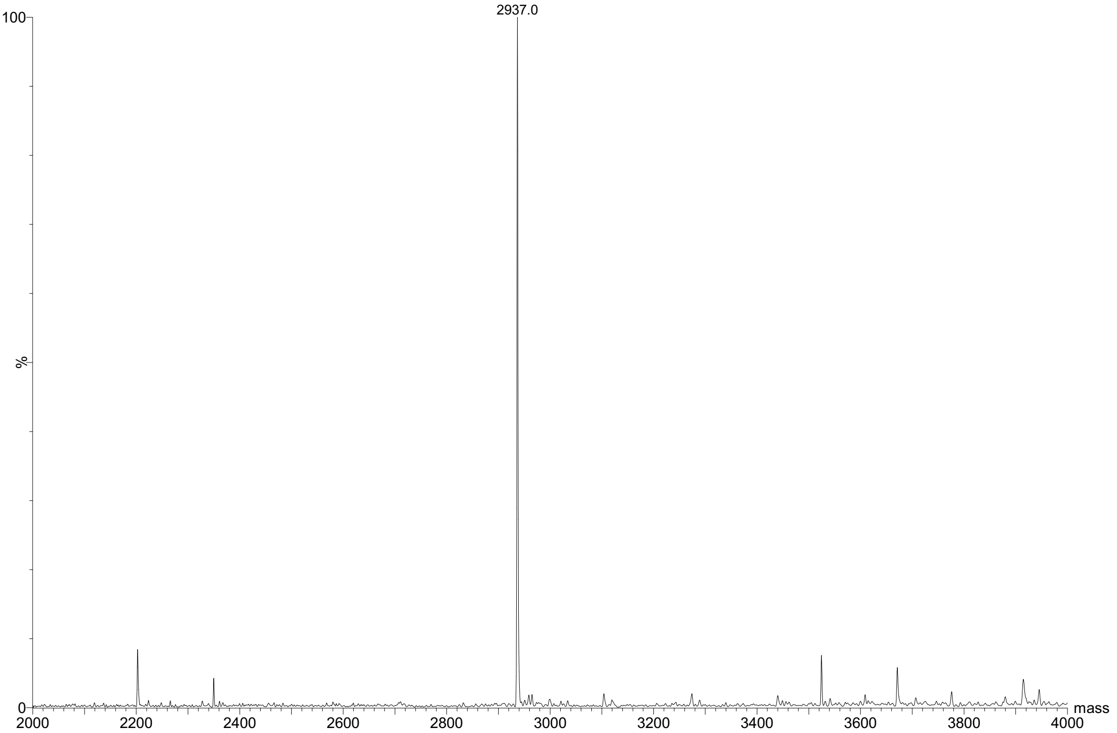

Supplementary Figure 35. Reverse-phase UPLC (UV absorbance at 260 nm vs time in min) and mass spectrum (ES$^-$) of ON28$^{xDNA\text{-}Am\text{-}DNA}$ following HPLC purification. Required 2936.1 Da, found 2937.0 Da. CTT*TTCTTTG (DNA, phosphodiester backbone). y-axis = relative intensity (%), x-axis = mass in Da.

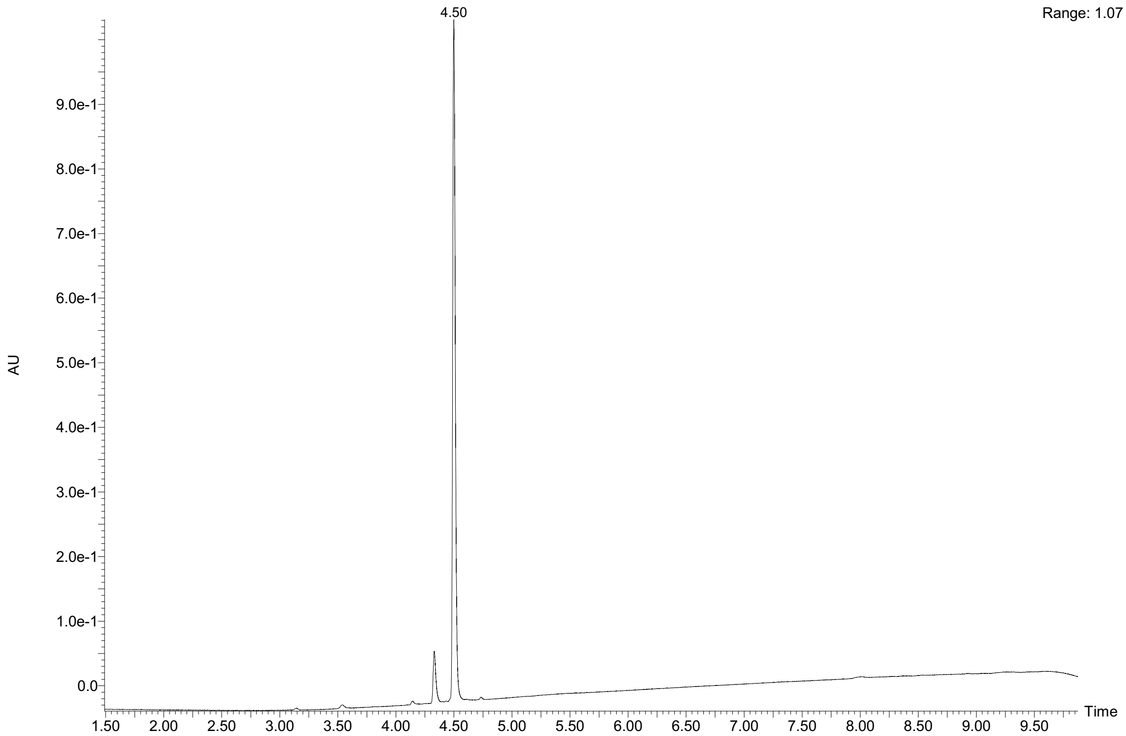

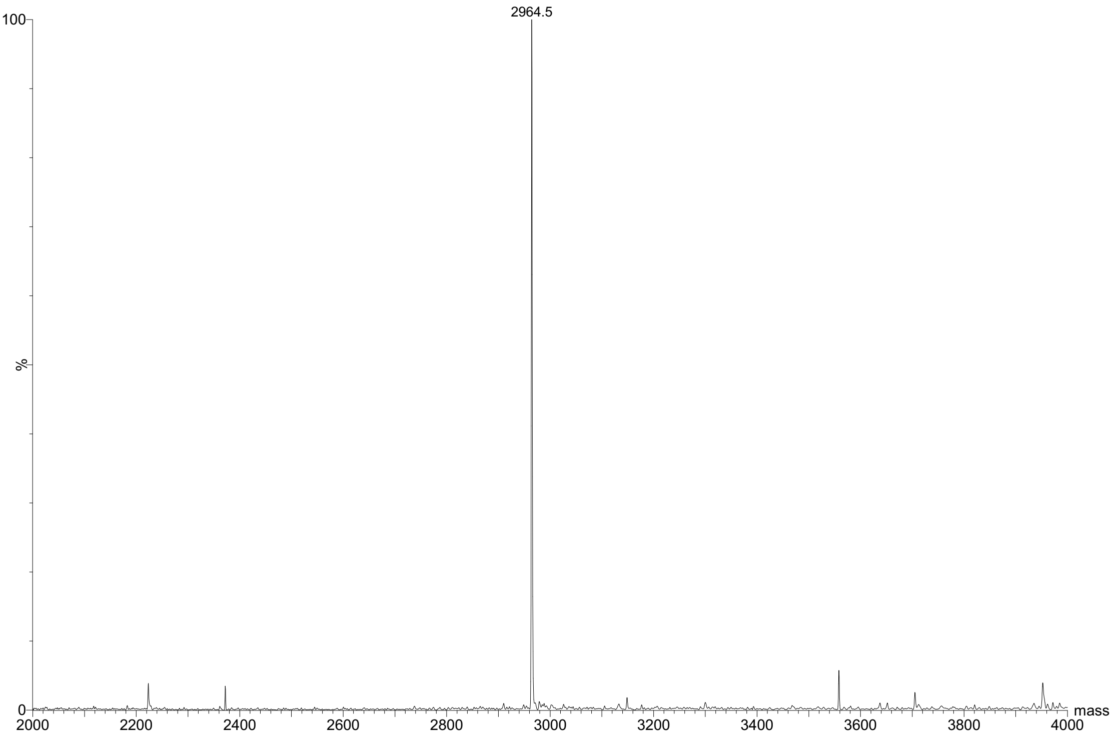

Supplementary Figure 36. Reverse-phase UPLC (UV absorbance at 260 nm vs time in min) and mass spectrum (ES$^-$) of ON29$^{xLNA\text{-}Am\text{-}DNA}$ after HPLC purification. Required 2964.2 Da, found 2964.5 Da. CT<u>T</u>*TTCTTTG (DNA, phosphodiester backbone). y-axis = relative intensity (%), x-axis = mass in Da.

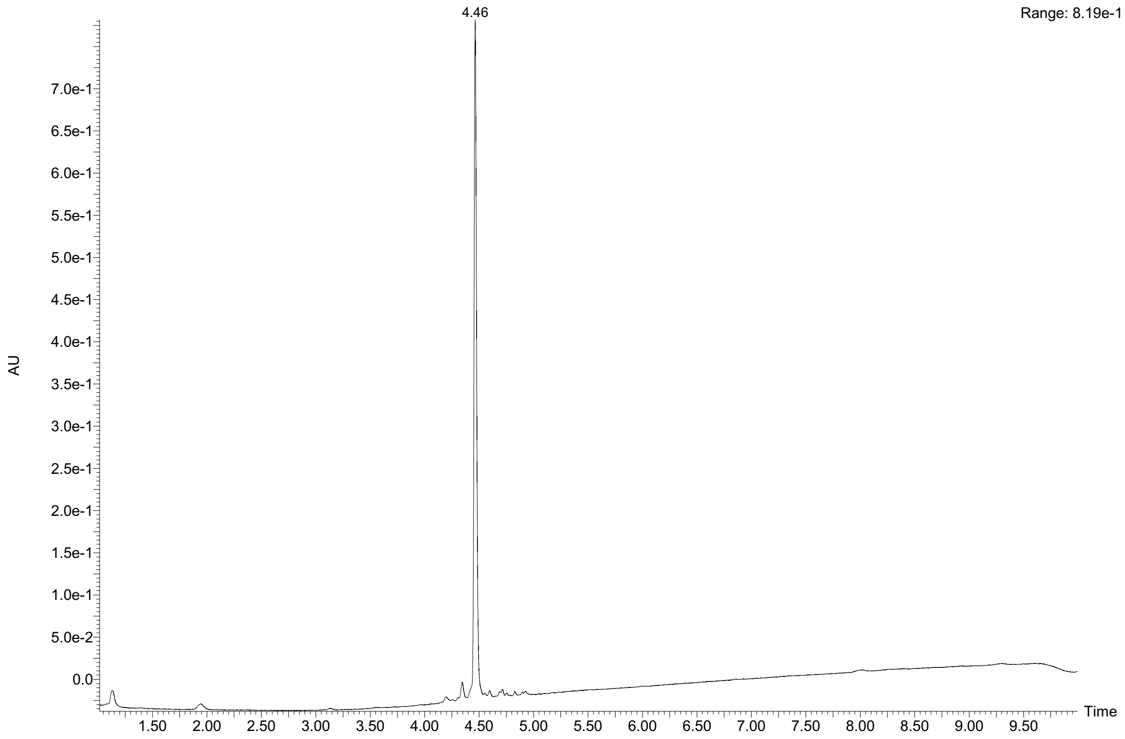

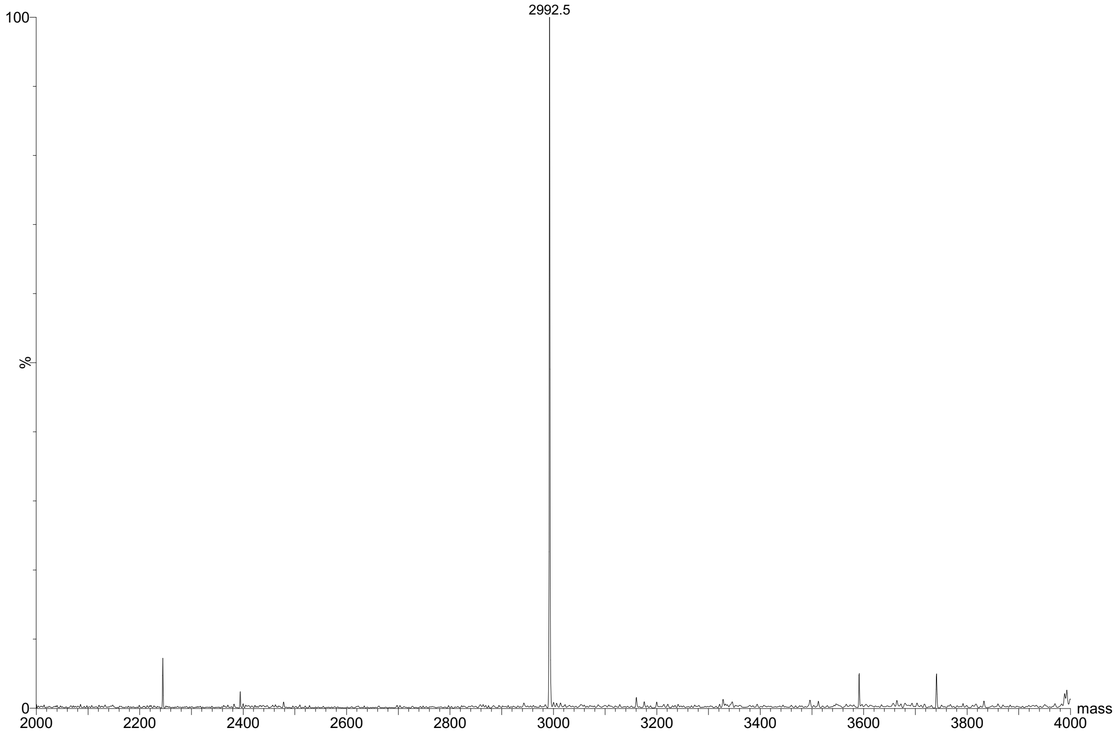

Supplementary Figure 37. Reverse-phase UPLC (UV absorbance at 260 nm vs time in min) and mass spectrum (ES$^-$) of ON30$^{xLNA\text{-}Am\text{-}LNA}$ without purification. Required 2992.3 Da, found 2992.5 Da. CT<u>T</u>*<u>T</u>TCTTTG (DNA, phosphodiester backbone). y-axis = relative intensity (%), x-axis = mass in Da.

Supplementary small molecule crystallography information

Single crystal X-ray diffraction data were collected using a (Rigaku) Oxford Diffraction SuperNova diffractometer and CrysAlisPro v41.117a. Structures were solved using Superflip through Crystals 03/15/13[5] before refinement with CRYSTALS v10.1.100 commit 8199 [6, 7] as per the CIF provided in the Supplementary Information. Crystallographic data have been deposited with the Cambridge Crystallographic Data Centre (CCDC 2105684-5) and can be obtained via www. ccdc.cam.ac.uk/data_request/cif.

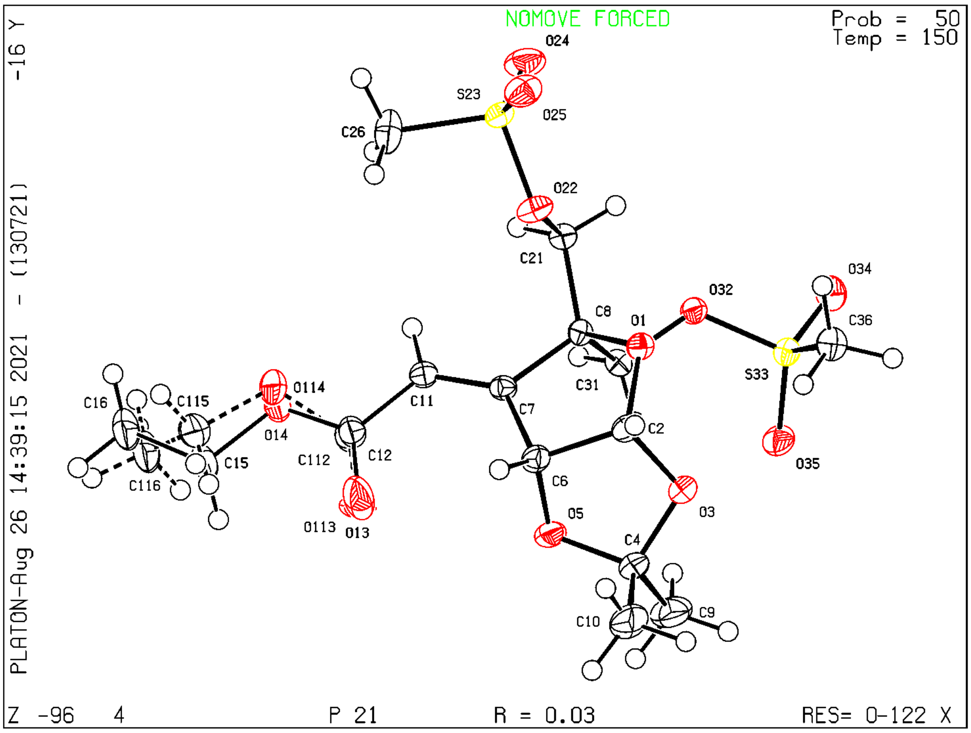

Supplementary Figure 38. Ellipsoid plot of compound **4**

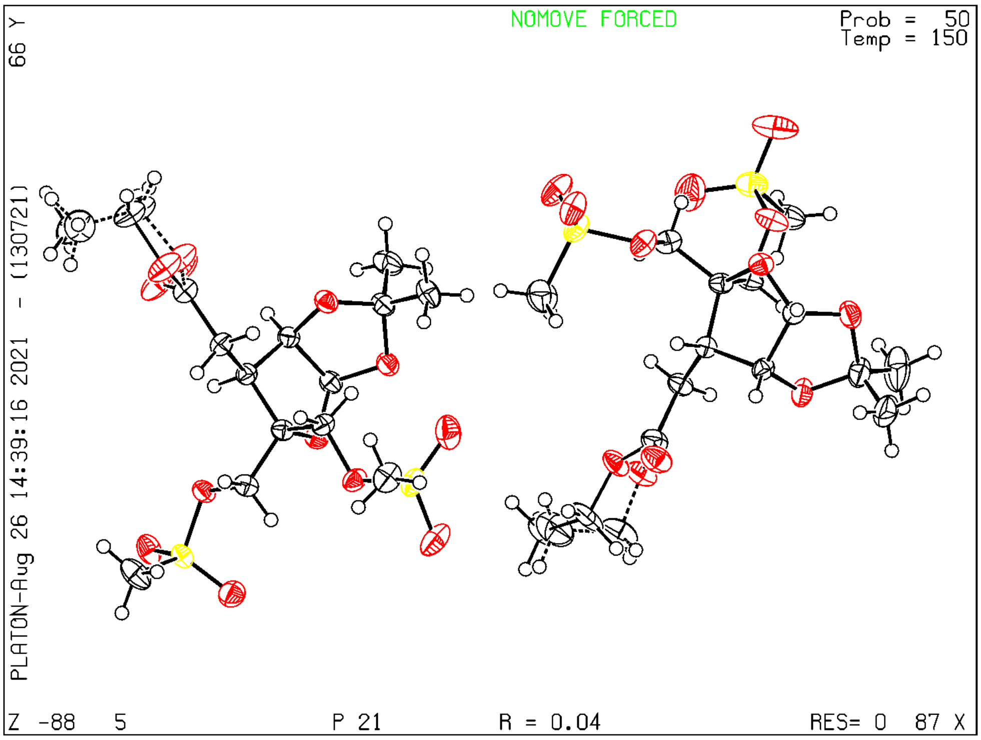

Supplementary Figure 39. Ellipsoid plot of compound **5**

Supplementary Table 7. Crystal data and structure refinement for **4**.

Empirical formula	C15 H24 O11 S2
Formula weight	444.48
Temperature	150 K
Wavelength	1.54184 Å
Crystal system	Monoclinic
Space group	P 2_1
Unit cell dimensions	a = 12.0289(2) Å, α= 90°.
	b = 5.42400(10) Å, β= 97.7502(15)°.
	c = 15.2984(2) Å, γ = 90°.
Volume	989.02(3) $Å^3$
Z	2
Density (calculated)	1.492 Mg/m^3
Absorption coefficient	2.962 mm^{-1}
F(000)	468
Crystal size	0.28 x 0.09 x 0.06 mm^3
Theta range for data collection	3.708 to 76.217°.
Index ranges	-14<=h<=15, -6<=k<=6, -17<=l<=19
Reflections collected	11945
Independent reflections	4099 [R(int) = 0.029]
Completeness to theta = 74.692°	99.80%
Absorption correction	Semi-empirical from equivalents
Max. and min. transmission	0.84 and 0.68
Refinement method	Full-matrix least-squares on F^2
Data / restraints / parameters	4098 / 29 / 289
Goodness-of-fit on F^2	1.0094
Final R indices [I>2sigma(I)]	R1 = 0.0261, wR2 = 0.0655
R indices (all data)	R1 = 0.0264, wR2 = 0.0661
Absolute structure parameter	-0.001(6)
Extinction coefficient	19(2)
Largest diff. peak and hole	0.11 and -0.09 e.$Å^{-3}$

Supplementary Table 8. Crystal data and structure refinement for **5**.

Empirical formula	C15 H26 O11 S2
Formula weight	446.5
Temperature	150 K
Wavelength	1.54184 Å
Crystal system	Monoclinic
Space group	P 2_1
Unit cell dimensions	a = 5.61600(10) Å, α= 90°.
	b = 18.1625(4) Å, β= 93.200(2)°.
	c = 19.9906(5) Å, γ = 90°.
Volume	2035.87(8) Å^3
Z	4
Density (calculated)	1.457 Mg/m^3
Absorption coefficient	2.878 mm^{-1}
F(000)	944
Crystal size	0.18 x 0.07 x 0.02 mm^3
Theta range for data collection	3.290 to 76.463°.
Index ranges	-6<=h<=6, -22<=k<=22, -24<=l<=25
Reflections collected	24089
Independent reflections	8349 [R(int) = 0.051]
Completeness to theta = 74.169°	99.60%
Absorption correction	Semi-empirical from equivalents
Max. and min. transmission	0.94 and 0.83
Refinement method	Full-matrix least-squares on F^2
Data / restraints / parameters	8348 / 143 / 553
Goodness-of-fit on F^2	0.9988
Final R indices [I>2sigma(I)]	R1 = 0.0399, wR2 = 0.0927
R indices (all data)	R1 = 0.0457, wR2 = 0.0978
Absolute structure parameter	-0.010(9)
Largest diff. peak and hole	0.47 and -0.25 e.Å^{-3}

Supplementary Methods

Small molecule synthesis

General

Unless otherwise stated, reactions were performed in oven-dried glassware under an inert argon using anhydrous solvents. Anhydrous solvents were collected from an mBraun SPS-800 bench top solvent purification system, having passed through anhydrous alumina columns. Solvents for phosphitylation reaction were degassed by bubbling with argon before used and pyridine and CH_2Cl_2 were further purified by distillation over KOH or CaH respectively. Anhydrous dichloroethane (Aldrich) was used as supplied without further purification. 3-O-Benzyl 4-C-(methanesulfonyloxymethyl)-5-O-methanesulfonyl-1,2-O-isopropylidene-a-D-ribofuranose was purchased from Carbosynth. All other chemicals were used as obtained from commercial sources without further purification.

Thin layer chromatography (TLC) was performed using Merck pre-coated 0.23 mm thick plates of Kieselgel 60 F254 and visualised using UV (λ = 254 nm) or by staining with $KMnO_4$, *p*-anisaldehyde, dinitrophenylhydrazine, iodine, or ninhydrin (depending on functionality). All retention factors (R_f) are given to 0.01 with the solvent system reported in parentheses. Column chromatography was carried out using Geduran Silica Gel 60 from Merck.

Melting points (mp) were measured using Gallenkamp melting point apparatus and are uncorrected.

^{1}H, ^{13}C and ^{31}P NMR spectra were recorded on a Bruker AVIIIHD 400, Bruker AVII 500 (with a ^{13}C cryoprobe), or Bruker NEO 600 (with broadband helium cryoprobe) spectrometer operating at 400, 500 or 600 MHz respectively using an internal deuterium lock at ambient probe temperatures. ^{1}H NMR chemical shifts (δ) are quoted to the nearest 0.01 ppm and are referenced relative to residual solvent peak. Coupling constants (J) are given to the nearest 0.1 Hz. The following abbreviations are used to indicate the multiplicity of signals: s = singlet, d = doublet, t = triplet, q = quartet, m = multiplet, and br = broad. Data is reported as follows: chemical shift (multiplicity, coupling constant(s), integration). ^{13}C NMR chemical shifts (δ) are quoted to the nearest 0.1 ppm and are reference relative to the deuterated solvent peak. NMR assignments are supported by DEPT, COSY, HMQC, and HMBC where necessary.

High resolution mass spectra (HRMS) were recorded on a Thermo Scientific Exactive Mass Spectrometer equipped with a Waters Equity autosampler and pump by the University of Oxford Chemistry Departmental Mass Spectrometry Service, and reported mass values are within ± 5 ppm mass units unless otherwise stated.

Unless otherwise stated, yields refer to analytically pure compounds.

4-*C*-(Methanesulfonyloxymethyl)-5-*O*-methanesulfonyl-1,2-*O*-isopropylidene-α-D-ribofuranose **2**

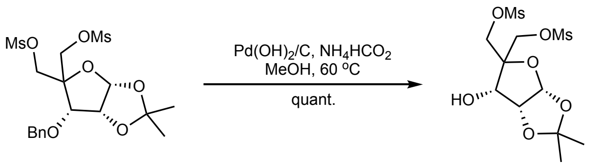

Commercially available 3-O-benzyl 4-C-(methanesulfonyloxymethyl)-5-O-methanesulfonyl-1,2-O-isopropylidene-a-D-ribofuranose **1** (9.8 g, 21.1 mmol) and ammonium formate (10 g, 159 mmol, 7.5 eq) were dissolved in MeOH (250 mL) and 20 wt% palladium hydroxide on carbon (1.48 g, 2.11 mmol, 10 mol%) was added. The flask was flushed with argon and the reaction was stirred at 60 °C overnight. A large volume of gas is generated within the first hour of the reaction presenting a risk of over-pressurisation. The reaction was filtered through celite to remove the catalyst and the solvent was removed under vacuum. The resulting solid was dissolved in EtOAc (100 mL), washed with a half-saturated aqueous solution of NaCl (2 x 100 mL), dried over $MgSO_4$, and evaporated to dryness to give **2** (7.9 g, 21.0 mmol) as a white solid in quantitative yield.

TLC (EtOAc:40-60 petroleum ether (PE), 7:3 v/v) R_f: 0.36;

^{1}H NMR (400 MHz, $CDCl_3$): δ 5.87 (d, J = 4.0 Hz, 1H), 4.75 (dd, J = 6.1, 4.0 Hz, 1H), 4.65 (d, J = 11.6 Hz, 1H), 4.50 (d, J = 11.6 Hz, 1H), 4.39 (dd, J = 7.7, 6.1 Hz, 1H), 4.33 (d, J = 10.9 Hz, 1H), 4.29 (d, J = 10.9 Hz, 1H), 3.12 (s, 3H), 3.08 (s, 3H), 2.88 (d, J = 7.7 Hz, 1H), 1.67 – 1.66 (s, 3H), 1.38 (s, 3H);

^{13}C NMR (101 MHz, $CDCl_3$): δ 114.1, 104.7, 84.3, 79.2, 72.7, 69.3, 68.6, 38.0, 37.7, 26.2, 26.0;

HRMS (m/z): $[M+Na]^+$ calcd. for $C_{11}H_{20}O_{10}NaS_2^+$, 399.0390; found, 399.0389.

4-*C*-(Methanesulfonyloxymethyl)-5-*O*-methanesulfonyl-1,2-*O*-isopropylidene-α-D-erythro-pentofuranos-3-ulose **3**

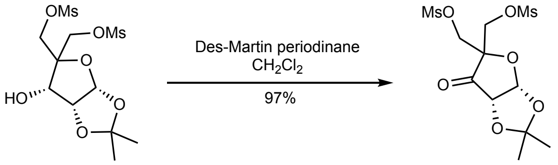

Alcohol **2** (17.4 g, 46.3 mmol) and Dess-Martin periodinane (29.7 g, 70.0 mmol, 1.5 eq) were dissolved in CH_2Cl_2 (300 mL) and the reaction was stirred at room temperature overnight. A solution of $Na_2S_2O_3$ in saturated aqueous $NaHCO_3$ (10% w/v, 200 mL) was added slowly to the reaction and stirring was continued until bubbling ceased. The biphasic mixture was filtered through celite to remove the white precipitate and the organic layer collected. The organic layer was washed twice more with saturated $NaHCO_3$ (200 mL), dried over Na_2SO_4, and evaporated to dryness to give **3** (16.7 g, 44.7 mmol) as colourless oil in 97% yield which crystallised on standing. This was used without further purification

TLC (EtOAc:40-60 PE, 6:4 v/v) R_f: 0.24;

^{1}H NMR (400 MHz, $CDCl_3$): δ 6.16 (d, J = 4.1 Hz, 1H), 4.52 (d, J = 4.1 Hz, 1H), 4.46 (d, J = 11.0 Hz, 1H), 4.45 (d, J = 11.7 Hz, 1H), 4.33 (d, J = 11.7 Hz, 1H), 4.32 (d, J = 11.0 Hz, 1H), 3.11 (s, 3H), 3.03 (s, 3H), 1.56 (s, 3H), 1.39 (s, 3H);

^{13}C NMR (101 MHz, $CDCl_3$): δ 205.0, 115.6, 103.0, 84.3, 76.7, 69.5, 68.9, 38.2, 37.9, 27.2, 26.8;

HRMS (m/z): $[M+Na]^+$ calcd. for $C_{11}H_{18}O_{10}NaS_2^+$, 397.0234; found, 397.0231.

Ethyl 2-((3aR,6aR)-2,2-dimethyl-5,5-bis(((methylsulfonyl)oxy)methyl)dihydrofuro[2,3-d][1,3]dioxol-6(5H)-ylidene)acetate **4**

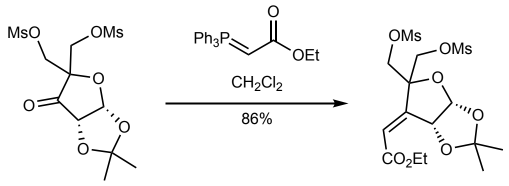

A solution of compound **3** (15.1 g, 40.4 mmol) and (carbethoxymethylene)triphenylphosphorane (16.9 g, 48.5 mmol, 1.2 eq) in CH_2Cl_2 (80 mL) was stirred at room temperature overnight. After removal of the solvent the resulting orange gum was triturated with EtOH, forming a white precipitate. The precipitate was collected by filtration, washed with cold EtOH, and dried under vacuum. The crude product was then purified by recrystallisation from hot EtOH to yield alkene **4** (15.4 g, 33.1 mmol) in 86% yield.

TLC (EtOAc:40-60 PE, 6:4 v/v) R_f: 0.64;

mp: 88-93 °C (crystallised from EtOH);

^1H NMR (400 MHz, $CDCl_3$): δ 6.07 (d, J = 1.3 Hz, 1H), 5.93 (d, J = 3.7 Hz, 1H), 5.79 (dd, J = 3.7, 1.3 Hz, 1H), 4.53 – 4.19 (m, 6H), 3.11 (s, 3H), 3.07 (s, 3H), 1.59 (s, 3H + H_2O), 1.41 (s, 3H), 1.32 (t, J = 7.1 Hz, 3H);

^{13}C NMR (101 MHz, $CDCl_3$): δ 164.0, 151.2, 121.4, 114.5, 105.6, 85.0, 78.7, 70.1, 69.5, 61.5, 38.2, 37.9, 27.2, 26.4, 14.2;

HRMS (m/z): $[M+Na]^+$ calcd. for $C_{15}H_{24}O_{11}NaS_2^+$, 467.0652; found, 467.0652.

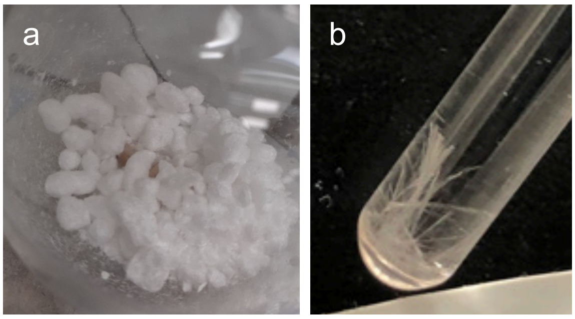

Supplementary Figure 40. crystalline product **4**. **a.** Crystalline product after work up. **b.** Crystals used for XRD studies

Ethyl 2-((3aR,6S,6aR)-2,2-dimethyl-5,5-bis(((methylsulfonyl)oxy)methyl)tetrahydrofuro[2,3-d][1,3]dioxol-6-yl)acetate **5**

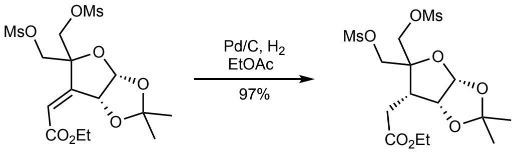

4 5

A solution of **4** (13.0 g, 29.3 mmol) in EtOAc (300 mL) was placed under an atmosphere of argon before 5% palladium on activated carbon (1.3 g, 1.5 mmol, 5 mol%) was added. The flask was evacuated under vacuum and refilled with H_2 gas three times to ensure a hydrogen atmosphere and vigorously stirred overnight. The reaction was monitored by NMR. Once complete, the mixture was filtered through a pad of celite and the solvent removed under reduced pressure to yield **5** (12.7 g, 28.4 mmol) as a colourless solid in 97% yield which was used without further purification. The use of Pd/C and hydrogen gas along with flammable solvents poses a significant fire risk.

TLC (EtOAc:40-60 PE, 6:4 v/v) R_f: 0.64;

mp: 88-95 °C (crystallised from a mixture of MeOH and EtOH);

^{1}H NMR (400 MHz, $CDCl_3$): δ 5.85 (d, J = 3.9 Hz, 1H), 4.87 (dd, J = 5.2, 3.9 Hz, 1H), 4.59 (d, J = 10.8 Hz, 1H), 4.31 (d, J = 10.7 Hz, 1H), 4.30 (d, J = 10.8 Hz, 1H), 4.23 (d, J = 10.7 Hz, 1H), 4.18 (apparent dq, J = 7.1, 2.2 Hz, 2H), 3.12 (s, 3H), 3.07 (s, 3H), 2.91 – 2.65 (m, 2H), 2.57 (dd, J = 16.9, 5.5 Hz, 1H), 1.60 (s, 3H), 1.30 (s, 3H), 1.28 (t, J = 7.2 Hz, 3H);

^{13}C NMR (101 MHz, $CDCl_3$): δ 171.3, 112.9, 105.3, 84.1, 81.7, 70.1, 68.2, 61.3, 42.7, 38.1, 37.7, 28.6, 26.3, 25.5, 14.2;

HRMS (m/z): $[M+Na]^+$ calcd. for $C_{15}H_{26}O_{11}NaS_2^+$, 469.0809; found, 469.0811.

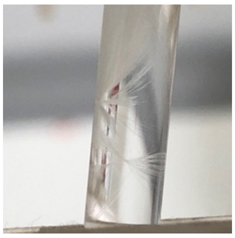

Supplementary Figure 41. Crystals of **5** used for XRD studies.

(3R,4S)-4-(2-Ethoxy-2-oxoethyl)-5,5-bis(((methylsulfonyl)oxy)methyl)tetrahydrofuran-2,3-diyl diacetate **6**

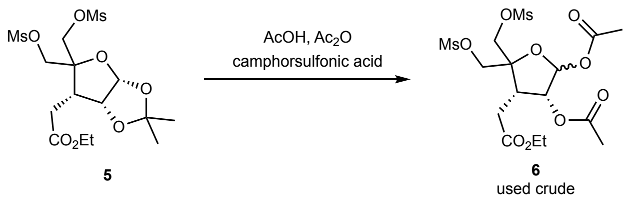

To a solution of compound **5** (4.9 g, 11.0 mmol) in acetic acid (50 mL) and acetic anhydride (38 mL) was added camphorsulfonic acid (CSA) (120 mg, 0.52 mmol) and the solution was stirred at 80 °C for 90 min. A second portion of CSA (120 mg, 0.52 mmol) was added and stirring continued at 80 °C for 90 min. This was repeated twice more, and the reaction left to stir overnight at 80 °C. The volatiles were removed under reduced pressure and the resulting brown gum was co-evaporated with toluene (3 x 50 mL), dissolved in EtOAc (150 mL), washed with a saturated aqueous solution of $NaHCO_3$ (5 x 100 mL), washed with brine (1 x 100 mL), dried over Na_2SO_4, and evaporated to dryness to yield crude compound **6** (5.3 g, assume quantitative) which was used without purification in the next step. We found the compound was not stable to column chromatography.

TLC (EtOAc:40-60 PE, 6:4 v/v) R_f: 0.38;

^{1}H NMR (400 MHz, $CDCl_3$): δ 6.10 (s, 1H), 5.32 (d, J = 5.0 Hz, 1H), 4.40 (d, J = 3.3 Hz, 2H), 4.28 (s, 2H), 4.20 – 4.11 (m, 2H), 3.08 (s, 3H), 3.07 (s, 3H), 3.03 (dd, J = 7.7, 4.9 Hz, 1H), 2.62 (t, J = 7.5 Hz, 2H), 2.14 (s, 3H), 2.11 (s, 3H), 1.26 (t, J = 7.2 Hz, 3H);

^{13}C NMR (101 MHz, $CDCl_3$): δ 170.9, 169.5, 169.3, 97.8, 84.6, 77.8, 71.4, 66.9, 61.6, 40.4, 38.0, 37.6, 28.6, 21.1, 20.8, 14.2;

HRMS (m/z): $[M+Na]^+$ calcd. for $C_{16}H_{26}O_{13}NaS_2^+$, 513.0707; found, 513.0706.

Thymine LNA acid precursor **7a**

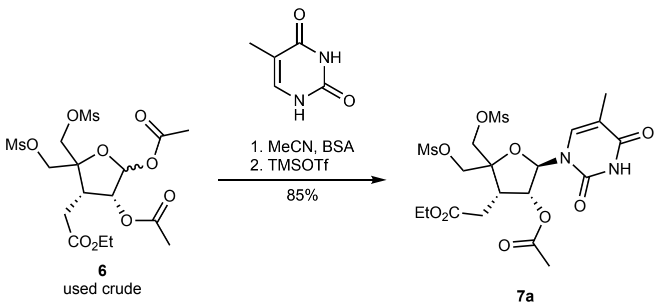

Crude compound **6** (2.5 g, 5.1 mmol) and thymine (0.8 g, 6.4 mmol, 1.25 eq) were co-evaporated with anhydrous MeCN (3 x 15 mL). The mixture was then dissolved in anhydrous MeCN (12.5 mL) and bis(trimethylsilyl)acetamide (BSA) (3.5 mL, 14.2 mmol, 2.8 eq) was added. The solution was heated to reflux for 1 h. The reaction was cooled to room temperature and TMSOTf (1.25 mL, 6.8 mmol, 1.4 eq) was added. The reaction was then heated to reflux overnight resulting in a dark red solution. The reaction was cooled to room temperature, diluted with CH_2Cl_2 (12.5 mL), and a half saturated aqueous solution of $NaHCO_3$ (25 mL) was added with stirring (bubbles are generated). The organic layer became a pale-yellow colour and was subsequently washed with saturated aqueous $NaHCO_3$ (25 mL) and brine (25 mL). The organic phase was dried over Na_2SO_4 and evaporated to dryness. The crude brown foam was purified by column chromatography (0-7% MeOH in CH_2Cl_2) to give compound **7a** (2.42 g, 4.4 mmol) as a beige foam in 85% yield.

TLC (CH_2Cl_2:MeOH, 17:1 v/v) R_f: 0.55;

^{1}H NMR (400 MHz, $CDCl_3$): δ 9.29 (s, 1H), 7.03 (d, J = 1.2, 1H), 5.58 (dd, J = 7.6, 1.7, 1H), 5.49 (d, J = 1.7, 1H), 4.47 (s, 2H), 4.38 (d, J = 11.0, 1H), 4.32 (d, J = 11.0, 1H), 4.15 (q, J = 7.1, 2H), 3.55 (ddd, J = 9.6, 7.6, 6.2, 1H), 3.10 (s, 3H), 3.09 (s, 3H), 2.65 (dd, J = 16.8, 9.6, 1H), 2.57 (dd, J = 16.8, 6.2, 1H), 2.14 (s, 3H), 1.91 (d, J = 1.2, 3H), 1.25 (t, J = 7.1, 3H);

^{13}C NMR (101 MHz, $CDCl_3$): δ 170.8, 170.3, 163.9, 150.3, 138.5, 111.6, 95.8, 85.5, 78.5, 70.1, 67.4, 61.5, 41.4, 37.9, 37.6, 29.1, 20.8, 14.3, 12.4;

HRMS (m/z): $[M+Na]^+$ calcd. for $C_{19}H_{28}N_2O_{13}NaS_2^+$, 579.0925; found 579.0924.

N[4]-Benzoylcytosine LNA acid precursor **7b**

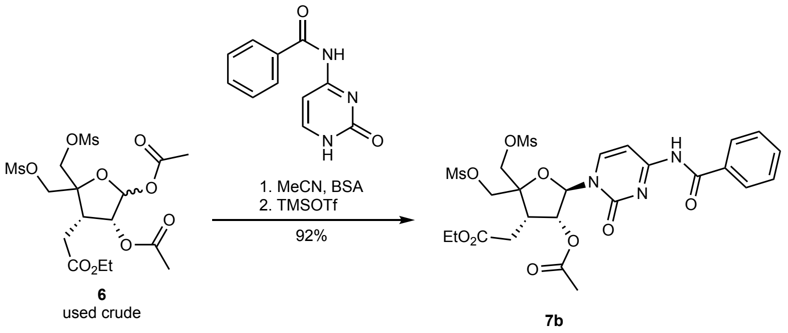

Compound **6** (1.04 g, 2.1 mmol) and *N*[4]-benzoylcytosine (0.912 g, 4.0 mmol, 2.0 eq) were co-evaporated with anhydrous MeCN (3 x 15 mL). The mixture was then dissolved in anhydrous MeCN (12.5 mL) and BSA (1.0 mL, 4.1 mmol, 1.9 eq) was added. The suspension was heated to reflux for 1 h. The reaction was cooled to room temperature and TMSOTf (0.45 mL, 2.5 mmol, 1.2 eq) was added. The reaction was then heated to reflux overnight resulting in a dark red solution. The reaction was cooled to room temperature, diluted with CH_2Cl_2 (12.5 mL), and a half saturated aqueous solution of $NaHCO_3$ was added with stirring. The organic layer was subsequently washed with saturated aqueous $NaHCO_3$ (25 mL) and brine (25 mL). The organic phase was dried over Na_2SO_4 and evaporated to dryness. The crude brown foam was purified by column chromatography (50-100% EtOAc in 40-60 PE) to give **7b** (1.25 g, 1.9 mmol) as a pale-yellow foam in 92% yield.

TLC (CH_2Cl_2:MeOH, 17:1 v/v) R_f: 0.53;

^{1}H NMR (400 MHz, $CDCl_3$): δ 8.97 (s, 1H), 7.93 (d, J = 7.7, 2H), 7.73 (d, J = 7.5, 1H), 7.65 – 7.50 (m, 4H), 5.67 (dd, J = 7.4, 1.5, 1H), 5.58 (d, J = 1.5, 1H), 4.62 – 4.49 (m, 2H), 4.45 – 4.36 (m, 2H), 4.16 (q, J = 7.1, 2H), 3.66 (apparent dt, J = 9.1, 7.0, 1H), 3.11 (s, 3H), 3.09 (s, 3H), 2.69 (dd, J = 16.8, 9.1, 1H), 2.59 (dd, J = 16.7, 6.8, 1H), 2.17 (s, 3H), 1.26 (t, J = 7.1, 3H);

^{13}C NMR (151 MHz, $CDCl_3$): δ 170.7, 170.4, 166.3 (broad due to rotamers), 162.6, 153.3 (broad due to rotamers), 148.5, 133.8, 132.4, 129.3, 128.2, 97.8, 96.9 (broad due to rotamers), 86.7, 78.7, 70.1, 67.6, 61.5, 41.4, 38.0, 37.7, 29.3, 20.8, 14.3;

HRMS (m/z): $[M+H]^+$ calcd. for $C_{25}H_{32}O_{13}N_3S_2^+$, 646.1371; found, 646.1367.

N^4-Benzoyl 5-methylcytosine LNA precursor **7c**

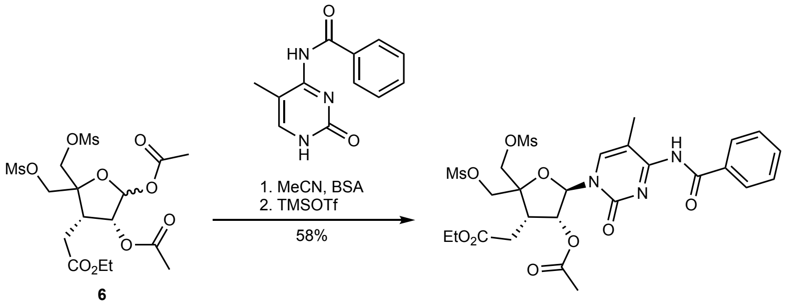

6
used crude

7c

A suspension of N^4-benzoyl methylcytosine (808 mg, 3.5 mmol, 1.5 eq), compound **6** (1.13 g, 2.3 mmol) and BSA (1.5 mL, 6.1 mmol, 2.7 eq) in anhydrous MeCN (13.5 mL) was heated to reflux for 1 h. The solution was cooled to room temperature, TMSOTf (0.5 mL, 2.8 mmol, 1.2 eq) was added dropwise with stirring and the reaction was then heated to reflux overnight. After cooling to room temperature, the reaction was diluted with EtOAc (30 mL) and a saturated aqueous solution of $NaHCO_3$ (30 mL) was added slowly (generates bubbles). Stirring the biphasic mixture generated a precipitate, which was filtered removing the precipitate prior to workup. The organic layer was collected, washed with saturated aqueous $NaHCO_3$ (2 x 30 mL) followed by brine (30 mL), dried over $MgSO_4$, and evaporated to dryness to give an orange foam. This was purified by column chromatography (0-10% MeOH in CH_2Cl_2) to give **7c** (885 mg, 1.3 mmol) as a beige foam in 58% yield.

TLC (CH_2Cl_2:MeOH, 17:1 v/v) R_f: 0.5;

^{1}H NMR (400 MHz, $CDCl_3$): δ 13.24 (s, 1H), 8.34 – 8.29 (m, 2H), 7.54 (tt, J = 7.4, 1.4, 1H), 7.45 (t, J = 7.4, 2H), 7.21 (d, J = 1.2, 1H), 5.62 (dd, J = 7.5, 1.7, 1H), 5.54 (d, J = 1.7, 1H), 4.49 (d, J = 10.8, 2H), 4.46 (d, J = 10.8 , 1H), 4.41 (d, J = 11.0, 1H), 4.33 (d, J = 11.0, 1H), 4.17 (q, J = 7.1, 2H), 3.58 (apparent dt, J = 9.2, 6.9, 1H), 3.11 (s, 3H), 3.09 (s, 3H), 2.67 (dd, J = 16.8, 9.2, 1H), 2.58 (dd, J = 16.8, 6.6, 1H), 2.17 (s, 3H), 2.12 (d, J = 1.2, 3H), 1.27 (t, J = 7.1, 3H);

^{13}C NMR (151 MHz, $CDCl_3$): δ 180.0, 170.7, 170.2, 159.7, 147.8, 139.1, 137.1, 132.8, 130.2, 128.3, 112.7, 96.1, 85.7, 78.5, 69.8, 67.3, 61.5, 41.3, 38.0, 37.7, 29.1, 20.8, 14.3, 13.5;

HRMS (m/z): $[M+H]^+$ calcd. for $C_{26}H_{34}O_{13}N_3S_2^+$, 660.1528; found, 660.1522.

N^6-Benzoyladenine LNA acid precursor **7d**

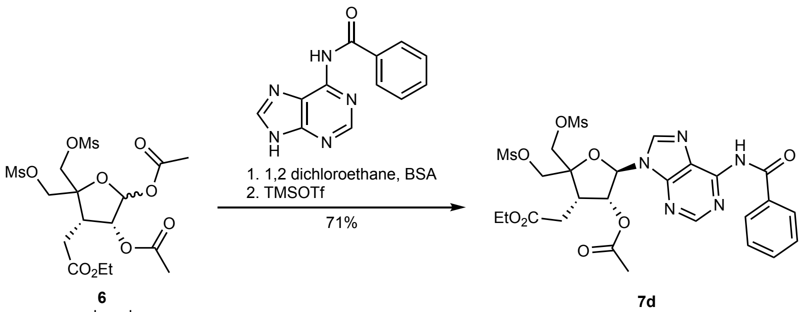

6
used crude

7d

N^6-Benzoyladenine (1.15 g, 4.8 mmol) and compound **6** (2.63 g, 5.3 mmol, 1.1 eq) were suspended in anhydrous 1,2-dichlorethane (22 mL) and BSA (3.13 mL, 12.8 mmol, 2.7 eq) was added. The solution was heated to reflux for 1 h. The reaction was cooled to room temperature and TMSOTf (2.0 mL, 11 mmol, 2.3 eq) was added. The reaction was then heated to reflux overnight resulting in a dark red solution. The reaction

was cooled to room temperature, diluted with CH_2Cl_2 (12.5 mL), and added to a saturated aqueous solution of $NaHCO_3$ (22 mL) slowly with stirring (bubbles are generated). The organic layer was subsequently washed with saturated aqueous $NaHCO_3$ (25 mL) and brine (25 mL), dried over Na_2SO_4, and evaporated to dryness. The crude brown foam was purified by silica column chromatography (0-100% EtOAc in 40-60 PE) to give compound **7d** (2.29 g, 3.4 mmol) as a beige foam in 71% yield.

TLC (EtOAc) R_f: 0.35;

^{1}H NMR (400 MHz, $CDCl_3$): δ 9.17 (s, 1H), 8.80 (s, 1H), 8.12 (s, 1H), 8.04 – 7.93 (m, 2H), 7.64 – 7.55 (m, 1H), 7.51 (t, J = 7.51, 2H), 6.13 (d, J = 1.1, 1H), 5.88 (dd, J = 6.7, 1.1, 1H), 4.55 (d, J = 1.9, 2H), 4.48 (d, J = 10.9, 1H), 4.40 (d, J = 10.9, 1H), 4.16 (q, J = 7.1, 2H), 4.12 – 4.02 (m, 1H), 3.11 (s, 3H), 2.95 (s, 3H), 2.72 (apparent dd, J = 7.8, 4.9, 2H), 2.19 (s, 3H), 1.25 (t, J = 7.1, 3H);

^{13}C NMR (101 MHz, $CDCl_3$): δ 170.7, 170.1, 164.8, 152.8, 151.1, 150.0, 142.6, 133.7, 133.0, 129.0, 128.0, 123.7, 90.8, 86.0, 79.0, 69.6, 67.0, 61.5, 41.6, 37.9, 37.6, 28.9, 20.8, 14.2;

HRMS (m/z): $[M+H]^+$ calcd. for $C_{26}H_{32}O_{12}N_5S_2^+$, 670.1483; found, 670.1482.

N^2-Isobutyrylguanine LNA acid precursor **7e**

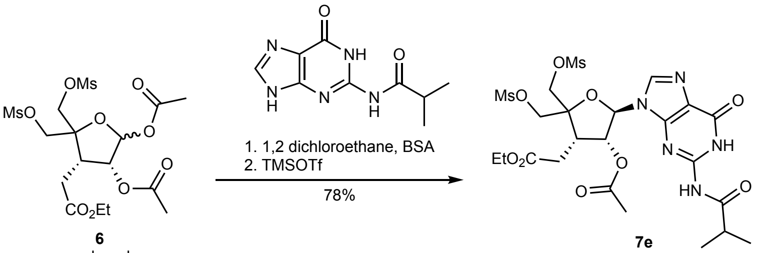

6
used crude

7e

Compound **6** (2.6 g, 5.3 mmol) and *N*^{2}-isobutyrylguanine (1.34 g, 6.1 mmol, 1.1 eq) were suspended in anhydrous 1,2 dichloroethane (22 mL) and BSA (3.1 mL, 12.5 mmol, 2.4 eq) was added. The suspension was heated to reflux for 1.5 h. The reaction was cooled to room temperature and TMSOTf (2.0 mL, 11 mmol, 2.1 eq) was added. The reaction was then heated to reflux for 2 h. The reaction was cooled to room temperature and added to a stirring solution of saturated aqueous $NaHCO_3$ (22 mL). The organic layer was subsequently washed with saturated aqueous $NaHCO_3$ (25 mL) and brine (25 mL). The organic phase was dried over Na_2SO_4 and evaporated to dryness. Purification by column chromatography (0-10% MeOH in EtOAc) gave **7e** (2.64, 4.1 mmol) as a pale-yellow foam in 78% yield.

TLC (EtOAc:MeOH, 9:1 v/v) R_f: 0.45;

^{1}H NMR (400 MHz, $CDCl_3$): δ 12.16 (s, 1H), 9.44 (s, 1H), 7.75 (s, 1H), 5.96 (d, J = 1.1, 1H), 5.72 (dd, J = 6.5, 1.1, 1H), 4.72 (d, J = 10.5, 1H), 4.49 (d, J = 11.1, 1H), 4.42 (d, J = 10.5, 1H), 4.32 (d, J = 11.1, 1H), 4.25 (dt, J = 8.9, 6.6, 1H), 4.13 (apparent qd, J = 7.2, 1.1, 2H), 3.12 (s, 3H), 3.06 (s, 3H), 2.77 – 2.57 (m, 3H), 2.16 (s, 3H), 1.30 – 1.16 (m, 9H);

^{13}C (101 MHz, $CDCl_3$): δ 179.4, 171.1, 170.0, 155.5, 148.1, 147.4, 139.2, 122.0, 91.0, 85.4, 78.5, 69.6, 67.6, 61.5, 41.4, 38.0, 37.7, 36.4, 28.6, 20.7, 19.0, 18.9, 14.2;

HRMS (m/z): $[M+H]^+$ calcd. for $C_{23}H_{34}O_{13}N_5S_2^+$, 652.1589; found, 652.1586.

Thymine LNA acid **8a**

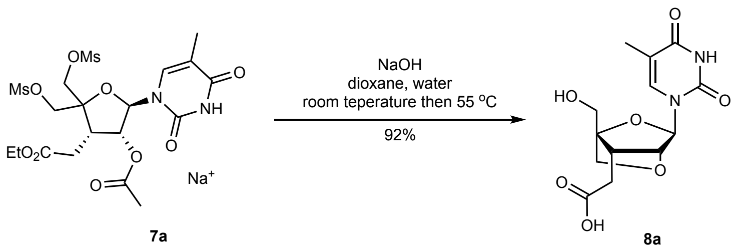

Compound **7a** (1.0 g, 1.8 mmol) was dissolved in 1,4-dioxane (4.5 mL) and water (4.5 mL) and 2 M NaOH in water (9 mL, 18 mmol, 10 eq) was added. The reaction was stirred at room temperature for 2 h until the locking step and ester hydrolysis was complete (reaction progress monitored using LCMS). The reaction was then heated to 55 °C for 1 h. The reaction was evaporated to dryness and partitioned between CH_2Cl_2 (40 mL) and water (30 mL). The aqueous layer was washed with CH_2Cl_2 (3 x 10 mL). The aqueous phase was acidified using 1 M HCl and washed with CH_2Cl_2 (3 x 20 mL). The product was then extracted from the aqueous layer using 25% iPrOH in CH_2Cl_2 (4 x 10 mL, until no product remained in the aqueous layer as determined by TLC), dried over Na_2SO_4, and evaporated to dryness to give **8a** (516 mg, 1.7 mmol) as a white solid in 92% yield which was used without further purification.

TLC (CH_2Cl_2:MeOH, 3:2 v/v + 2% Et_3N) R_f: 0.26;

^{1}H NMR (400 MHz, d_6-DMSO): δ 11.39 (s, 1H), 7.62 (d, J = 1.3, 1H), 5.44 (s, 1H), 4.35 (s, 1H), 3.81 (d, J = 13.0, 1H), 3.77 (d, J = 13.0, 1H), 3.73 (d, J = 8.5, 1H), 3.60 (d, J = 8.4, 1H), 3.31 (s, 1H), 2.41 (dd, J = 15.5, 2.7, 1H), 2.33 – 2.07 (m, 2H), 1.79 (d, J = 1.2, 3H);

^{13}C NMR (101 MHz, d_6-DMSO): δ 173.4, 164.3, 150.5, 135.4, 108.8, 91.1, 86.8, 80.2, 71.5, 57.1, 39.7, 29.0, 12.9;

HRMS (m/z): $[M-H]^-$ calcd. for $C_{13}H_{15}O_7N_2^-$, 311.0885; found, 311.0882.

N^4-Benzoylcytosine LNA acid **8b**

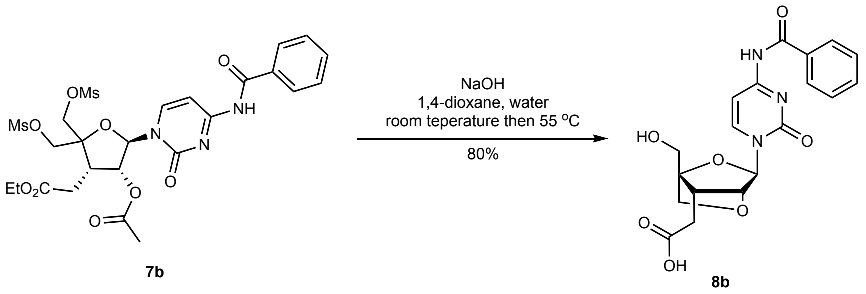

Compound **7b** (400 mg, 0.62 mmol) was dissolved in 1,4-dioxane (4 mL) and 1 M NaOH in water (2 mL, 2 mmol, 3.2 eq) was added. The reaction was stirred at room temperature for 2 h until the locking step and ester hydrolysis was complete (reaction progress monitored using LCMS). The reaction was then heated to 55 °C for 2 h. The reaction was evaporated to dryness and partitioned between CH_2Cl_2 (40 mL) and water (30 mL). The aqueous phase was washed with CH_2Cl_2 (3 x 10 mL), acidified using 1 M HCl and further washed with CH_2Cl_2 (3 x 20 mL). The product was then extracted from the aqueous layer using 25% iPrOH in CH_2Cl_2 (4 x 10 mL, until no product remains in the aqueous layer as determined by TLC), dried over Na_2SO_4, and evaporated to dryness to give **8b** (198 mg, 0.49 mmol) as a white solid in 80% yield which was used without further purification.

TLC (CH_2Cl_2:MeOH, 3:2 v/v, + 2% Et_3N) R_f: 0.4;

^{1}H NMR (500 MHz, d_6-DMSO): δ 8.26 (d, J = 7.5, 1H), 8.00 (dd, J = 8.5, 1.3, 2H), 7.63 (tt, J = 7.5, 1.3, 1H), 7.55 – 7.48 (m, 2H), 7.41 (d, J = 7.5, 1H), 5.55 (s, 1H), 4.45 (s, 1H), 3.82 (d, J = 13.1, 1H), 3.77 (d, J = 13.1, 1H), 3.77 (d, J = 8.4, 1H), 3.65 (d, J = 8.4, 1H), 2.30 (s, 1H), 2.31 – 2.05 (m, 3H);

^{13}C NMR (126 MHz, d_6-DMSO): δ 167.9, 167.4, 163.3, 154.0, 144.3, 133.2, 132.7, 128.5, 128.4, 95.9, 91.2, 87.4, 79.8, 71.1, 56.8, 40.1, 29.9;

HRMS (m/z): $[M-H]^-$ calcd. for $C_{19}H_{18}O_7N_3^-$, 400.1150; found, 400.1141.

N^4-Benzoyl 5-methylcytosine LNA acid **8c**

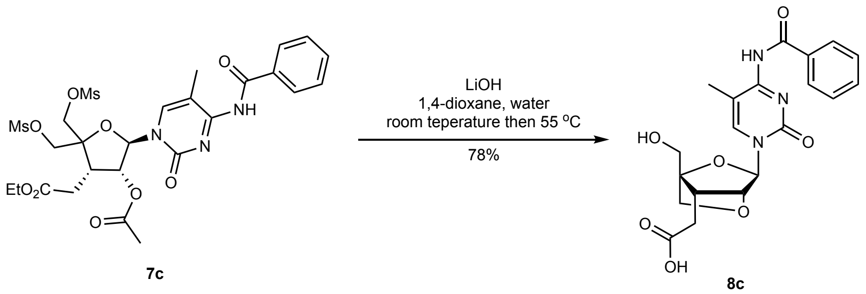

Compound **7c** (400 mg, 0.61 mmol) was dissolved in 1,4-dioxane (4 mL) and 1 M LiOH in water (2 mL, 2 mmol, 3.3 eq) was added. The reaction was stirred at room temperature for 2 h and then heated to 55 °C. After 1 h the reaction was complete as determined by LCMS. The reaction was evaporated to dryness and partitioned between CH_2Cl_2 (40 mL) and water (30 mL). The aqueous layer was washed with CH_2Cl_2 (3 x 40 mL), acidified with 1 M HCl, and washed once more with CH_2Cl_2 (40 mL). The product was then extracted from the aqueous layer using 15% iPrOH in CH_2Cl_2 (5 x 20 mL), until no product remained in the aqueous layer as determined by TLC, dried over Na_2SO_4, and evaporated to dryness to give **8c** (198 mg, 0.48 mmol) as a white solid in 78% yield which was used without further purification.

TLC (CH_2Cl_2:MeOH, 3:2 v/v, + 2% Et_3N) R_f: 0.36;

^{1}H NMR (500 MHz, d_6-DMSO): δ 8.23 – 8.16 (m, 2H), 7.95 (s, 1H), 7.60 (t, J = 7.3, 1H), 7.51 (t, J = 7.6, 2H), 5.53 (s, 1H), 5.25 (br s, 1H), 4.46 (s, 1H), 3.85 (d, J = 13.1, 1H), 3.82 (d, J = 13.1, 1H), 3.77 (d, J = 8.5, 1H), 3.65 (d, J = 8.5, 1H), 2.42 (dd, J = 15.8, 3.2, 1H), 2.30 – 2.19 (m, 2H), 2.04 (s, 3H);

^{13}C NMR (126 MHz, d_6-DMSO): δ 177.9, 172.9, 159.1, 147.2, 137.8, 136.5, 132.5, 129.3, 128.4, 109.1, 91.1, 87.0, 79.5, 71.1, 56.6, 39.0, 28.5, 13.4;

HRMS (m/z): $[M+H]^+$ calcd. for $C_{20}H_{22}O_7N_3^+$, 416.1452; found, 416.1452.

N^6-Benzoyladenine LNA acid **8d**

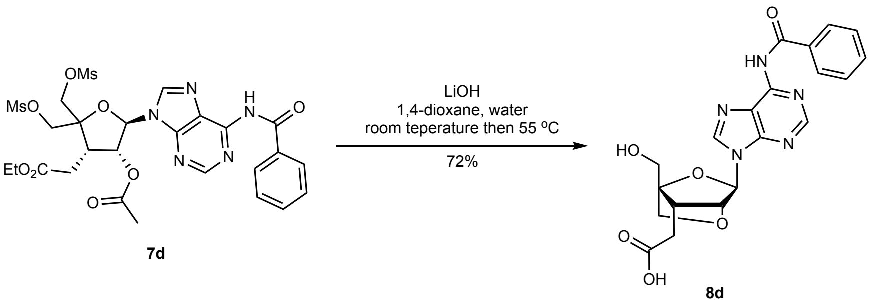

Compound **7d** (500 mg, 0.75 mmol) was dissolved in 1,4-dioxane (4.8 mL) and 1 M LiOH in water (2.4 mL, 2.4 mmol, 3.2 eq). The reaction was stirred at room temperature for 2 h and then heated to 55 °C. After 2 h the product formation was analysed by LCMS. The reaction was not complete and a further 0.33 eq of 1 M LiOH (266 μL) was added and the reaction stirred at 55 °C for 1 h. Once complete, the reaction was evaporated to dryness and partitioned between CH_2Cl_2 (20 mL) and water (20 mL). The aqueous layer was washed with CH_2Cl_2 (3 x 20 mL), acidified with 1 M HCl, and washed once more with CH_2Cl_2 (20 mL). The product was then extracted from the aqueous layer using 15% iPrOH in CH_2Cl_2 (5 x 20 mL), dried over Na_2SO_4, and evaporated to dryness to give **8d** (230 mg, 0.54 mmol) as a white solid in 72% yield which was used without further purification.

TLC (CH_2Cl_2:MeOH, 3:2 v/v, + 2% Et_3N) R_f: 0.24;

^{1}H NMR (400 MHz, d_6-DMSO): δ 12.36 (s, 1H), 11.21 (s, 1H), 8.76 (s, 1H), 8.53 (s, 1H), 8.09 – 8.02 (m, 2H), 7.69 – 7.60 (m, 1H), 7.60 – 7.51 (m, 2H), 6.09 (s, 1H), 4.74 (s, 1H), 3.86 (m, 3H), 3.77 (d, *J* = 8.5, 1H), 3.32 (s, 1H), 2.57 (dd, *J* = 9.8, 4.1, 1H), 2.54 – 2.47 (m, 1H + solvent peak), 2.33 (dd, *J* = 17.1, 9.8, 1H);

^{13}C NMR (101 MHz, d_6-DMSO): δ 173.4, 165.8, 152.3, 151.8, 150.8, 141.5, 133.8, 132.9, 129.0, 128.9, 126.2, 90.8, 86.0, 80.5, 72.0, 57.6, 41.3, 29.1;

HRMS (m/z): $[M+H]^+$ calcd. for $C_{20}H_{20}O_6N_5^+$, 426.1406; found, 426.1407.

N^2-Isobutyrylguanine LNA acid **8e**

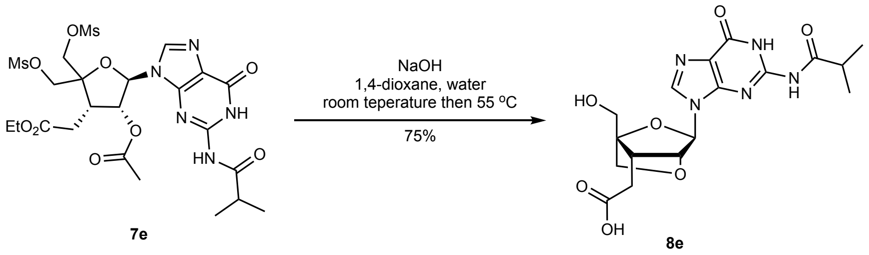

To a solution of compound **7e** (105 mg, 0.17 mmol) in 1,4-dioxane (2 mL) was added 1 M NaOH in water (0.5 mL, 0.5 mmol, 3.0 eq). The reaction was stirred at room temperature for 3 h and then heated to 55 °C. After 1 h the reaction was complete as determined by LCMS. The reaction was evaporated to dryness and was partitioned between CH_2Cl_2 (20 mL) and water (20 mL). The aqueous layer was washed with CH_2Cl_2 (3 x 20 mL), acidified with 1 M HCl, and washed once more with CH_2Cl_2 (20 mL). NaCl was added to saturate the aqueous layer and the product was extracted from the aqueous layer using 25% iPrOH in CH_2Cl_2 (5 x 20 mL), dried over Na_2SO_4, and evaporated to dryness to give **8e** (52 mg, 0.13 mmol) as a white solid in 75% yield which was used without further purification.

TLC (CH_2Cl_2:MeOH, 3:2 v/v, + 2% Et_3N) R_f: 0.18;

^{1}H NMR (400 MHz, d_6-DMSO): δ 12.14 (s, 1H), 11.80 (s, 1H), 8.14 (s, 1H), 5.82 (s, 1H), 4.61 (s, 1H), 3.82 (apparent d, J = 9.8, 3H), 3.69 (d, J = 8.6, 1H), 2.78 (app h, J = 6.8, 1H), 2.55 – 2.46 (m, 2H and d_6-DMSO), 2.29 (dd, J = 17.8, 10.5, 1H), 1.11 (d, J = 6.8, 6H);

^{13}C NMR (101 MHz, d_6-DMSO): δ 180.2, 172.9, 154.6, 148.4, 147.6, 136.1, 120.0, 90.3, 85.3, 80.1, 71.5, 57.1, 40.6, 34.7, 28.6, 18.9, 18.8;

HRMS (m/z): $[M-H]^-$ calcd. for $C_{17}H_{20}O_7N_5^-$, 406.1368; found, 406.1359.

5′-*O*-DMT thymine LNA acid **9a**

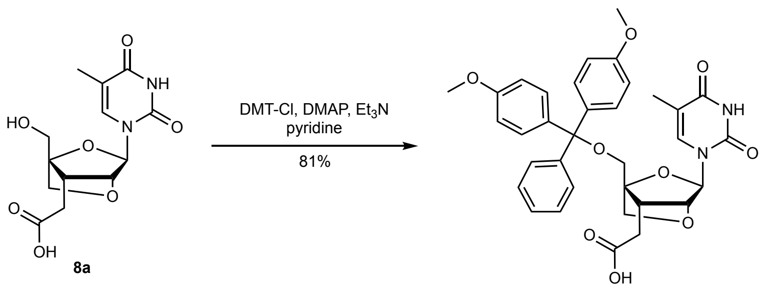

8a 9a

Compound **8a** (400 mg, 1.28 mmol) was dissolved in pyridine (18 mL) and Et_3N (0.25 mL, 1.8 mmol, 1.1 eq) and activated 3 Å molecular sieves were added. The solution was stirred at room temperature for 15 min before 4-dimethylaminopyridine (DMAP) (78 mg, 0.64 mmol, 0.5 eq) and 4,4′-dimethoxytrityl chloride (DMT-Cl) (1 g, 2.95 mmol, 2.3 eq) were added. The reaction was stirred at room temperature for 4 h before a second portion of DMT-Cl (0.8 g, 2.36 mmol, 1.8 eq) was added and the reaction was stirred at room temperature for 16 h. The molecular sieves were removed by filtration and the organic solvents were removed under vacuum. The resulting residue was purified by column chromatography (0-30% MeOH in EtOAc with a constant additive of 2% Et_3N). Following column chromatography, NMR showed significant amounts of Et_3N salts and the material was dissolved in EtOAc (25 mL) and was washed with H_2O (3 x 25 mL), dried over Na_2SO_4, and evaporated to dryness to yield **9a** (687 mg). Rather than risk degradation, the final product was not evaporated to complete dryness and was stored and used with 0.5 eq of Et_3N present (as determined by NMR), making the final yield 81%.

TLC (CH_2Cl_2:MeOH, 4:1 v/v, + 2% Et_3N) R_f: 0.18;

^{1}H NMR (400 MHz, DMSO-d_6): δ 11.43 (s, 1H), 7.61 (d, J = 1.1, 1H), 7.49 – 7.37 (m, 2H), 7.37 – 7.21 (m, 7H), 6.91 (dd, J = 9.0, 3.0, 4H), 5.48 (s, 1H), 4.42 (s, 1H), 3.74 (s, 6H), 3.64 (d, J = 8.6, 1H), 3.60 (d, J = 8.6, 1H), 3.50 (d, J = 11.4, 1H), 3.30 (d, J = 11.4, 1H under water peak), 2.50 – 2.45 (m, 8H, Et_3N counterion under solvent peak), 2.42 (dd, J = 9.4, 4.3, 1H), 2.17 (dd, J = 16.9, 9.4, 1H), 2.00 (dd, J = 16.9, 4.3, 1H), 1.59 (d, J = 1.1, 3H), 0.95 (t, J = 7.2, 4.5H, Et_3N);

^{13}C NMR (151 MHz, d_6-DMSO): δ 172.6, 163.8, 158.2, 149.9, 144.6, 135.2, 135.0, 134.3, 129.7, 129.7, 128.0, 127.6, 126.9, 113.3, 113.3, 108.5, 89.2, 86.6, 85.8, 79.7, 71.3, 58.6, 55.0, 45.5 (Et_3N), 40.7, 28.6, 12.4, 10.7 (Et_3N);

HRMS (m/z): $[M-H]^-$ calcd. for $C_{34}H_{33}O_9N_2^-$, 613.2192; found, 613.2184.

5′-*O*-DMT *N*4-benzoylcytosine LNA acid **9b**

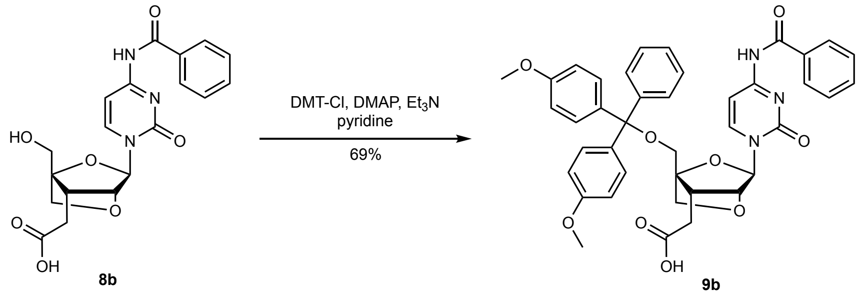

Compound **8b** (100 mg, 0.25 mmol) was dissolved in pyridine (4 mL) and Et_3N (0.05 mL, 0.36 mmol, 1.4 eq) and activated 3 Å molecular sieves were added. The solution was stirred at room temperature for 15 min before DMAP (16 mg, 0.13 mmol, 0.5 eq) and DMT-Cl (204 mg, 0.6 mmol, 2.5 eq) were added. The reaction was stirred at room temperature for 16 h before the molecular sieves were removed by filtration and the organic solvents removed under vacuum. The resulting residue was purified by column chromatography (0-30% MeOH in EtOAc with a constant additive of 2% pyridine) to give **9b** (122 mg) in 69% yield.

TLC (CH_2Cl_2:MeOH, 3:2 v/v, + 2% Et_3N) R_f: 0.37;

^{1}H NMR (400 MHz, DMSO-d_6): δ 11.17 (s, 1H), 8.33 (d, J = 7.5, 1H), 8.01 (d, J = 7.4, 2H), 7.64 (t, J = 7.4, 1H), 7.52 (t, J = 7.6, 2H), 7.46 – 7.22 (m, 10H), 6.94 (d, J = 8.7, 4H), 5.62 (s, 1H), 4.53 (s, 1H), 3.77 (s, 6H), 3.67-3.63 (m, 2H), 3.51 (d, J = 11.1, 1H), 3.41 (d, J = 11.1, 1H), 2.39 (dd, J = 9.3, 4.0, 1H), 2.21 – 2.09 (m, 1H), 1.97 (dd, J= 16.6, 4.0, 1H);

^{13}C NMR (151 MHz, d_6-DMSO): δ 173.2, 168.0. 163.4, 158.2, 154.0, 149.6 (pyridine), 144.4, 143.9, 135.1, 135.1, 133.2, 132.7, 129.7, 129.7, 128.5, 128.4, 128.0, 127.7, 126.9, 123.9, 113.3, 113.3, 95.8, 89.3, 87.6, 86.0, 79.3, 71.3, 58.4, 55.0, 40.1, 28.7.

HRMS (m/z): $[M+H]^+$ calcd. for $C_{40}H_{38}O_9N_3^+$, 704.2603; found, 704.2602.

5′-*O*-DMT *N*4-benzoyl methylcytosine LNA acid **9c**

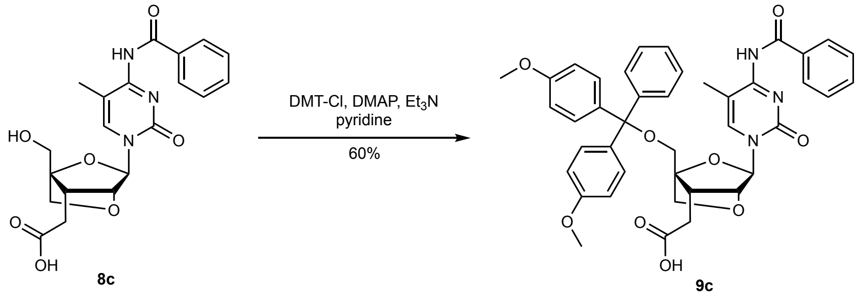

Compound **8c** (100 mg, 0.24 mmol) was dissolved in pyridine (4 mL) and Et_3N (0.05 mL, 0.7 mmol, 1.5 eq) and activated 3 Å molecular sieves were added. The solution was stirred at room temperature for 15 min before DMAP (16 mg, 0.36 mmol, 1.5 eq) and DMT-Cl (204 mg, 0.6 mmol, 2.5 eq) were added. The reaction was left to stir at room temperature for 16 h before the molecular sieves were removed by filtration and the organic solvents removed under vacuum. The resulting residue was purified by column chromatography (0-30% MeOH in EtOAc with a constant additive of 2% pyridine) to yield **9c** (114 mg). Rather than risk degradation, the final product was not evaporated to complete dryness and was stored and used with 1 eq of pyridine present (as determined by NMR), making the final yield 60%.

TLC (CH_2Cl_2:MeOH, 3:2 v/v, + 2% Et_3N) R_f: 0.37;

^{1}H NMR (500 MHz, DMSO-d_6): δ 8.65 – 8.45 (m, 2H, pyridine), 8.22 – 8.12 (m, 2H), 7.89 (s, 1H), 7.78 (tt, J = 7.6, 1.9, 1H, pyridine), 7.60 (t, J = 7.4, 1H), 7.50 (t, J = 7.7, 2H), 7.48 – 7.42 (m, 2H), 7.44 – 7.30 (m, 8H, pyridine), 7.29 – 7.24 (m, 1H), 6.93 (dd, J = 8.9, 4.0, 4H), 5.58 (s, 1H), 4.54 (s, 1H), 3.75 (s, 6H), 3.69 (d, J = 8.7, 1H), 3.66 (d, J = 8.7, 1H), 3.55 (d, J = 11.4, 1H), 3.36 (d, J = 11.4, 1H), 3.34 (br s, 1H), 2.47 (m, 1H under solvent peak), 2.25 (dd, J = 17.0, 8.9, 1H), 2.05 (dd, J = 17.0, 4.4, 1H), 1.87 – 1.75 (m, 3H);

^{13}C NMR (126 MHz, d_6-DMSO): δ 177.6, 172.5, 159.2, 149.6 (pyridine), 147.1, 144.6, 137.1, 136.1 (pyridine), 135.2, 135.1, 132.6, 129.8, 129.3, 129.2, 128.4, 128.0, 127.6, 126.9, 123.9 (pyridine), 113.4, 113.3, 109.2, 89.6, 87.3, 85.9, 79.5, 71.3, 58.5, 55.1, 39.4, 28.4, 13.6;

HRMS (m/z): $[M+H]^+$ calcd. for $C_{41}H_{40}N_3O_9^+$, 718.2759, found, 718.2756.

5′-*O*-DMT N^6-benzoyladenine LNA acid **9d**

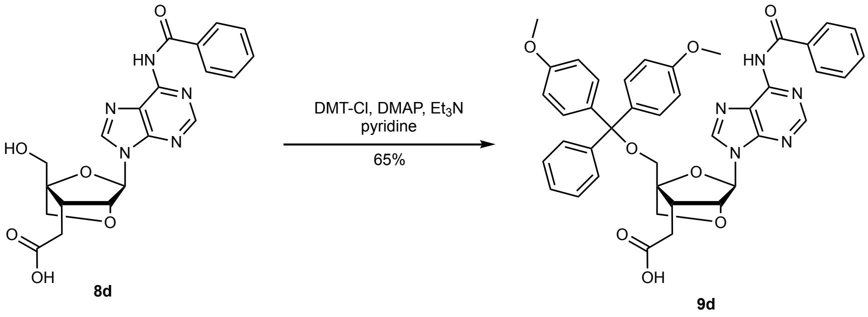

Compound **8d** (62.5 mg, 0.15 mmol) was dissolved in pyridine (1.5 mL) and Et_3N (0.031 mL, 0.22 mmol, 1.5 eq) and activated 3 Å molecular sieves were added. The solution was stirred at room temperature for 15 min before DMAP (18 mg, 2 mmol, 1.3 eq) and DMT-Cl (100 mg, 0.29 mmol, 2 eq) were added. The reaction was stirred at room temperature for 2 h, and a second portion of DMT-Cl (100 mg, 0.29 mmol, 2.0 eq) was added. After 16 h the molecular sieves were removed by filtration and the organic solvents removed under vacuum. The resulting residue was purified by column chromatography (5-15% MeOH in EtOAc with a constant additive of 2% pyridine) to yield **9d** (76 mg). Rather than risk degradation, the final product was not evaporated to complete dryness and was stored and used with 0.72 eq of pyridine present (as determined by NMR), making the final yield 65%.

TLC (EtOAc:MeOH, 3:7 v/v, + 2% Et_3N) R_f: 0.50;

^{1}H NMR (500 MHz, DMSO-d_6): δ 12.63 (s, 1H), 11.27 (s, 1H), 8.78 (s, 1H), 8.60 – 8.55 (m, 1H, pyridine), 8.48 (s, 1H), 8.08 – 8.02 (m, 2H), 7.78 (tt, J = 7.6, 1.9, 0.5H, pyridine), 7.68 – 7.61 (m, 1H), 7.55 (t, J = 7.7, 2H), 7.43 – 7.22 (m, 10H, includes pyridine), 6.90 – 6.85 (m, 4H), 6.14 (s, 1H), 4.81 (s, 1H), 3.84 (d, J = 8.5, 1H), 3.77 (d, J = 8.5, 1H), 3.73 (s, 6H), 3.47 (d, J = 11.2, 1H), 3.44 (d, J = 11.2, 1H), 2.73 (dd, J = 9.7, 4.2, 1H), 2.24-2.16 (m, 1H), 2.08 (dd, J = 16.8, 4.2, 1H);

^{13}C NMR (126 MHz, d_6-DMSO): δ 173.2, 165.7, 158.2, 151.8, 151.4, 150.4, 149.6 (pyridine), 144.6, 140.8, 133.3, 136.1 (pyridine), 135.2, 135.2, 133.4, 132.5, 129.8, 129.7, 128.5, 128.5, 127.9, 127.6, 126.9, 126.8, 125.6, 123.9 (pyridine), 113.3, 88.8, 85.7, 85.6, 80.0, 71.8, 59.5, 55.0, 41.6, 29.2;

HRMS (m/z): $[M+H]^+$ calcd. for $C_{41}H_{38}O_8N_5^+$, 728.2711; found, 728.2715.

5′-*O*-DMT *N*^{2}-isobutyrylguanine LNA acid **9e**

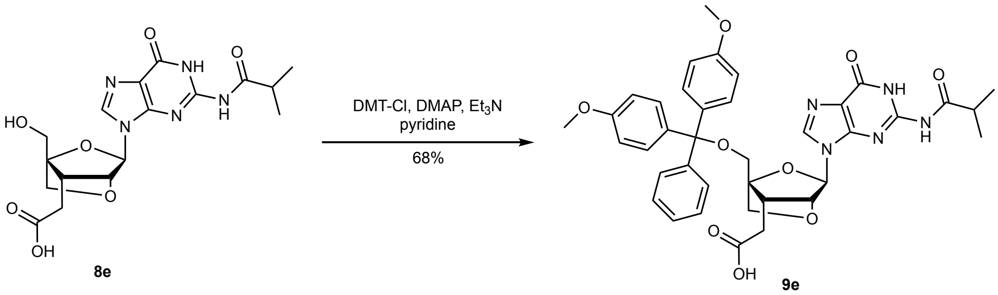

Compound **8e** (41 mg, 0.10 mmol) was dissolved in pyridine (1.7 mL) and Et_3N (0.023 mL, 0.17 mmol, 1.7 eq) and activated 3 Å molecular sieves were added. The solution was stirred at room temperature for 15 min before DMAP (6.7 mg, 0.05 mmol, 0.5 eq) and DMT-Cl (85 mg, 0.25 mmol, 2.5 eq) were added. The reaction was left to stir at room temperature for 16 h. The molecular sieves were removed by filtration and the organic solvents removed under vacuum. The resulting residue was purified by column chromatography (5-15% MeOH in EtOAc with a constant additive of 2% pyridine) to yield **9e** (52 mg). Rather than risk degradation, the final product was not evaporated to complete dryness and was stored and used with 0.65 eq of pyridine present (as determined by NMR), making the final yield 68%.

TLC (EtOAc:MeOH, 3:7 v/v, + 2% Et_3N) R_f: 0.53;

^{1}H NMR (500 MHz, DMSO-d$_6$): δ 8.97 – 8.40 (m, 1.2 H, pyridine), 8.11 (s, 1H), 7.91 – 7.56 (m, 0.6 H, pyridine) 7.39 – 7.34 (m, 3.4H, with 1.4H from pyridine), 7.30 (dd, J = 8.6, 6.9, 2H), 7.29 – 7.17 (m, 5H), 6.93 – 6.84 (m, 4H), 5.85 (s, 1H), 4.71 (s, 1H), 3.76 (d, J = 8.4, 1H), 3.73 (s, 6H), 3.71 (d, J = 8.4, 1H), 3.41 (d, J = 11.1, 1H), 3.31 (d, J = 11.1, 1H), 3.16 (s, 1H, MeOH), 2.79 (p, J = 6.9, 1H), 2.67 (dd, J = 10.2, 3.9, 1H), 2.10 – 1.94 (m, 2H), 1.11 (dd, J = 6.9, 1.9, 6H);

^{13}C NMR (126 MHz, d$_6$-DMSO): δ 180.2, 175.1 (broad), 158.2, 154.9, 148.4, 148.0, 144.7, 136.0, 135.2, 135.1, 129.7, 127.9, 127.6, 126.8, 120.4, 113.3, 113.3, 88.7, 85.6, 85.1, 80.1, 71.9, 59.5, 55.1, 42.5, 34.7, 30.4, 18.9, 18.9;

HRMS (m/z): $[M+H]^+$ calcd. for $C_{40}H_4O_9N_5^+$, 710.2821; found 710.2817.

N_3 Thymine LNA **S14**[4]

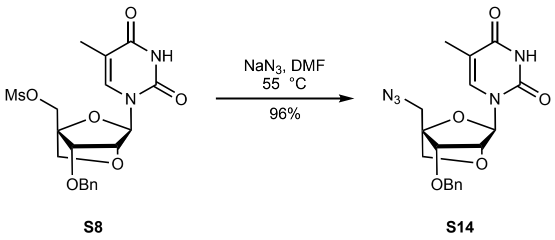

Compound **S14** was prepared based on a similar procedure outlined by Thorpe *et al.*[4] Compound **S8**[1] (3.5 g, 8.0 mmol) and NaN_3 (1.04 g, 16 mmol, 2 eq) were dissolved in DMF (40 mL) and the reaction was stirred at 50 °C for 5 h. Sodium azide is potentially explosive if handled in correctly. The solvent was removed under vacuum and the resulting residue was partitioned between EtOAc (40 mL) and water (40 mL). The organic layer was washed with water (2 x 40 mL), dried over Na_2SO_4, and evaporated to dryness to yield **S14** (2.96 g, 7.7 mmol) as a white solid in 96% yield which was used without purification. If required the compound can be purified by column chromatography (50-100% EtOAc in 40-60 PE).

Data consistent with literature[4].

Amino thymine LNA **S11**[4]

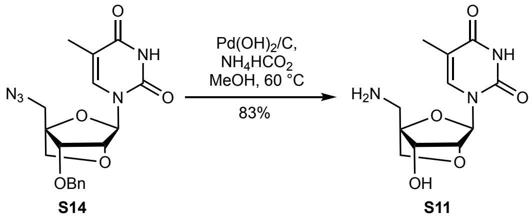

Compound **S11** (2.0 g, 5.2 mmol) and ammonium formate (4.0 g, 63 mmol, 12 eq) were dissolved in MeOH (100 mL) and 20 wt% palladium hydroxide on carbon (0.36 g, 0.52 mmol, 10 mol%) was added. The flask was flushed with argon and the reaction was stirred at 60 °C for 4 h. A large volume of gas is generated within the first hour of the reaction presenting a risk of over-pressurisation. The reaction was filtered through celite to remove the catalyst and the solvent was removed under vacuum. The resulting solid was purified by column chromatography (0-30% MeOH in EtOAc) to give **S11** (1.17 g, 4.3 mmol) as a white solid in 83% yield.

Data consistent with literature[4].

5′-*N*-MMT thymine LNA **S12**[3]

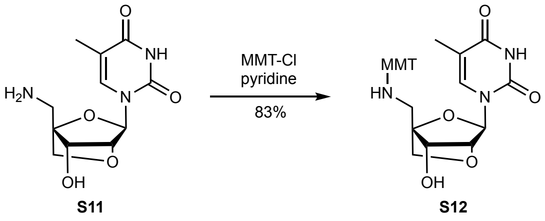

Amine **S11** (1.17 g, 4.3 mmol) was dissolved in anhydrous pyridine (50 mL) and 4-methoxytriphenylmethyl chloride (1.6 g, 5.2 mmol, 1.2 eq) was added in small portions. The reaction was stirred at room temperature for 2 h before the solvents were removed under vacuum. The resulting residue was purified by column chromatography (0-30% EtOAc in 40-60 PE with a constant additive of 0.1% pyridine) to give **S12** (1.93 g, 3.6 mmol) as a pale-yellow foam in 83% yield.

TLC (EtOAc:hexane, 3:2 v/v, + 0.5% pyridine) R_f: 0.5;

^{1}H NMR (400 MHz, $CDCl_3$): δ 9.32 (s, 1H), 7.65 (s, 1H), 7.48 – 7.45 (m, 4H), 7.38 – 7.37 (m, 2H), 7.29 – 7.24 (m, 4H), 7.17 (t, J = 7.3, 2H), 6.81 (d, J= 9.0, 2H), 5.61 (s, 1H), 4.46 (s, 1H), 4.26 (s, 1H), 4.00 (br s, 1H), 3.92 (d, J = 8.3, 1H), 3.78 (d, J = 8.2, 1H), 3.74 (s, 3H), 2.65 – 2.49 (m, 2H), 2.09 (t, J = 8.5, 1H), 1.92 (d, J = 1.2, 3H).

^{13}C NMR (101 MHz, $CDCl_3$): δ 164.1, 158.3, 150.0, 145.9, 145.8, 137.4, 134.7, 129.9, 128.5, 128.2, 126.8, 113.5, 110.5, 88.8, 87.2, 79.8, 72.7, 70.7, 70.4, 55.4, 40.2, 12.8;

HRMS (m/z): $[M+Na]^+$ calcd. for $C_{31}H_{31}O_6N_3Na^+$, 564.2105, found, 564.2103;

No spectroscopic data reported previously[3].

5′-*N*-MMT thymine LNA phosphoramidite **10**[3]

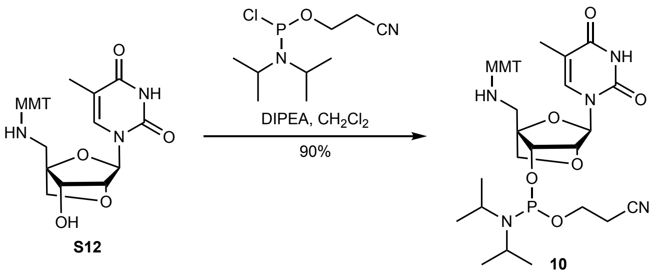

Nucleoside **10** (1.1 g, 2.0 mmol) was dissolved in anhydrous degassed CH_2Cl_2 (10 mL). Degassed *N,N*-diisopropylethylamine (DIPEA) (883 µL, 5.1 mmol, 2.5 eq) and 2-cyanoethyl *N,N*-diisopropylchlorophosphoramidite (677 µL, 3.0 mmol, 1.5 eq) were added and the reaction was stirred under an argon atmosphere at room temperature for 2 h. The reaction mixture was diluted with CH_2Cl_2 (40 mL) and washed with a saturated aqueous solution of KCl (30 mL). The organic phase was dried over Na_2SO_4 and the solvents were removed under vacuum. The resulting pale-yellow oil was purified by column chromatography (40% EtOAc in hexane with a constant additive of 0.5% pyridine) to give the phosphoramidite **10** (1.3 g, 1.8 mmol) as a white foam in 90% yield.

TLC (EtOAc:hexane, 2:3 v/v, + 0.5% pyridine) R_f: 0.4;

^{31}P NMR (162 MHz, $CDCl_3$): δ 148.7, 148.3

HRMS (m/z): $[M-H]^-$ calcd. for $C_{40}H_{47}O_7N_5P^-$, 740.3219; found 740.3219.

Oligonucleotide synthesis

DNA synthesis and cleavage

DNA synthesis was performed on an Applied Biosystems 394 automated DNA/RNA synthesiser using a standard phosphoramidite cycle of detritylation, coupling, capping (unless stated elsewhere), and oxidation on a 1.0 µmole scale. Trichloroacetic acid (TCA) (3% in CH_2Cl_2) was used for detritylation, 5-benzylthio-1*H*-tetrazole (BTT) (0.25 M in MeCN) was used as an activator, and oxidation was achieved using iodine (0.02 M in THF, pyridine and water). Pre-packed nucleoside SynBase™ CPG 1000/110 (Link Technologies) were used and β-cyanoethyl phosphoramidite monomers (dA(Bz), dG(iBu), dC(Bz) and dT, Sigma-Aldrich) were dissolved in anhydrous MeCN (0.1 M) immediately prior to use with coupling time of 50 s. LNA β-cyanoethyl phosphoramidite monomers (QIAGEN) were dissolved to a concentration of 0.1 M in either MeCN (LNA-T) or 25% THF/MeCN (LNA-mC(Bz)) immediately prior to use with a coupling time of 6 min. Stepwise coupling efficiencies were determined by automated trityl cation conductivity monitoring and were >98% in all cases. Cleavage and deprotection were achieved by exposure to concentrated aqueous ammonia solution for 60 min at room temperature followed by heating in a sealed tube for 5 h at 55 °C.

RNA synthesis and cleavage

RNA synthesis was performed on an Applied Biosystems 394 automated DNA/RNA synthesiser using a standard phosphoramidite cycle of detritylation, coupling, capping, and oxidation on a 1.0 µmole scale. Coupling, capping and oxidation reagents were identical to those used for DNA synthesis except a solution of ethylthiotetrazole (ETT) (0.25 M in MeCN, Link Technologies) was used instead of BTT as the activator. Standard CPG resin (Link Technologies) was used and 2'-thiomorpholine-4-carbothioate (TC) protected monomers (A(Bz), C(Ac), G(iBu) and U, Sigma-Aldrich) were dissolved in anhydrous toluene/MeCN (1:1 v/v, 0.1 M) immediately prior to use. The coupling time for all monomers was 3 min. Stepwise coupling efficiencies were determined by automated trityl cation conductivity monitoring and in all cases were >97%. To deprotect and cleave the RNA, the solid support was exposed to dry ethylenediamine:toluene (1:1 v/v) for 6 h at room temperature, washed with toluene (3 x 1 mL), then MeCN (3 x 1 mL) and dried using argon. The cleaved RNA was eluted from the solid support with water.

2’OMe phosphodiester oligonucleotide synthesis and cleavage

2’OMe oligonucleotides were synthesised on an Applied Biosystems 394 automated DNA/RNA synthesiser using a standard phosphoramidite cycle of detritylation, coupling (unless otherwise stated), capping, and oxidation on a 1.0 µmole scale. Detritylation, coupling, capping, oxidation and activation reagents are identical to those used for DNA synthesis. Pre-packed nucleoside SynBase™ CPG 1000/110 (Link Technologies) were used, and β-cyanoethyl phosphoramidite monomers (DMT-2’O-methyl-rA(Bz), DMT-2’O-Methyl-rG(iBu), DMT-2’O-Methyl-rC(Ac) and DMT-2’O-Methyl-rU, Sigma-Aldrich) were dissolved in anhydrous MeCN (10% CH_2Cl_2 was added when 2’OMe U phosphoramidite was used) to a concentration of 0.1 M immediately prior to use with a coupling time of 6 min. LNA β-cyanoethyl phosphoramidite monomers (QIAGEN) were dissolved to a concentration of 0.1 M in either MeCN (LNA-T) or 25% THF/MeCN (LNA-mC(Bz)) immediately prior to use with a coupling time of 6 min. Stepwise coupling efficiencies were determined by automated trityl cation conductivity monitoring and were >98% in all cases. Cleavage and deprotection were achieved by exposure to concentrated aqueous ammonia solution for 60 min at room temperature followed by heating in a sealed tube for 5 h at 55 °C.

Phosphodiester oligonucleotide purification

Oligonucleotides were purified using a Gilson reverse-phase high performance liquid chromatography (RP-HPLC) system with ACE® C8 column (particle size: 10 µm, pore size: 100 Å, column dimensions: 10 mm x 250 mm) with a gradient of buffer A (0.1 M TEAB, pH 7.5) to buffer B (0.1 M TEAB, pH 7.5 containing 50% v/v MeCN) and flow rate of 4 mL/min. The gradient of MeCN in triethylammonium bicarbonate (TEAB) was increased from 0% to 50% buffer B over 30 min. Elution was monitored by UV absorbance at 298 nm. After HPLC purification, oligonucleotides were freeze dried then dissolved in water without the need for desalting.

Phosphorothioate oligonucleotide synthesis, cleavage and purification

Oligonucleotides with a phosphorothioate rather than a phosphodiester backbone were synthesised as described above, except for a solution of 3-ethoxy-1,2,4-dithiazoline-5-one (EDITH, Link Technologies) in MeCN (0.05 M) was used as a sulfurising reagent in place of the oxidising solution. The sulfurisation time was extended to 3 min followed by sending fresh EDITH to the synthesis column and leaving it for another 3 min. Phosphorothioate modified oligonucleotides were isolated with the final 5′-DMT protecting group still in place (DMT-On). Following solid phase synthesis, the cyanoethyl groups were removed by a 15 min treatment with 20% diethylamine in MeCN. The resin was then washed with MeCN (5 x 1 mL) and dried by passing a stream of argon through the synthesis column. The oligonucleotides were cleaved from the solid support and deprotected by heating in a sealed glass vial at 55 °C for 5 h. The ammonia was removed under reduced pressure prior to oligonucleotide purification. The DMT-On oligonucleotides were purified by RP-HPLC and lyophilised. They were then dissolved in 0.5 mL of 80% acetic acid and incubated for 1 h at room temperature to remove the DMT group. The solution was neutralised with 0.5 mL of triethylammonium acetate buffer (2 M, pH 7) and the detritylated oligonucleotides were desalted using a NAP-10 column (Cytiva) then freeze dried.

Oligonucleotide analysis

All oligonucleotides were characterised by negative-mode ultra-performance liquid chromatography (UPLC) mass spectrometry using a Waters Xevo G2-XS QT of mass spectrometer with an Acquity UPLC system, equipped with an Acquity UPLC oligonucleotide BEH C18 column (particle size: 1.7 µm; pore size: 130 Å; column dimensions: 2.1 mm x 50 mm). Data were analysed using Waters MassLynx software v 4.1 or Waters UNIFI Scientific Information System software.

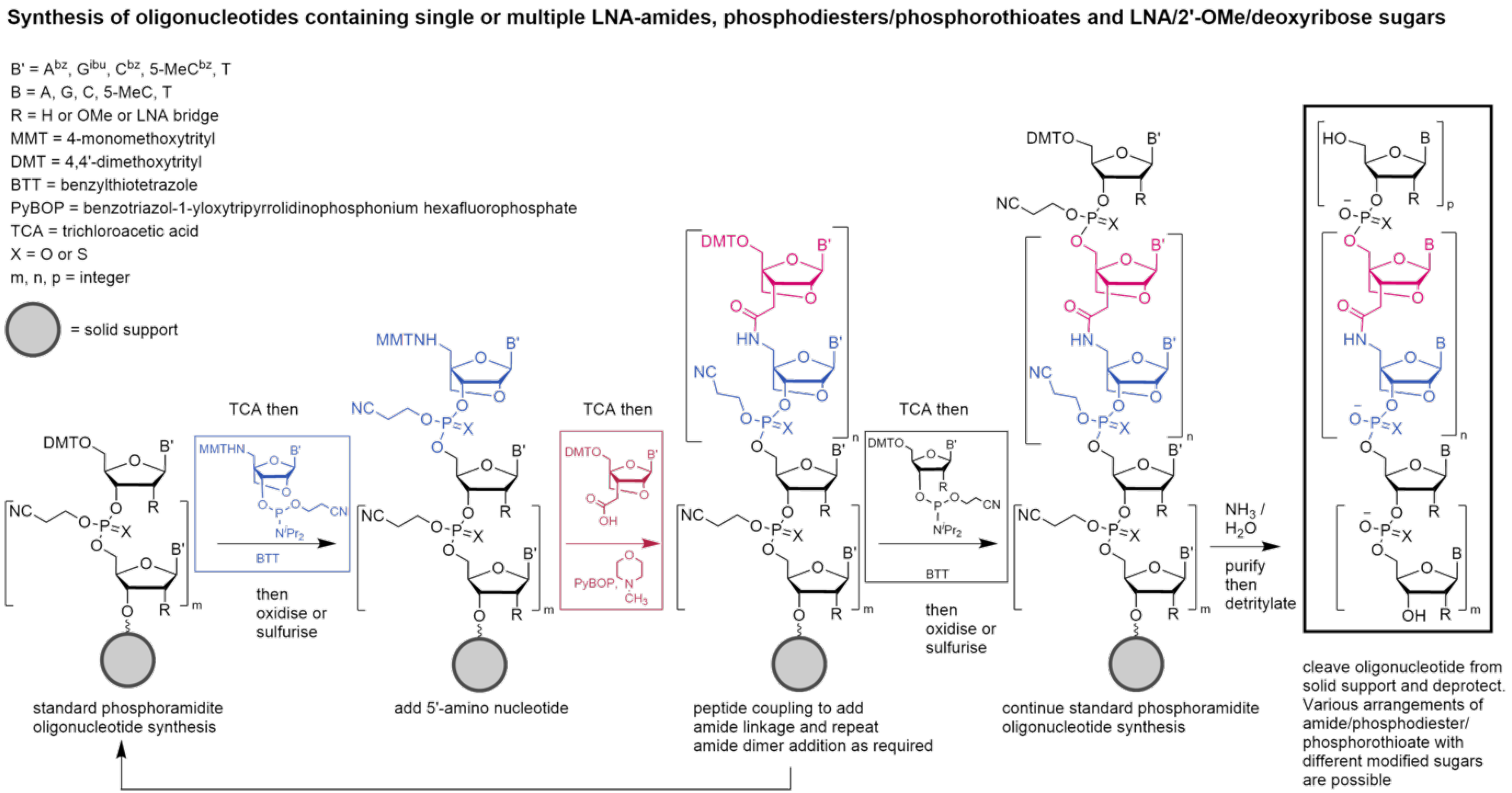

Supplementary Figure 42. Schematic overview of LNA amide modified oligonucleotide synthesis. Protocols for individual steps are outlined below.

LNA-amide modified oligonucleotide synthesis

Oligonucleotide segment synthesis

Oligonucleotide segments were synthesised as described, except that the capping step was omitted.

Amino monomer addition

The MMT-protected 5´-amino phosphoramidite monomer (either LNA **10**[3] or commercially available deoxythymidyl **11**) was dissolved in anhydrous MeCN (0.1 M) immediately prior to use. The same conditions as above were used, but the coupling time was extended to 10 min. No capping step was used. The 5´-MMT protecting group was cleaved on the Applied Biosystems 394 automated synthesiser using TCA (3% in CH_2Cl_2) with an extended cleavage time of 2 min. The solid support was then washed with MeCN on the synthesiser for 3 min. To improve the coupling efficiency in the next step the solid support was washed with *N*-methylmorpholine in DMF (0.5% v/v, 1 mL) followed by DMF (3 x 1 mL).

Amide bond formation on resin (peptide coupling)

All amide couplings were performed manually in the synthesis column. A solution with 10 equivalents of acid monomer, 10 equivalents of PyBOP and 30 equivalents of *N*-methylmorpholine was first prepared in 400 µL of DMF. This was then taken up into a 1 mL syringe and loaded into the column before a second 1 mL syringe was attached to the other end of the synthesis column. The mixture was agitated every 10 min for 1 h. The columns were then washed with DMF (3 x 1 mL) followed by MeCN (5 x 1 mL) and dried by passing argon through the column. The column was then returned to the synthesiser to continue oligonucleotide synthesis.

Cleavage of oligonucleotides from resin, deprotection and purification

LNA-amide containing oligonucleotides were isolated with the final 5′-DMT protecting group still in place (DMT-On). Following solid phase synthesis, the cyanoethyl groups were removed by a 15 min treatment with 20% diethylamine in MeCN. The resin was then washed with MeCN (5 x 1 mL) and dried by passing a stream of argon through the synthesis column. The oligonucleotides were cleaved from the solid support and deprotected by heating in concentrated aqueous ammonia solution in a sealed glass vial at 55 °C for 5 h. The ammonia was removed under reduced pressure prior to oligonucleotide purification. The DMT-On oligonucleotides were purified by RP-HPLC. The elution of oligonucleotides was monitored by UV absorbance at 298 nm. The oligonucleotides were lyophilised and then dissolved in 0.5 mL of 80% acetic acid, and incubated for 1 h at room temperature to remove the DMT group. The solution was neutralised with 0.5 mL of triethylammonium acetate buffer (2 M, pH 7) and the detritylated oligonucleotides were desalted using a NAP-10 column (Cytiva), then freeze dried.

Biophysical studies

UV melting experiments

UV melting experiments were performed using a Cary 4000 scan UV-vis spectrophotometer. 3 nmol of each oligonucleotide was dissolved in 1 mL of 10 mM phosphate buffer containing 200 mM NaCl at pH 7.0. The samples were first denatured by heating to 85 °C (10 °C/min) and then annealed by slowly cooling to 20 °C (1 °C/min). Six successive cycles of heating and cooling were performed at a gradient of 1 °C/min whilst recording the change in UV absorbance at 260 nm. The built-in Cary WinUV 3.0 software was then used to calculate the melting temperature from the first derivative of the melting curve.

Oligonucleotide X-Ray crystallography

Crystallisation

DNA and RNA oligonucleotides were purified by HPLC, desalted by gel filtration (NAP-10) and then freeze dried. Oligonucleotide stock solutions (2 mM) were prepared in aqueous KCl (10 mM). DNA samples were combined with an equimolar ratio of complementary RNA to form their respective modified DNA:RNA hybrids to form 1 mM duplex (60 µL). Single crystals of the DNA:RNA duplexes were obtained by the sitting drop vapour diffusion method. The Natrix HT sparse matrix screen (Hampton Research, HR2-131) was used to identify crystallisation hits for each modified duplex sample using high throughput (HT) methods. All HT screens were performed in CrystalMation Intelli-Plate 96-3 low-profile plates (Hampton Research, HR3-119). Reservoirs and drops were dispensed using an Art Robbins Phoenix automatic liquid handler. Reservoirs contained 80 µL of Natrix HT solution and crystallisation drops (200 - 300 nL total volume) were placed in each of the three subwells; subwell 1, 200 nL oligo :100 nL well solution; subwell 2, 100 nL oligo : 100 nL well solution; subwell 3, 100 nL oligo: 200nL well solution (stock duplex concentration was 1 mM). Plates were sealed using optically clear Xtra-Clear Advanced Polyolefin StarSeal (StarLab) and incubated at 19 °C, crystals usually formed within one week (range 2-90 days, crystal size < 10 - 200 µm). The unmodified DNA:RNA duplex was crystallised using adapted conditions from Kopka *et al.*[8] Optimisation of these conditions were done in 24 well Cryschem sitting drop plates (Hampton Research, USA) using 4 µL sitting drops consisting of 0.5 mM duplex, 12 mM $Mg(OAc)_2$, 0.6 mM spermidine.HCl, 0.075% (w/v) β-octylglucoside, 12 mM sodium cacodylate and 12% 2-methyl-2,4-pentanediol (MPD). This was equilibrated against a reservoir of H_2O:MPD (1:1 v/v, 400 µL). To screen conditions, components of the drop were varied (6-16 mM $Mg(OAc)_2$, 0.2-1.2 mM spermidine.HCl, 0.075% (w/v) β-octylglucoside, 12 mM sodium cacodylate and 6-16% MPD. All other structures were obtained using hits from the NatrixHT screen (Hampton Research, USA). All samples were crystallised at 19 °C using the conditions outlined in Supplementary Table 6.

Data collection and processing

Sample wells were opened and cryo protectant 20% glycerol in reservoir solution (2 µL) was added. Crystals were harvested using cryoloops (0.01-0.05 mm) and immediately cryo-cooled by plunging into liquid N_2 (77 K), transferred into a cryo-vial and stored under liquid nitrogen at 77 K until data collection. Data collection was performed at Diamond Light Source (beamlines i03 or i04) or DESY in Hamburg (beamline P13). The high radiation damage resistance of the oligo duplex crystals permitted 100% beam transmission. Oscillation images (3600 images, 0.1 ° osc) were collected. The detector distance was set to obtain a maximum resolution of 0.5 Å greater than the expected diffraction limit to maximise spot separation (see Supplementary Table 5) and reduce overlapping reflections and obtain maximal completeness. Data were auto processed using either fast_dp v1.6.2[9], xia2_dials (xia2 v0.6.475, dials v2.2.10)[10] or xia2_3dii (xia2 v0.6.475, XDS v20210205)[11]. $CC_{1/2}$ > 0.3 and completeness > 90%, crystal data quality was reviewed using Phenix.Xtriage. $ON26^{xDNA}$, $ON29^{xDNA\text{-}Am\text{-}DNA}$, and $ON29^{xLNA\text{-}Am\text{-}DNA}$ duplexes all crystallised in the high symmetry space group $P\ 6_1$ and contained a single DNA:RNA hybrid in the asymmetric unit. In contrast, the $ON30^{xLNA\text{-}Am\text{-}LNA}$ duplex was in the lower symmetry Space Group $P\ 3_221$ with two DNA:RNA hybrids in each asymmetric unit.

Structure solution, model building and refinement

The structures were solved using the Molecular Replacement method and 1PJO PDB ID as the search model[12, 13] using PHASER 2.8.2[14]. Structure solutions resulted in TFZ score > 8.0 and LLG > 50 and correct solution was confirmed by visual inspection of electron density maps. The DNA:RNA models (some with modified backbone) were built and fit to the electron density using winCOOT v 0.9.2[15]. Model refinement was performed using REFMAC5 v5.0.32[16] and PHENIX.REFINE with Phenix software suite v1.19.2[17]. Geometric restraints for the non-standard phosphoribosyl backbones were generated using JLIGAND v 1.0.40[12] or ACEDRG 222[18]. Model building continued until the observed electron density was satisfied and the R_{free} no longer decreased. Software packages and project management was handled using CCP4 v 7.0.065[19] and Phenix[17]. Images were made using PYMOL graphic software (The PyMOL Molecular Graphics System, Version 2.3.2 Schrödinger, LLC).

Where necessary, data were reprocessed to achieve acceptable final statistics (i.e. CC ½ > 0.3). Reprocessing was performed using iMosflm, XDS or in-house using automated xia2 pipelines[11]. The data were then scaled and merged using Aimless v 0.7.3[20].

Biological assays

Evaluation of stability in fetal bovine serum (FBS)

Five nmol of each oligonucleotide was dissolved in Dulbecco's PBS (50 µL) and FBS (50 µL, Gibco, standard sterile-filtered) was added. The sample was mixed by pipetting and 20 µL of this solution was immediately removed, mixed with formamide (20 µL), snap frozen in liquid N_2, and stored at −80 °C as a control (0 h). The remaining reaction mixtures were incubated at 37 °C and aliquots (20 µL) were taken at different time intervals, mixed with formamide (20 µL), snap frozen in liquid N_2 and stored at −80 °C. The samples were then analysed by denaturing 20% polyacrylamide gel.

Cell culture

HeLa pLuc/705 cells[21] were cultured in Dulbecco's Modified Eagle Medium with GlutaMAX-I (Gibco) supplemented with 10% (v/v) FBS (Gibco) and 1 x Antibiotic-Antimycotic (Gibco) at 37 °C in a humidified incubator with 5% CO_2.

Transfection with Lipofectamine 2000

Cells were seeded at a density of 7000 cells/well in 100 µL of culture media in 96 well plates 16 h before transfection to reach 70-80% cell confluency. Immediately prior to transfection, 5 µL of Lipofectamine 2000 (Invitrogen) was diluted in 500 µL OptiMEM (Gibco) and incubated at room temperature for 5 min before mixing with 4 nmol of lyophilised oligonucleotide dissolved in 500 µL of OptiMEM. The resulting mixture was incubated at room temperature allowing complexation to occur. The complexes were then further diluted in OptiMEM to the concentrations required for the experiments. Culture media was removed from the cells and 100 µL of the complexes added per well. The cells were then incubated at 37 °C in a humidified incubator with 5% CO_2. After 4 h the media was replaced with 100 µL of culture media and the cells were returned to the incubator for a further 20 h.

Gymnosis experiments

Cells were seeded at a density of 800 cells/well in 100 µL in culture media in 96 well plates 16 h before the oligonucleotides were added. Lyophilised oligonucleotides were dissolved in OptiMEM immediately before addition to the cells. The media in each well was removed and replaced with 100 µL of the oligonucleotide containing OptiMEM. The cells were then incubated for 96 h at 37 °C in a humidified incubator with 5% CO_2.

Luciferase assay

The culture media was removed from the well and the cells were washed with 200 µL of PBS. 100 µL of GloLysis™ buffer (Promega) was added to each well. The plate was incubated at room temperature on the orbital shaker for 10 min to lyse the cells. 50 µL of the cell lysate was added to 50 µL of Bright-Glo™ luciferase reagent (Promega) in a white 96 well plate and the luminescence was measured using a CLARIOstar microplate reader (BMG Labtech) and analysed with CLARIOstar software version 5.21.R2. 25 µL of the cell lysate was then used for protein quantification using a Pierce BCA protein assay kit in accordance with the manufacturer's guidelines, using GloLysis buffer as a blank standard. The luminescence values were divided by the total protein quantities and normalised to the values for untreated cells.

WST-1 cell viability assay

The cell viability was evaluated using the WST-1 cell proliferation reagent (Roche) in accordance with the manufacturer's guidelines. Briefly, cells were seeded, transfected using Lipofectamine 2000, and the media was changed to culture media after 4 h, as described above. The cells were then incubated for 20 h at 37 °C

in a humidified incubator with 5% CO_2 before WST-1 reagent (10 µL) was added to each well. The cells were returned to the incubator for 4 h. The cells were shaken at 500 rpm for 1 min before 10 µL of media was removed from each well and added to a clear 96 well plate containing 90 µL of water in each well and the absorbance at 440 nm was measured using a CLARIOstar microplate reader (BMG Labtech) and analysed with CLARIOstar software version 5.21.R2. This dilution step was necessary as the absorbance went above the accurate range of the instrument. Cells that were treated with OptiMEM instead of the oligonucleotide complexes were used as a 100% viability reference.

NMR spectra of compounds 2-10

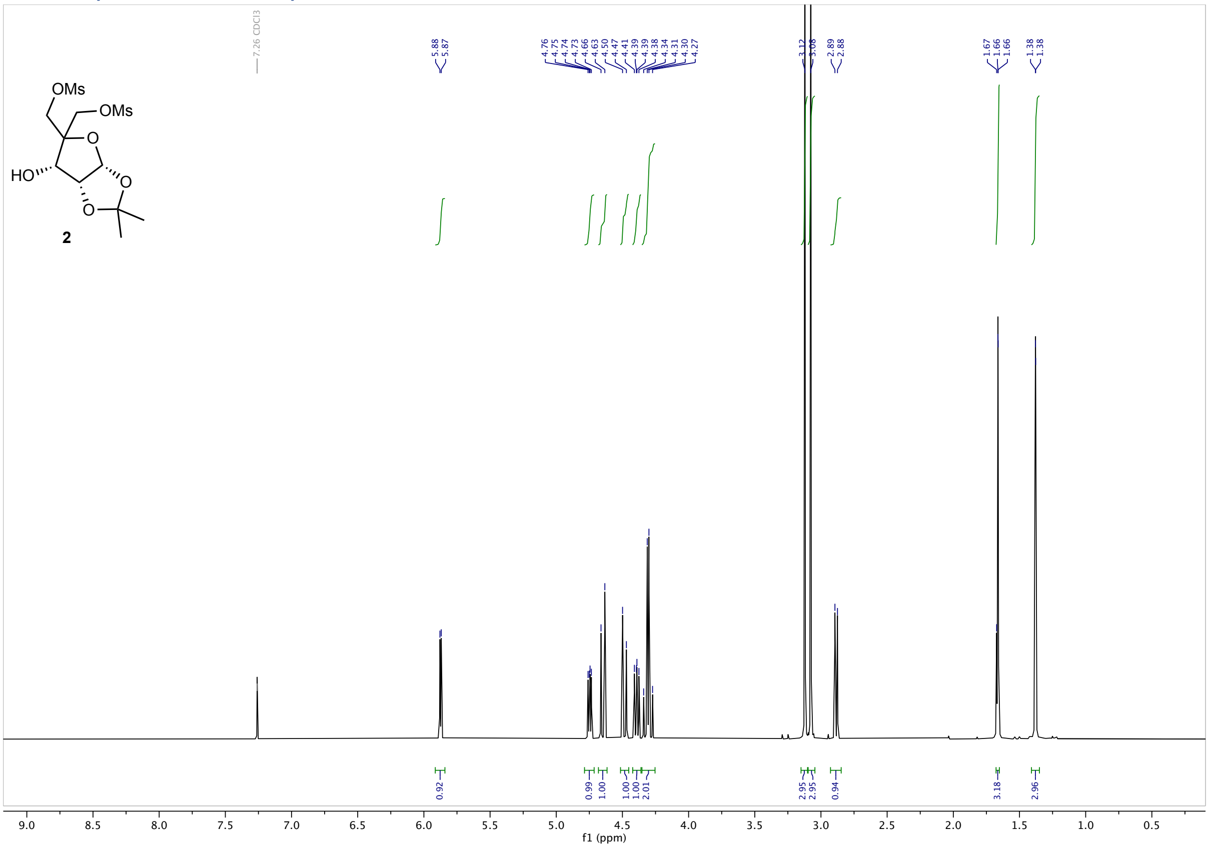

Supplementary Figure 43. ^{1}H NMR (400 MHz, $CDCl_3$) spectra of compound **2**.

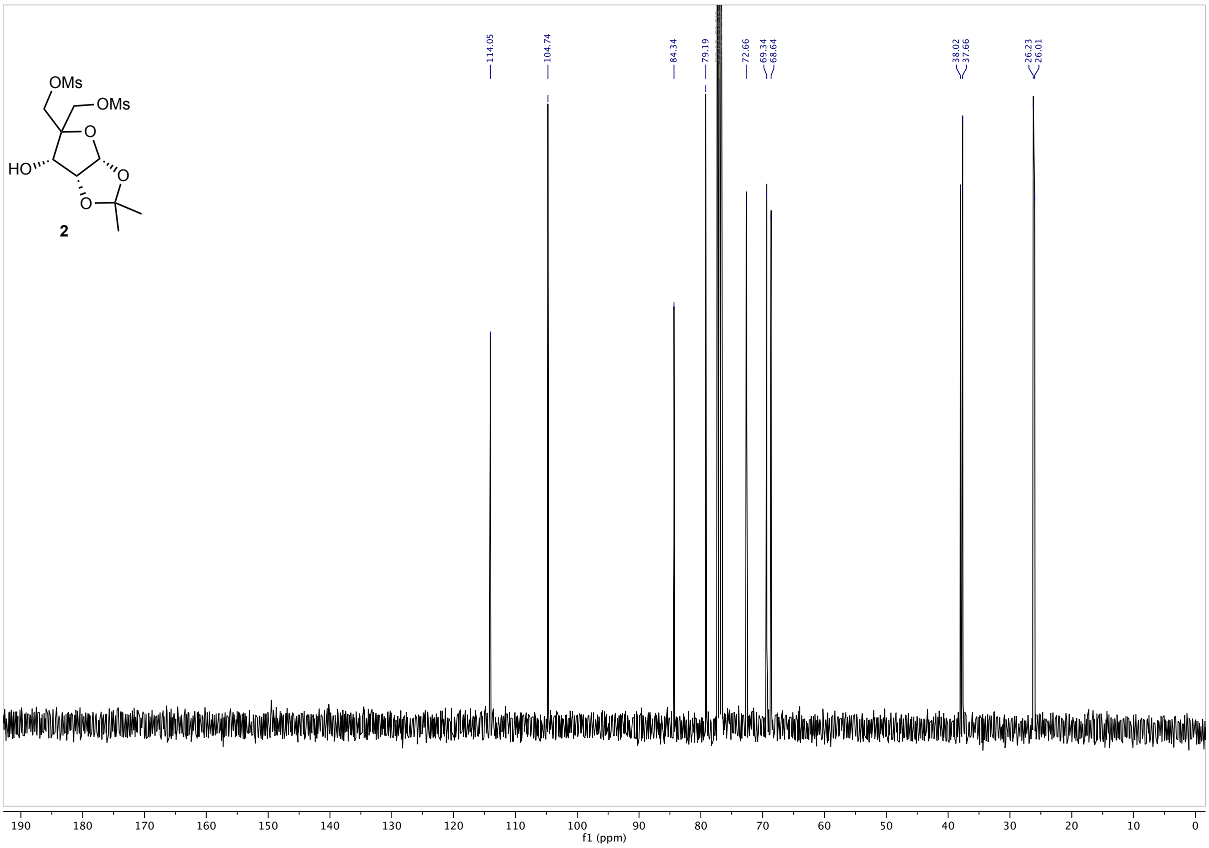

Supplementary Figure 44. ^{13}C NMR (101 MHz, $CDCl_3$) spectra of compound **2**.

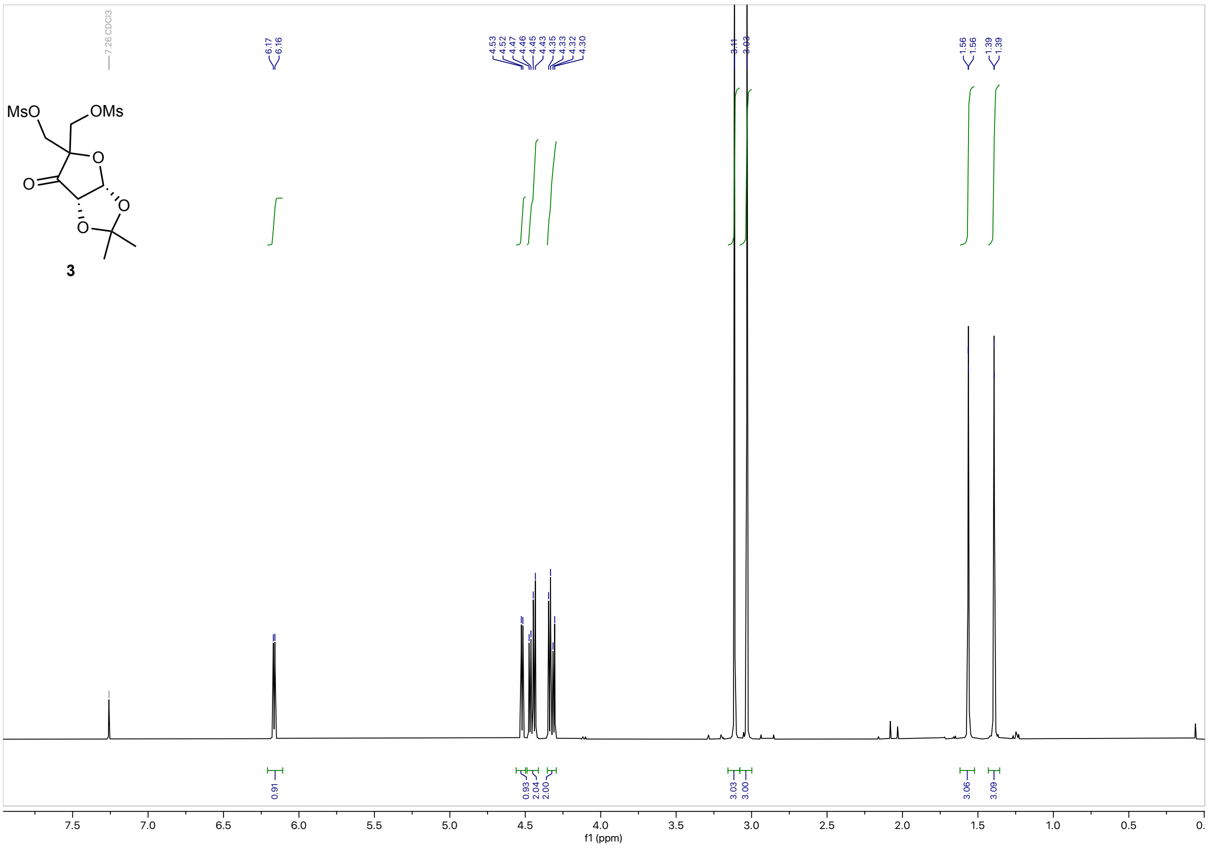

Supplementary Figure 45. ^{1}H NMR (400 MHz, $CDCl_3$) spectra of compound **3**.

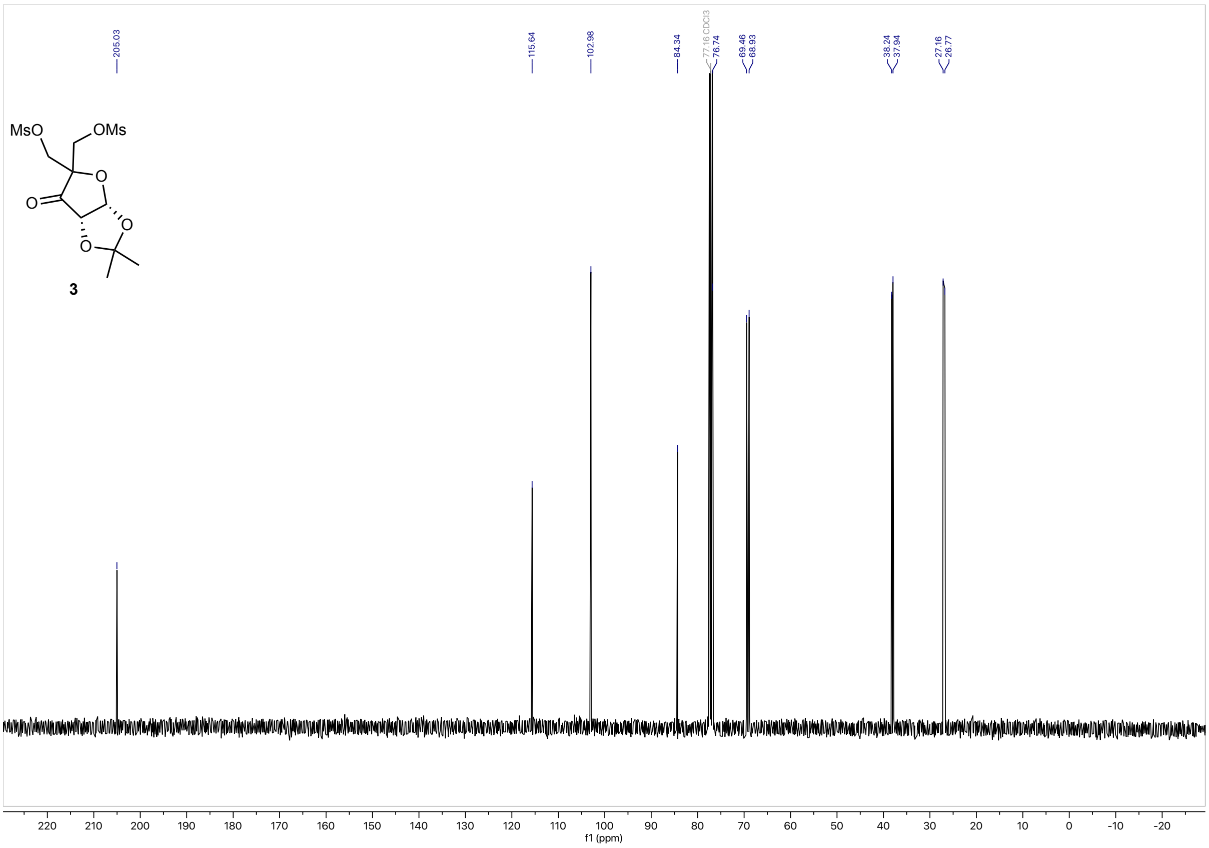

Supplementary Figure 46. ^{13}C NMR (101 MHz, $CDCl_3$) spectra of compound **3**.

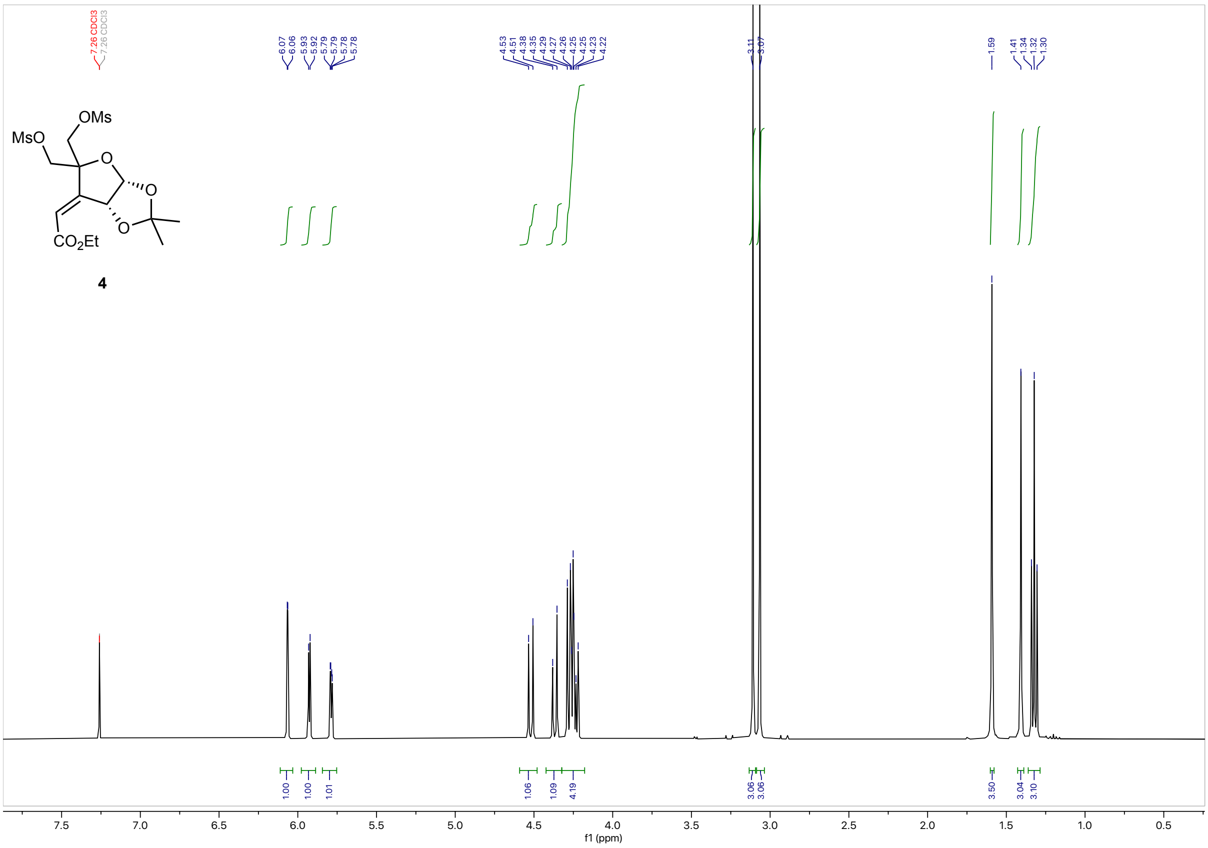

Supplementary Figure 47. ^{1}H NMR (400 MHz, $CDCl_3$) spectra of compound **4**.

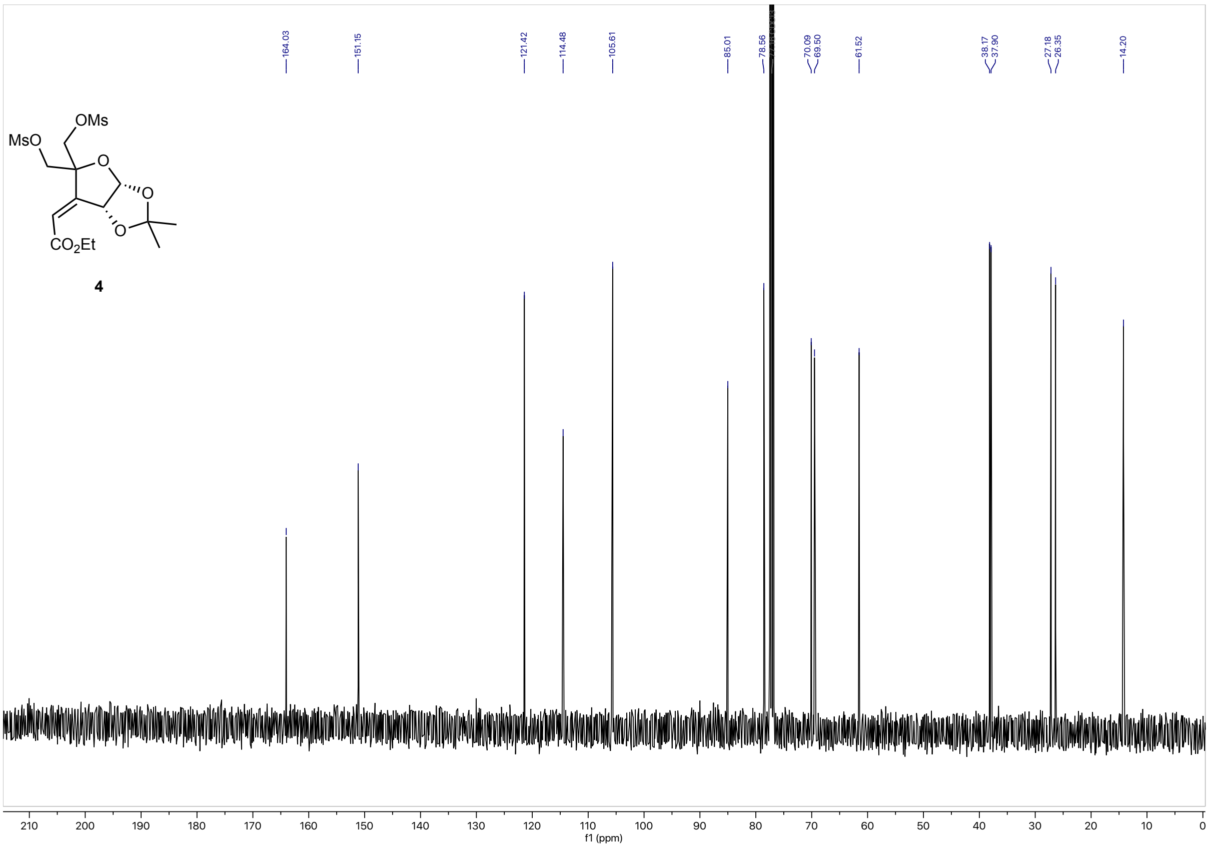

Supplementary Figure 48. ^{13}C NMR (101 MHz, $CDCl_3$) spectra of compound **4**.

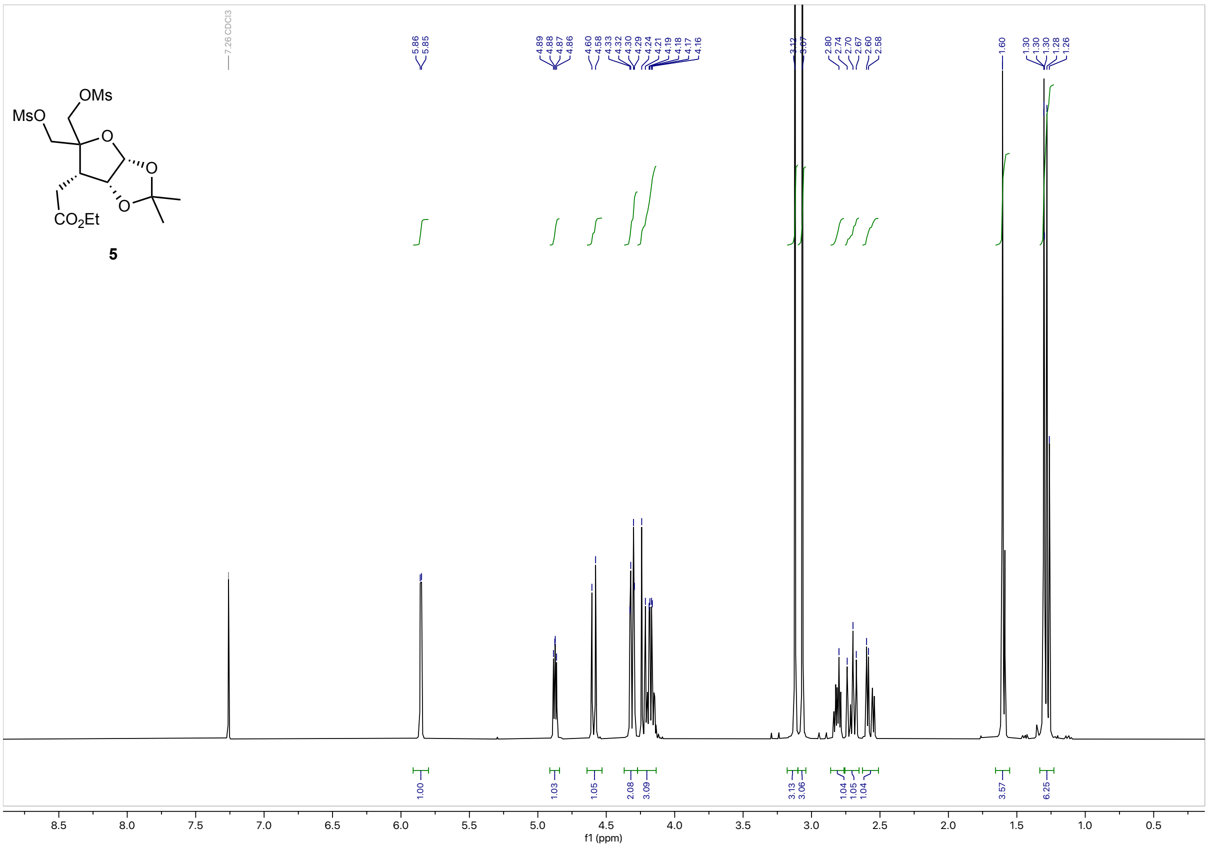

Supplementary Figure 49. ^{1}H NMR (400 MHz, $CDCl_3$) spectra of compound **5**.

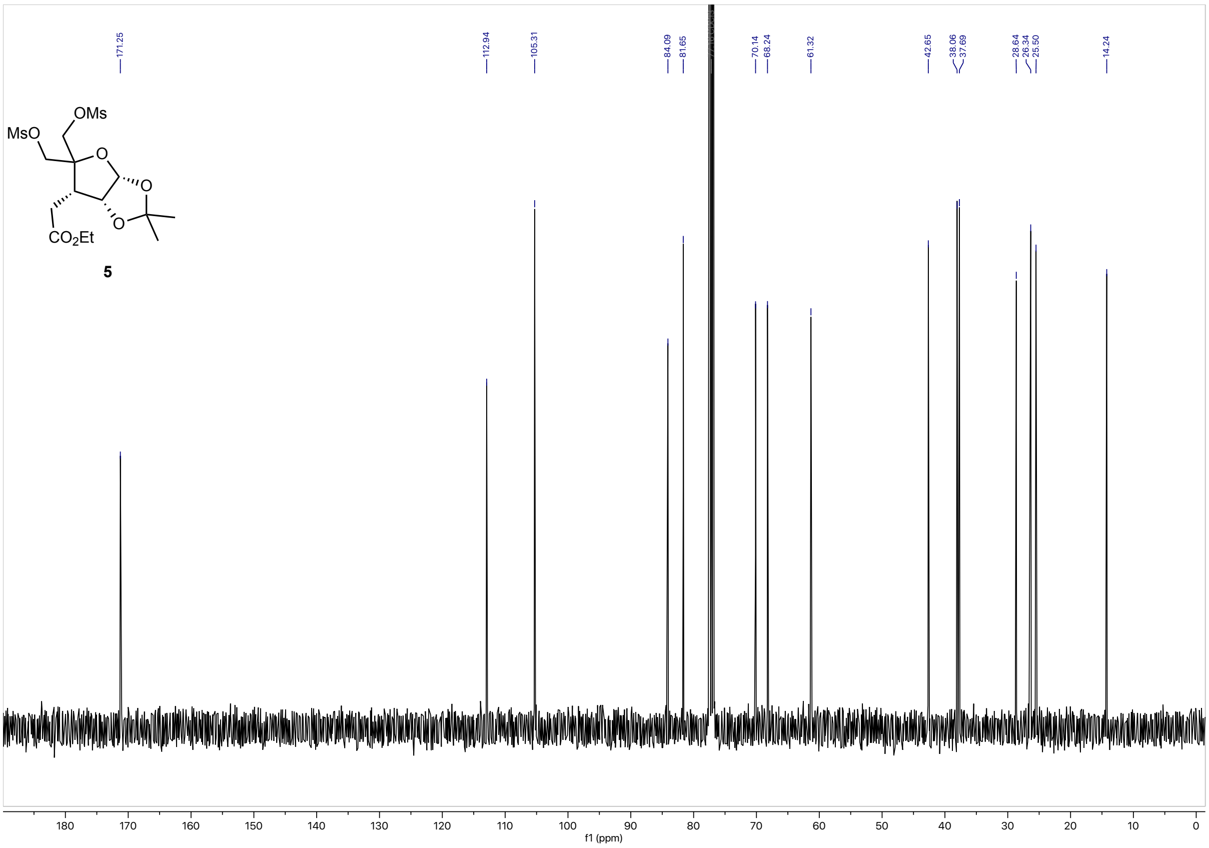

Supplementary Figure 50. ^{13}C NMR (101 MHz, $CDCl_3$) spectra of compound **5**.

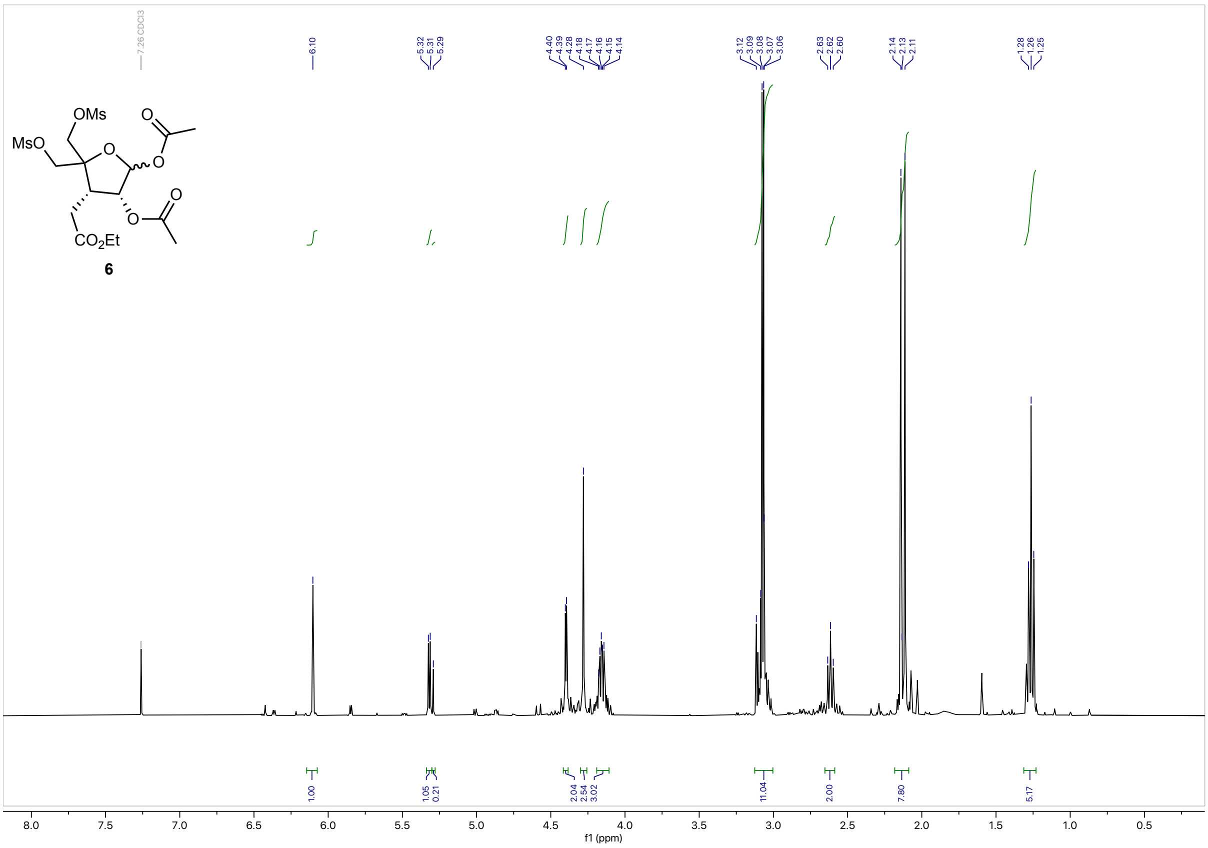

Supplementary Figure 51. ^{1}H NMR (400 MHz, $CDCl_3$) spectra of compound **6** (crude).

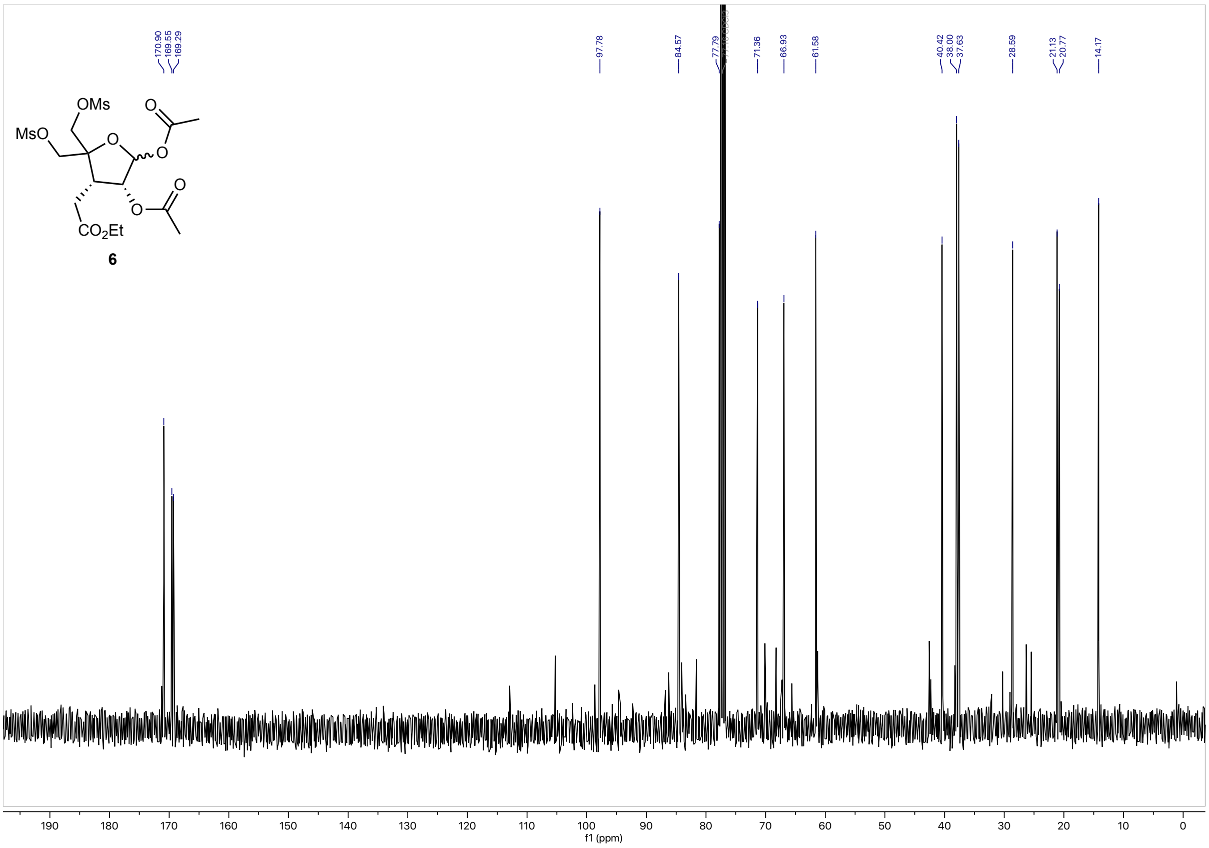

Supplementary Figure 52. ^{13}C NMR (101 MHz, $CDCl_3$) spectra of compound **6** (crude).

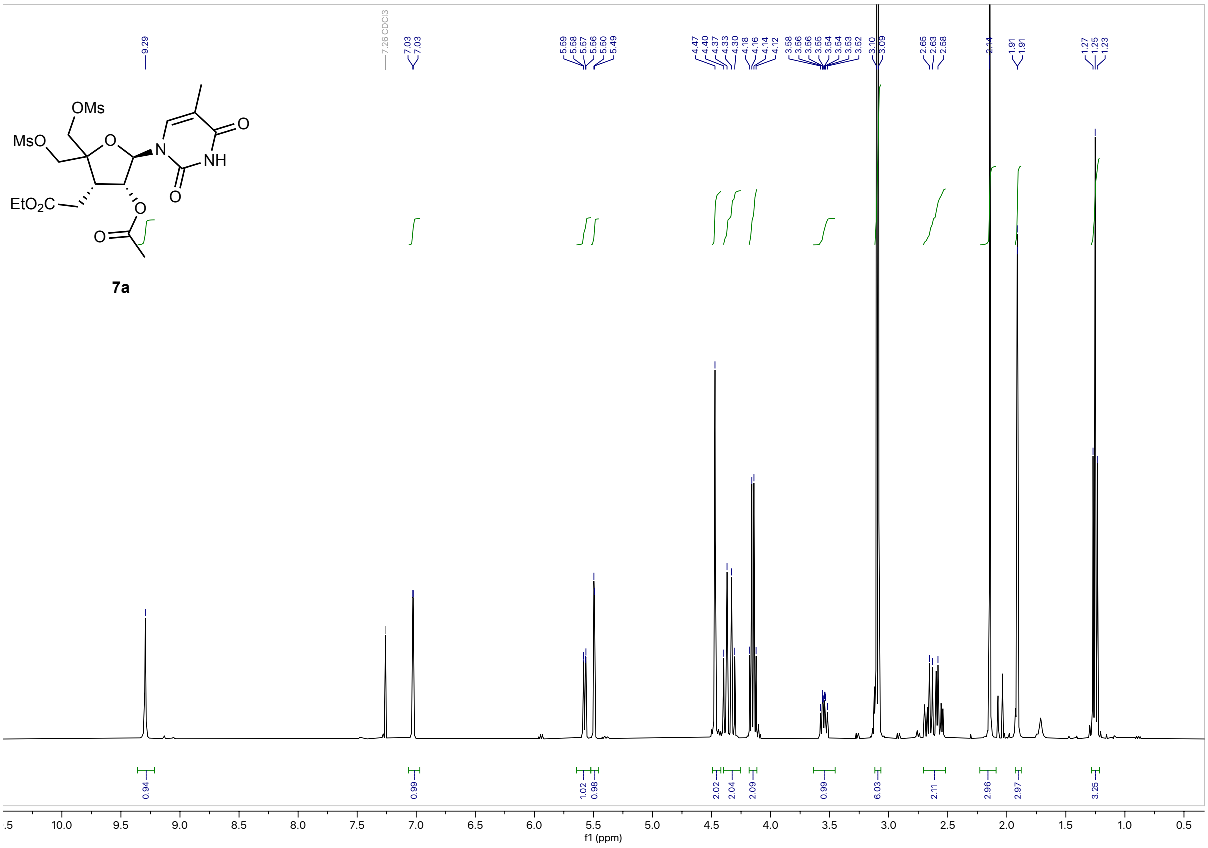

Supplementary Figure 53. ^{1}H NMR (400 MHz, $CDCl_3$) spectra of compound **7a**.

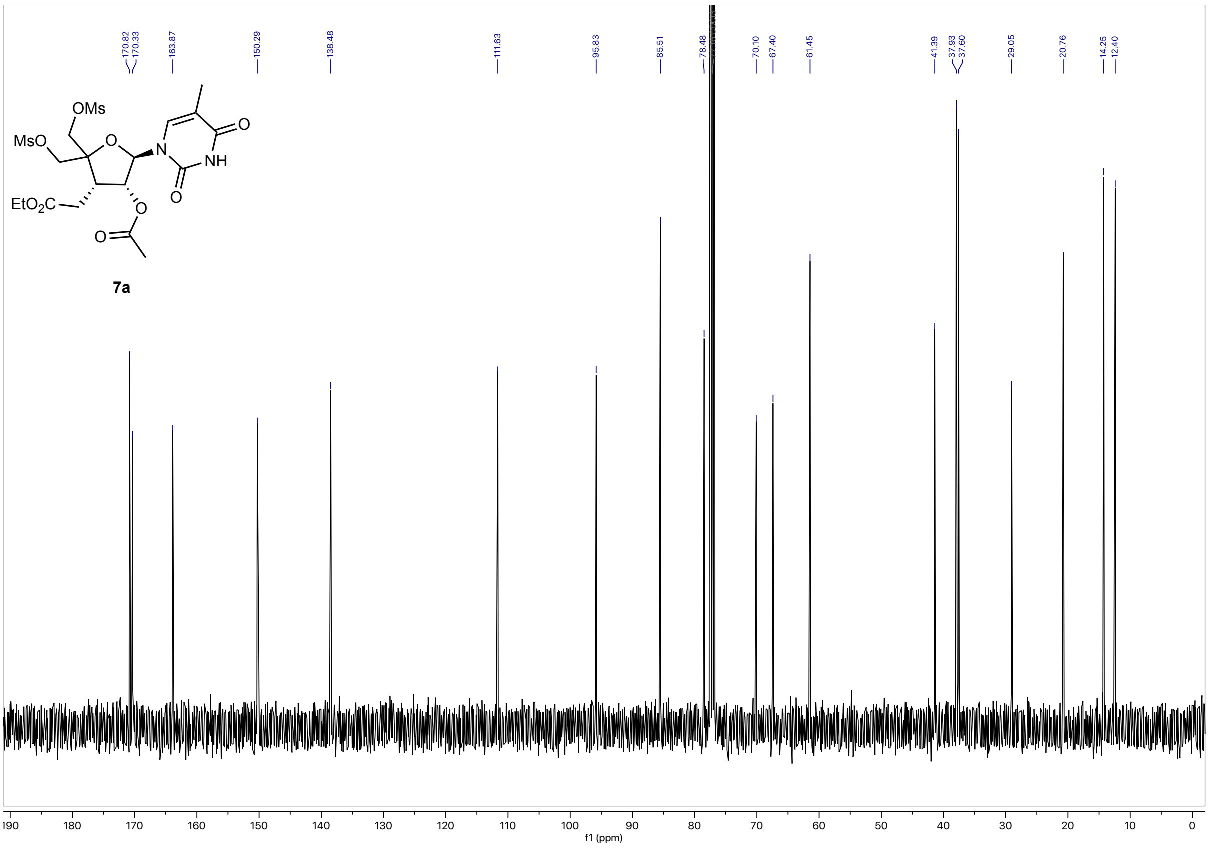

Supplementary Figure 54. ^{13}C NMR (101 MHz, $CDCl_3$) spectra of compound **7a**.

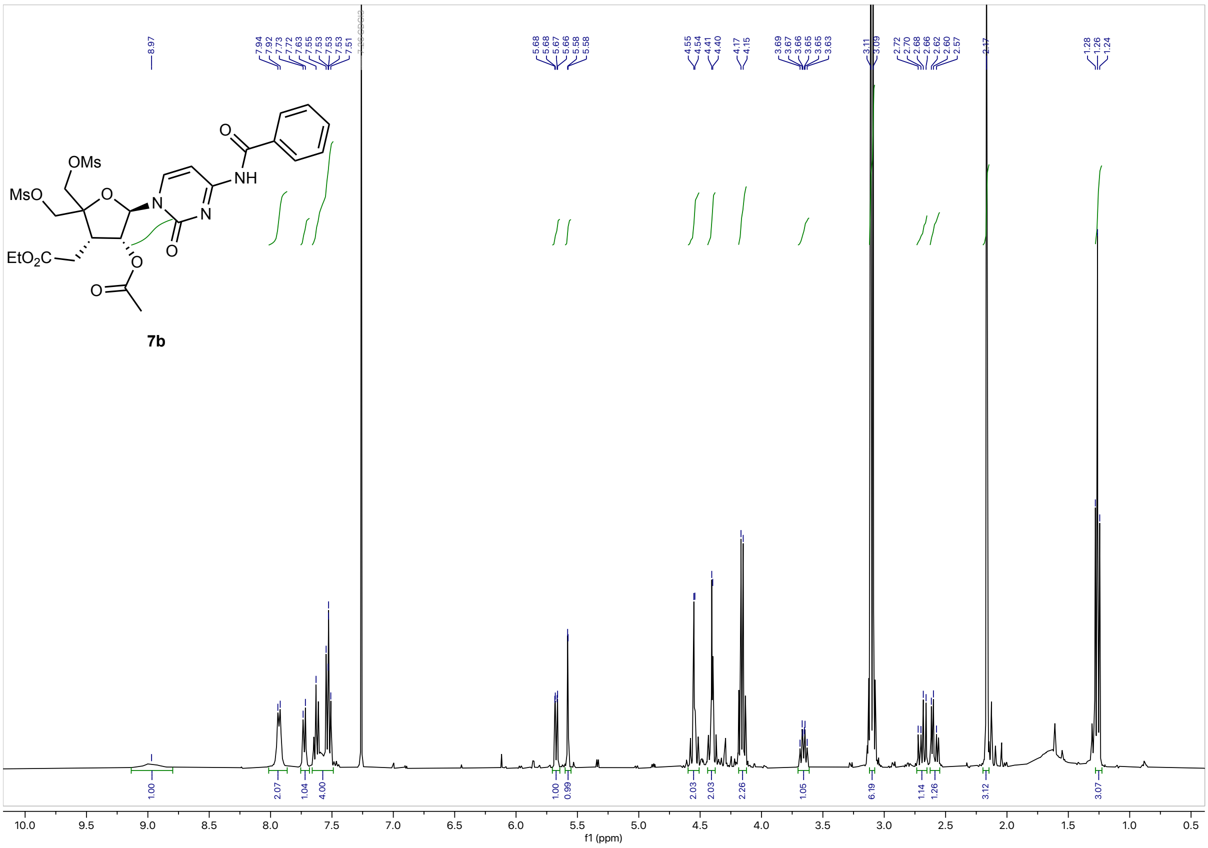

Supplementary Figure 55. ^{1}H NMR (400 MHz, $CDCl_3$) spectra of compound **7b**.

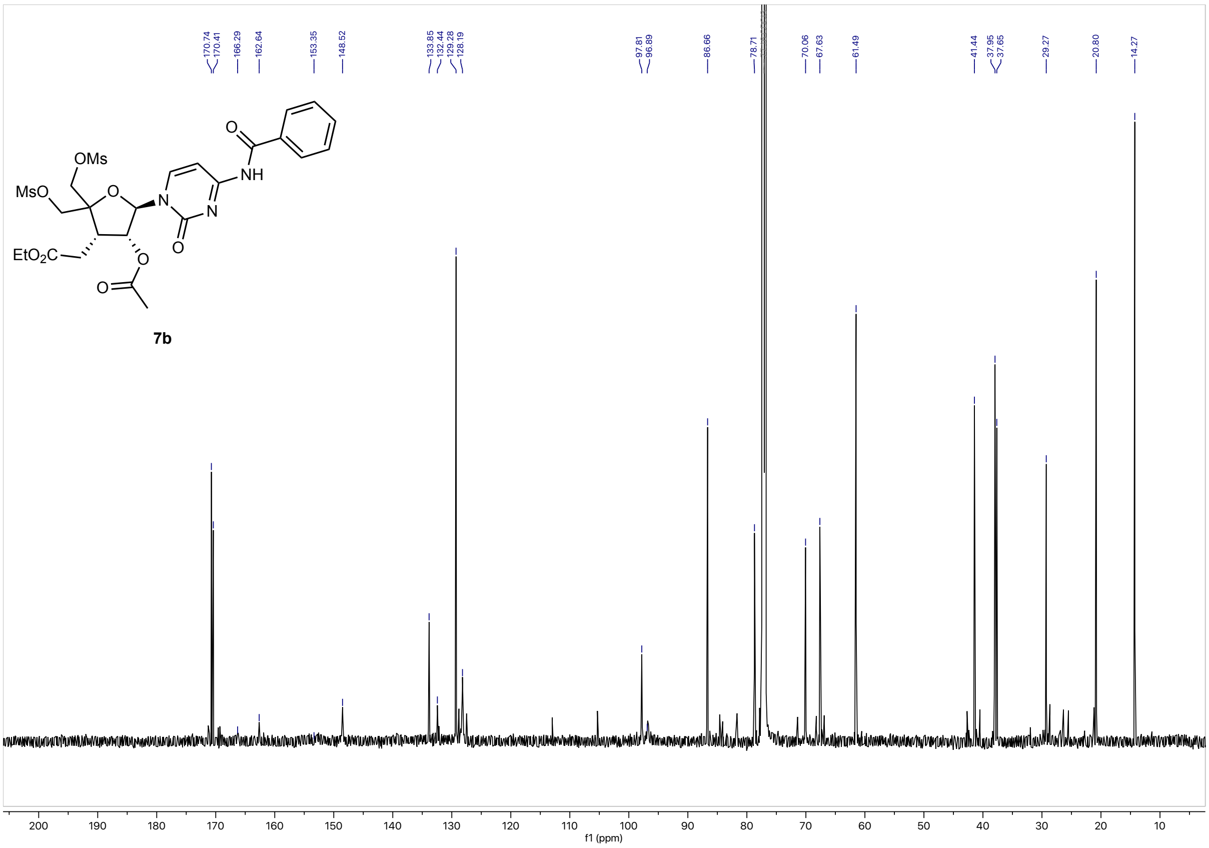

Supplementary Figure 56. ^{13}C NMR (151 MHz, $CDCl_3$) spectra of compound **7b**. Broad peaks observed at 166.3, 153.3, 96.9 ppm due to rotamers around the amide bond.

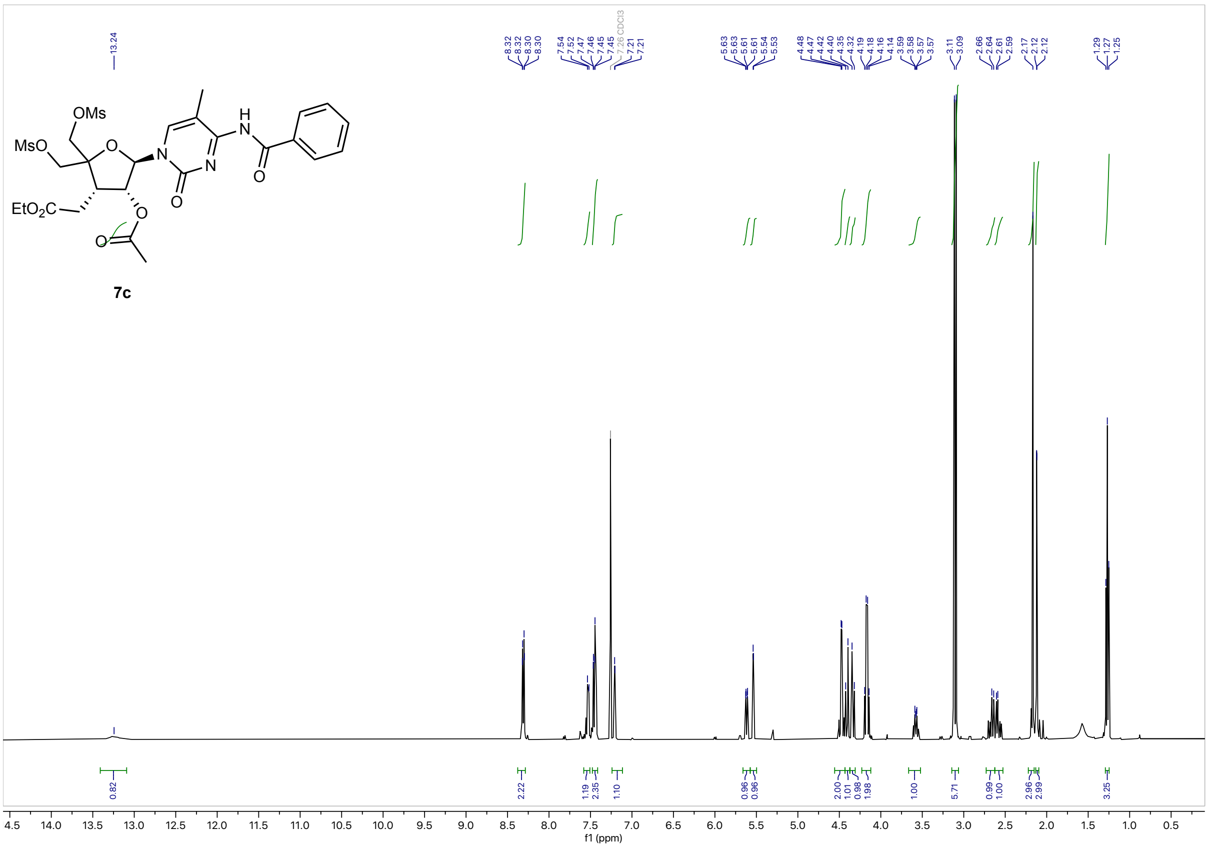

Supplementary Figure 57. ^{1}H NMR (400 MHz, $CDCl_3$) spectra of compound **7c**.

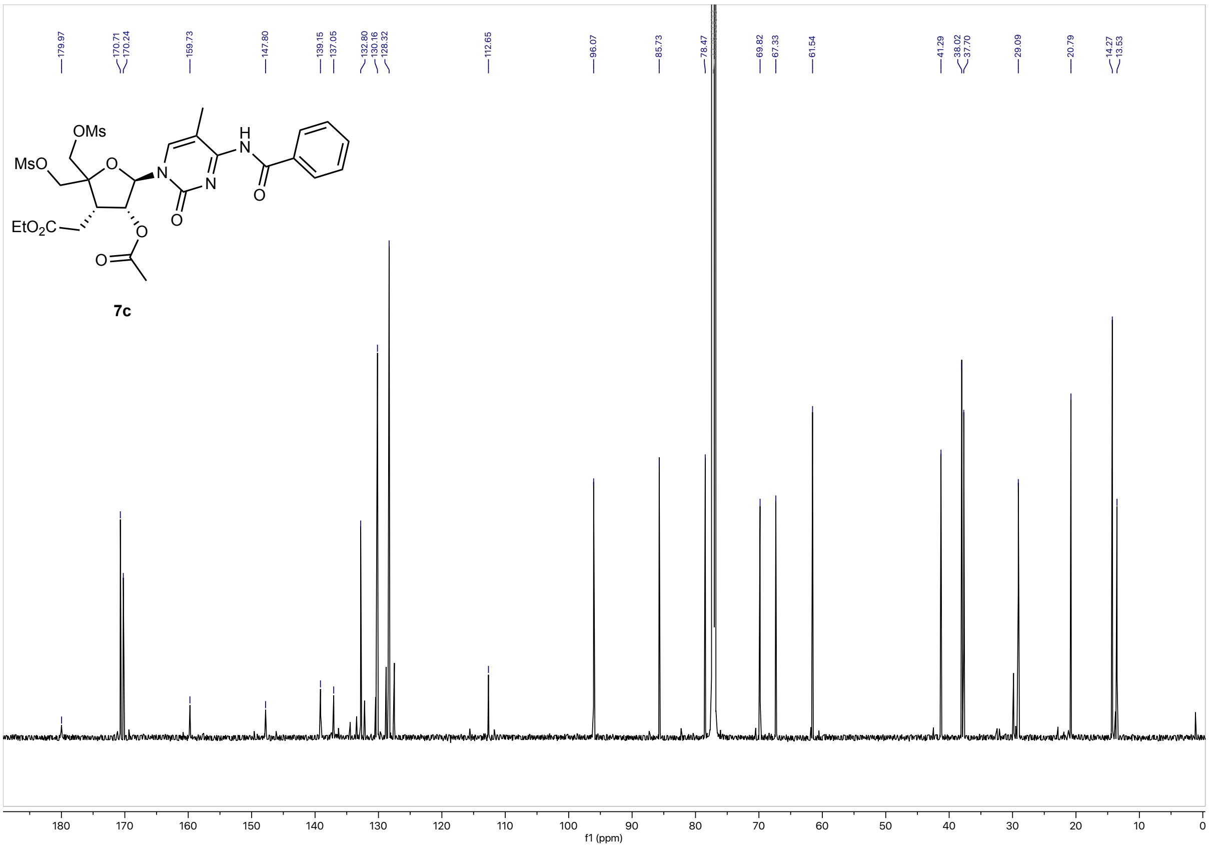

Supplementary Figure 58. ^{13}C NMR (101 MHz, $CDCl_3$) spectra of compound **7c**.

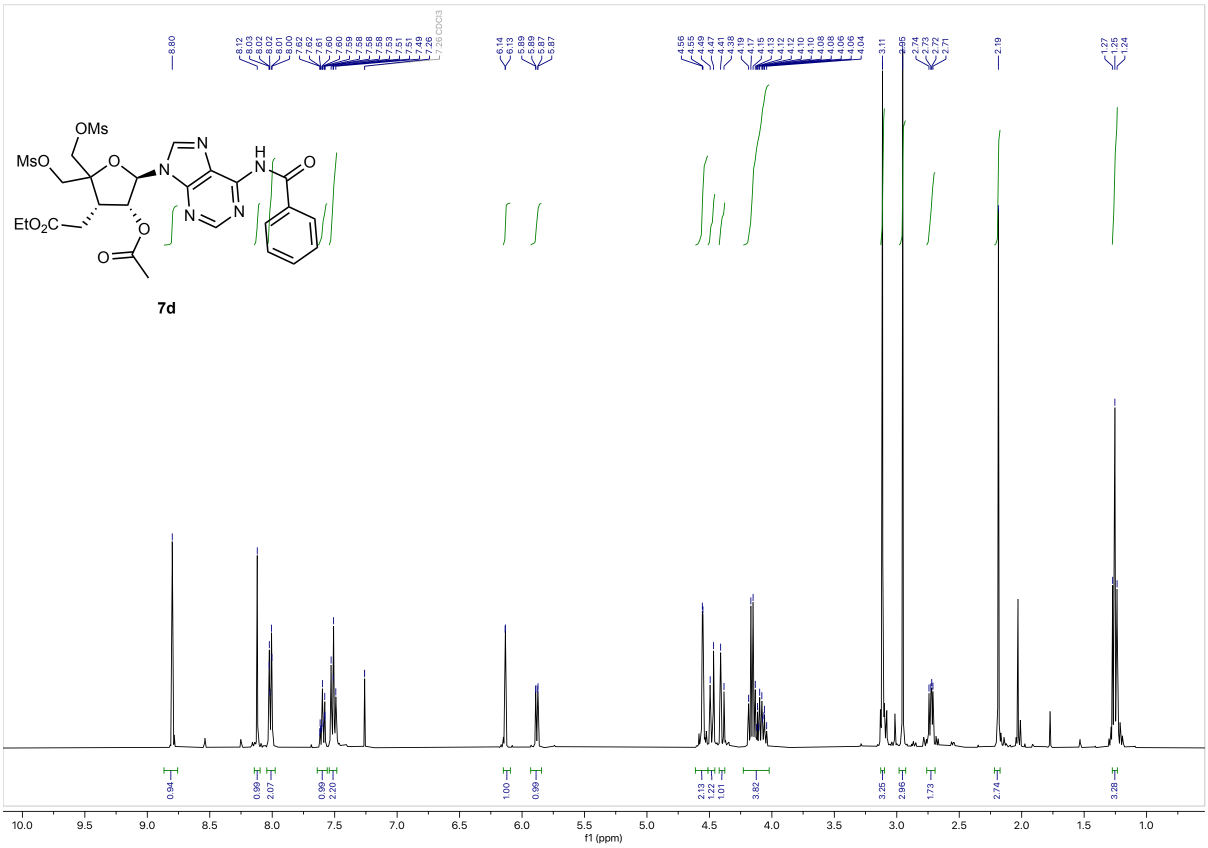

Supplementary Figure 59. ^{1}H NMR (400 MHz, $CDCl_3$) spectra of compound **7d**.

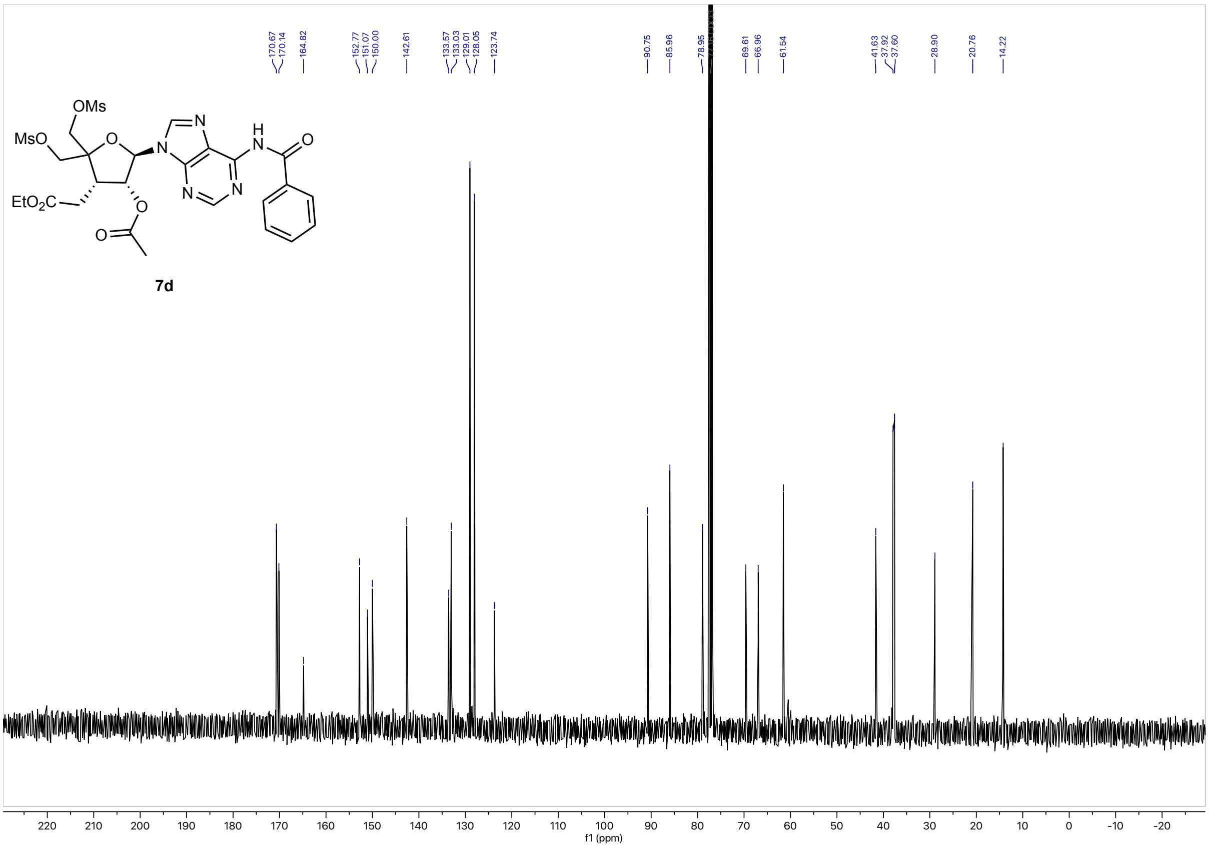

Supplementary Figure 60. ^{13}C NMR (101 MHz, $CDCl_3$) spectra of compound **7d**.

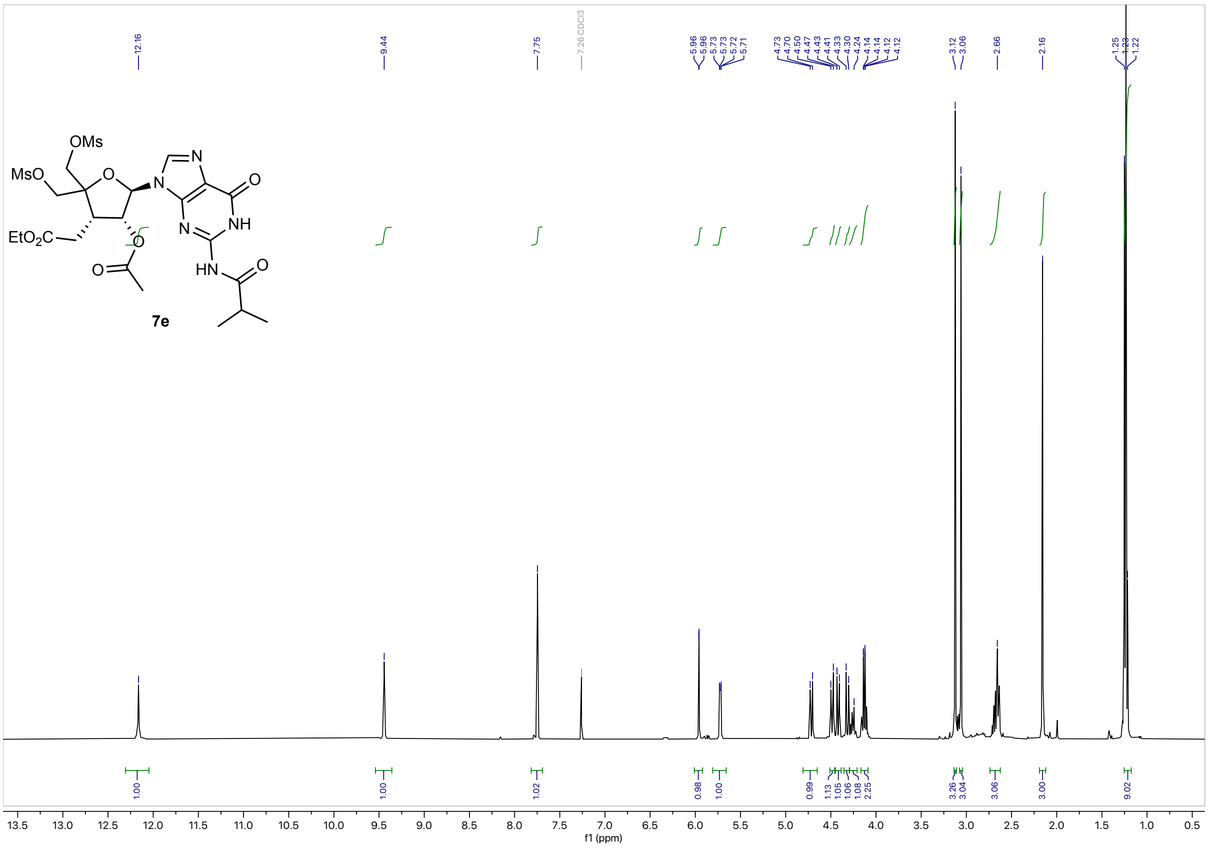

Supplementary Figure 61. ^{1}H NMR (400 MHz, $CDCl_3$) spectra of compound **7e**.

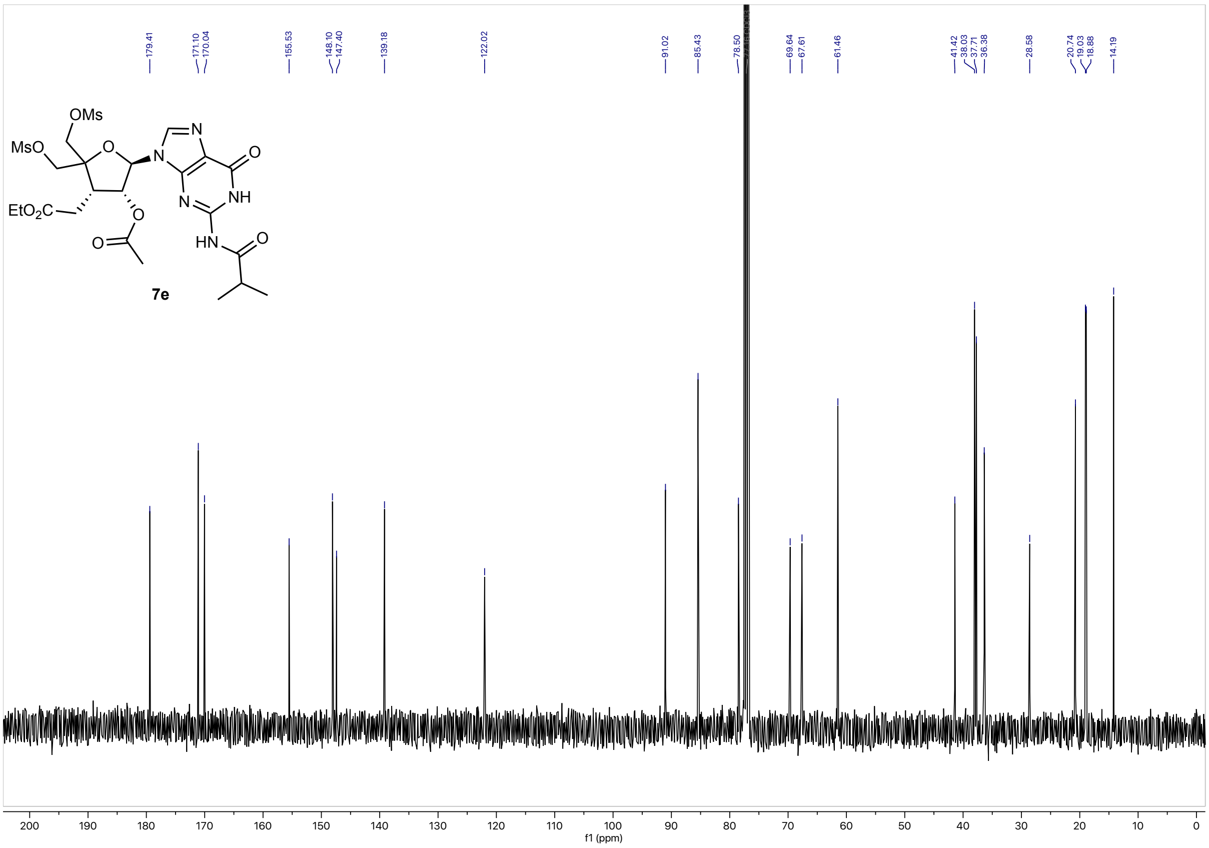

Supplementary Figure 62. ^{13}C NMR (101 MHz, $CDCl_3$) spectra of compound **7e**.

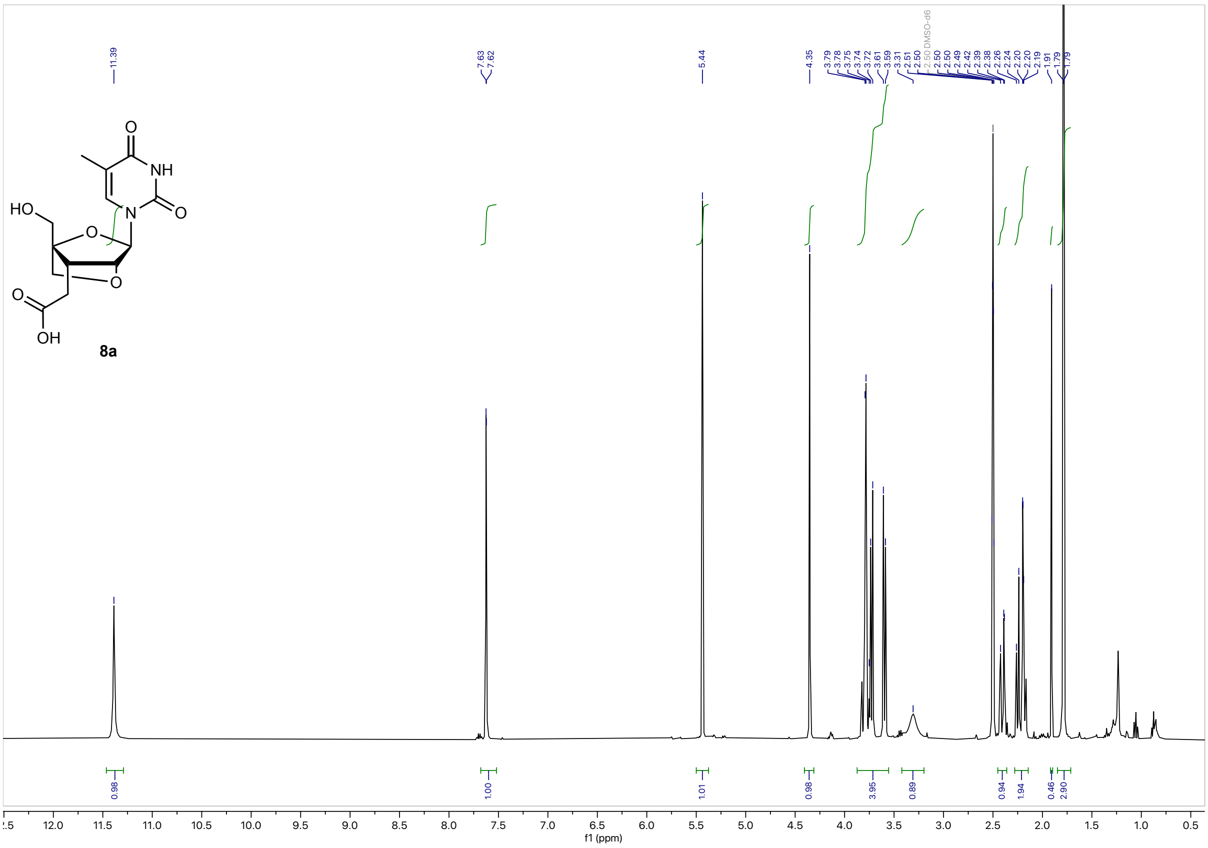

Supplementary Figure 63. ^{1}H NMR (400 MHz, d_6-DMSO) spectra of compound **8a** (crude).

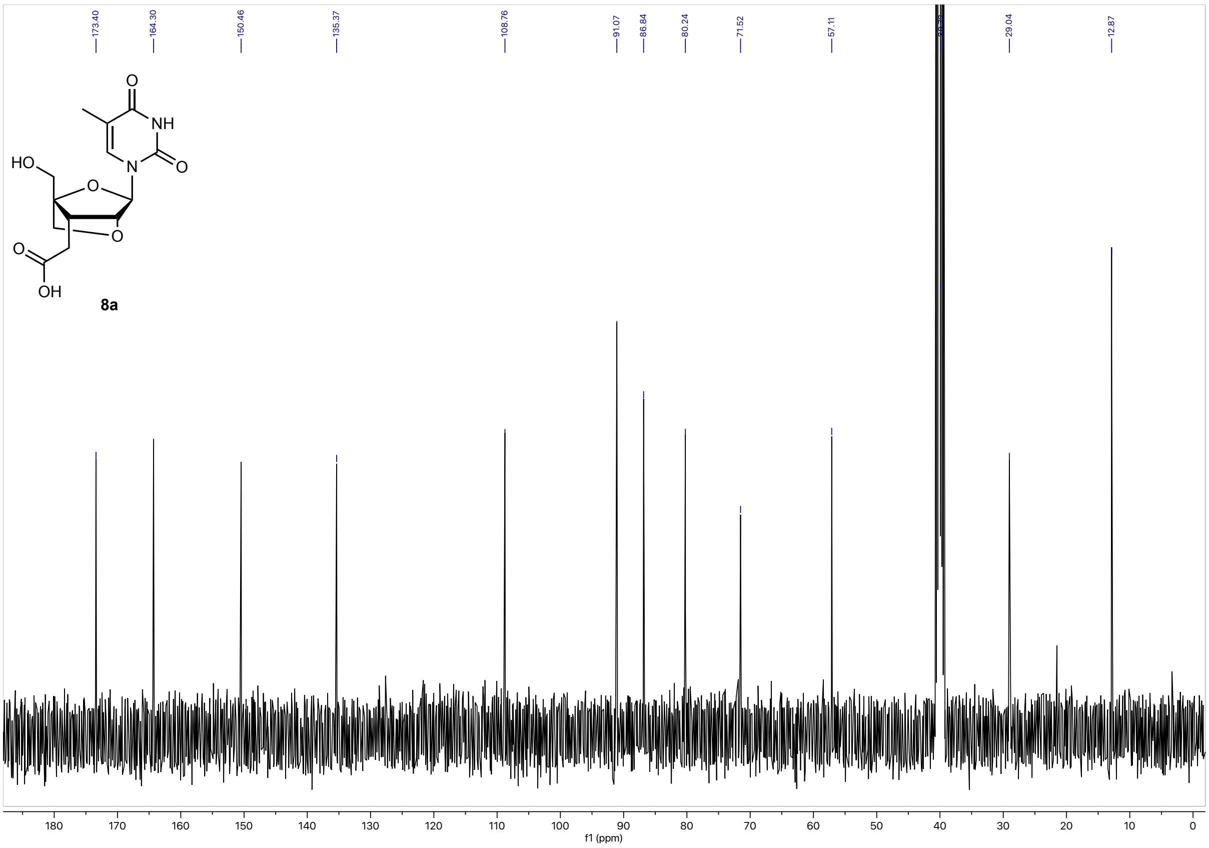

Supplementary Figure 64. ^{13}C NMR (101 MHz, d_6-DMSO) spectra of compound **8a** (crude). AcOH peak at 20 ppm.

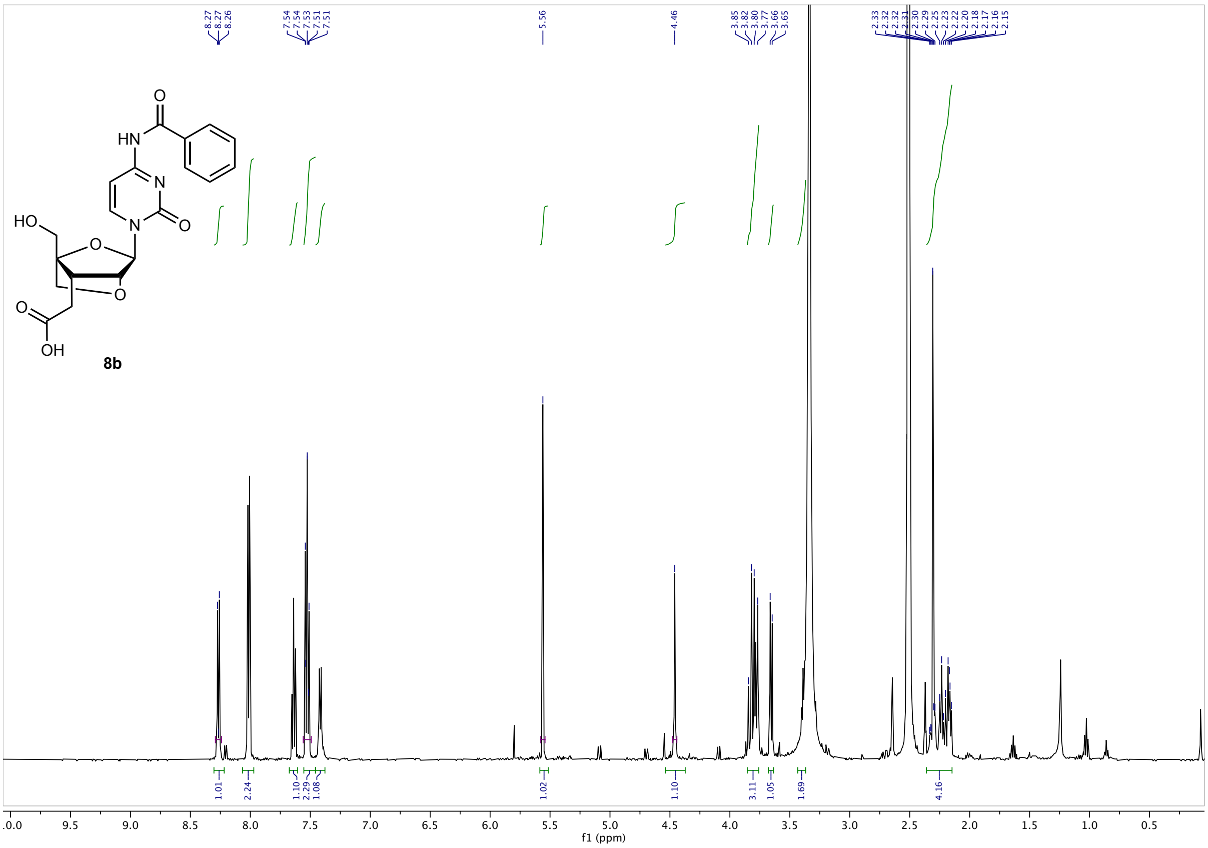

Supplementary Figure 65. ^{1}H NMR (500 MHz, d_6-DMSO) spectra of compound **8b** (crude).

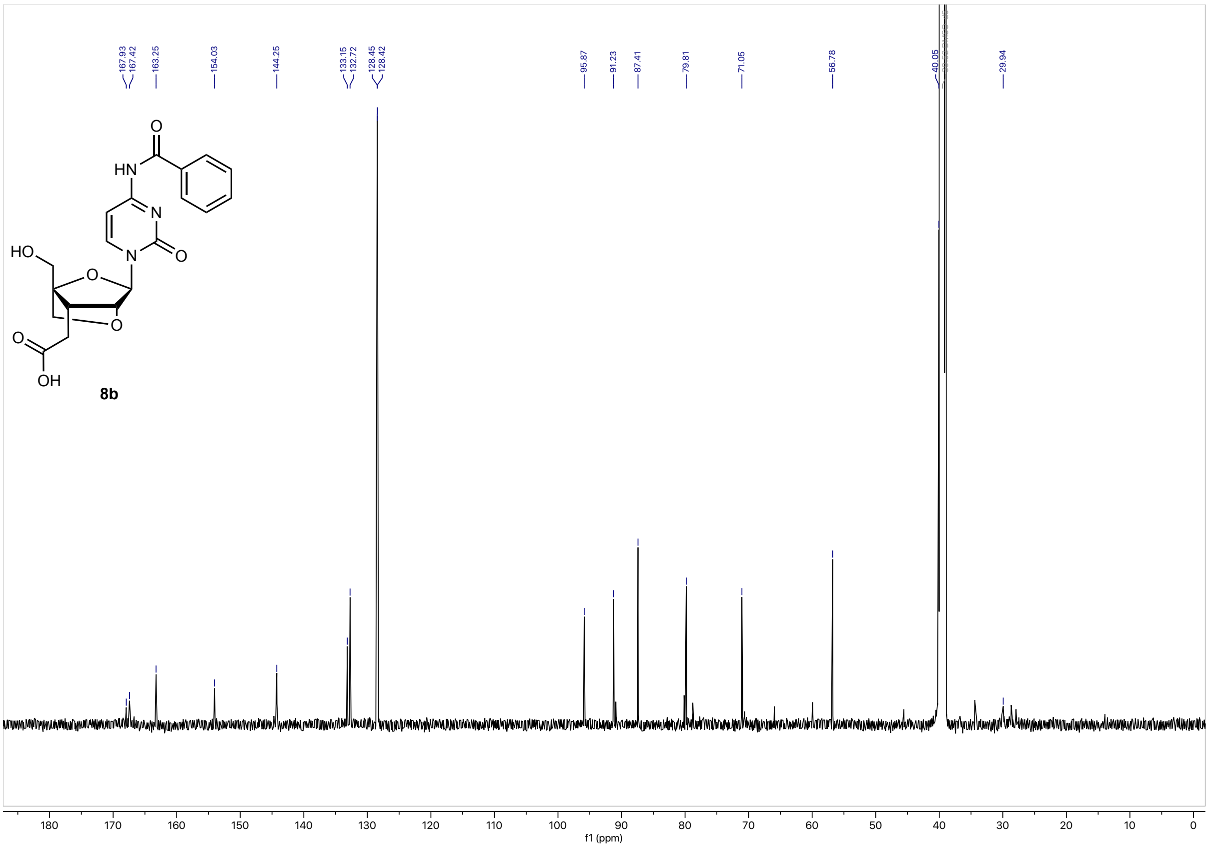

Supplementary Figure 66. ^{13}C NMR (500 MHz, d_6-DMSO) spectra of compound **8b** (crude).

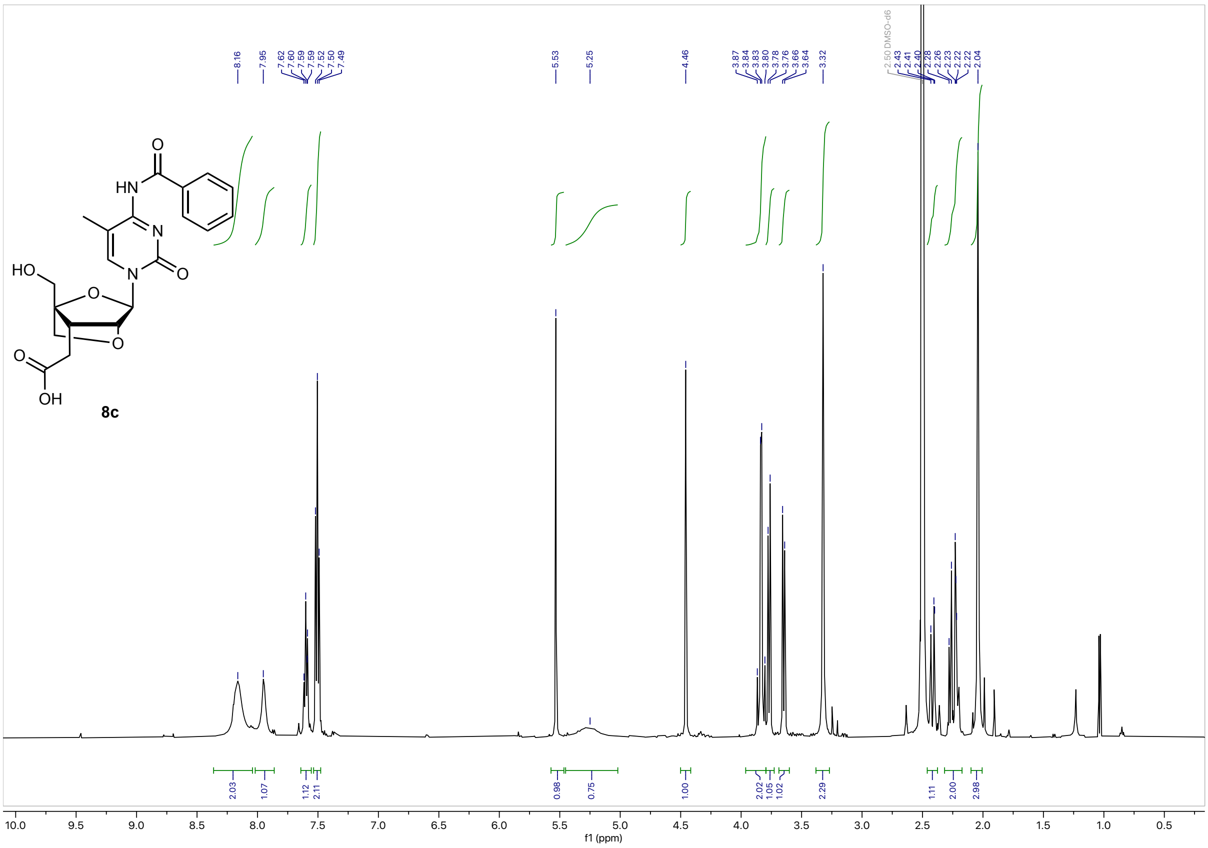

Supplementary Figure 67. ^{1}H NMR (500 MHz, d_6-DMSO) spectra of compound **8c** (crude). Residual iPrOH peak at 1.21 ppm.

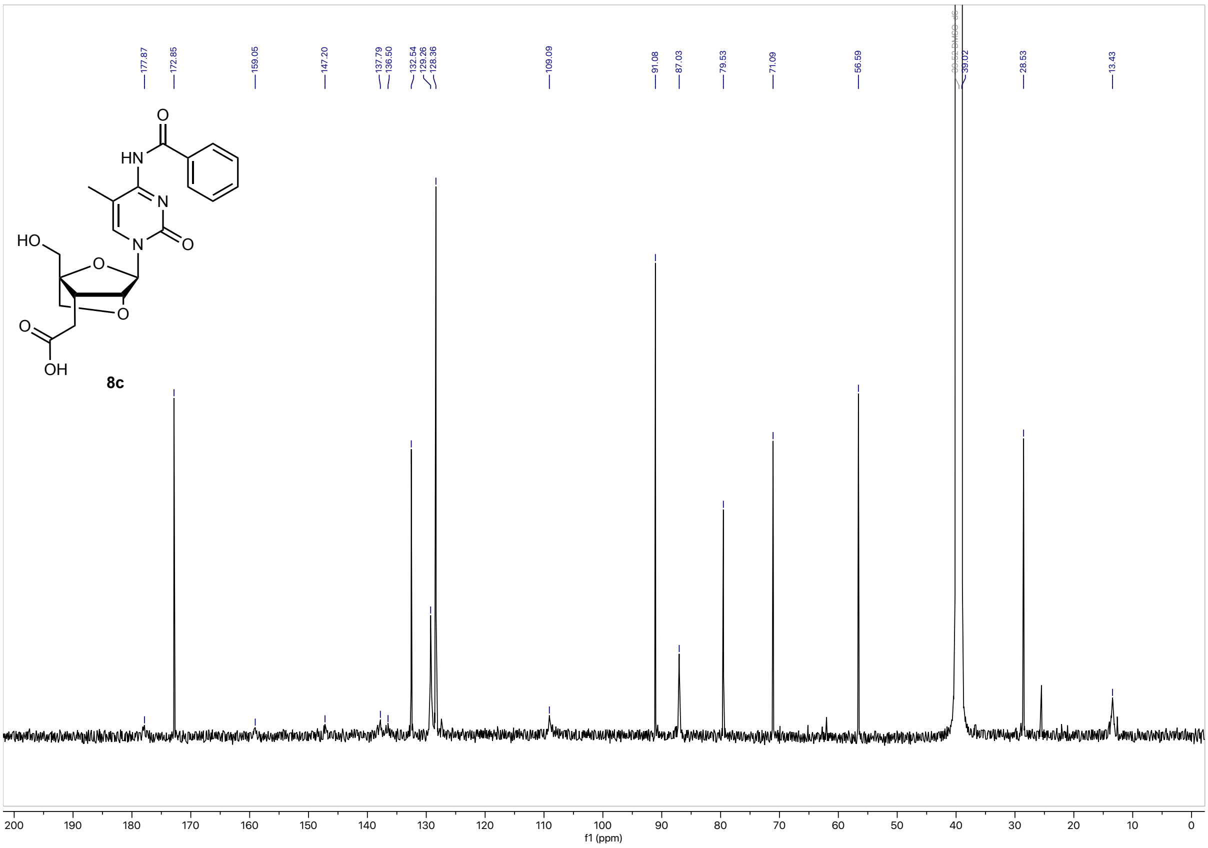

Supplementary Figure 68. ^{13}C NMR (151 MHz, d_6-DMSO) spectra of compound **8c** (crude). Broad peaks due to rotamers around amide. Residual iPrOH peak at 25.2 ppm.

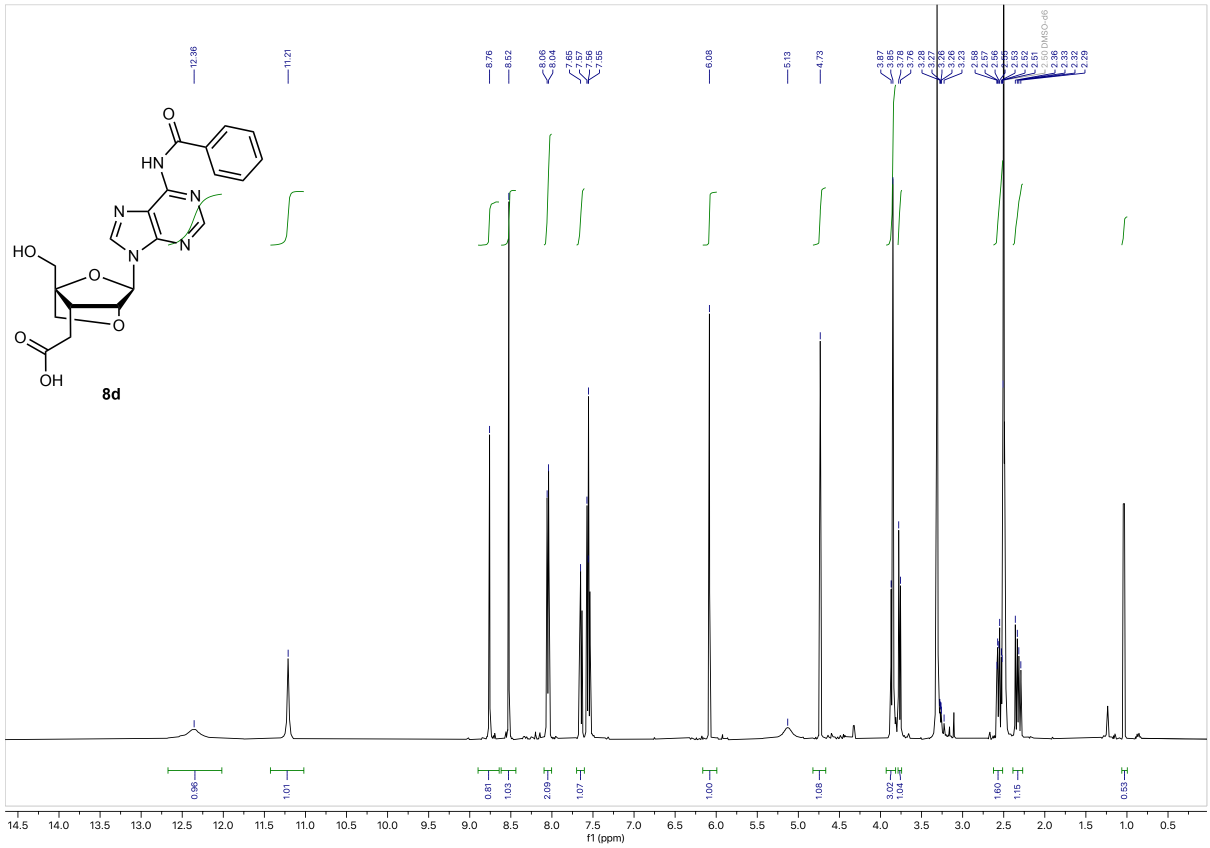

Supplementary Figure 69. ^{1}H NMR (400 MHz, d_6-DMSO) spectra of compound **8d** (crude). Residual iPrOH peak present.

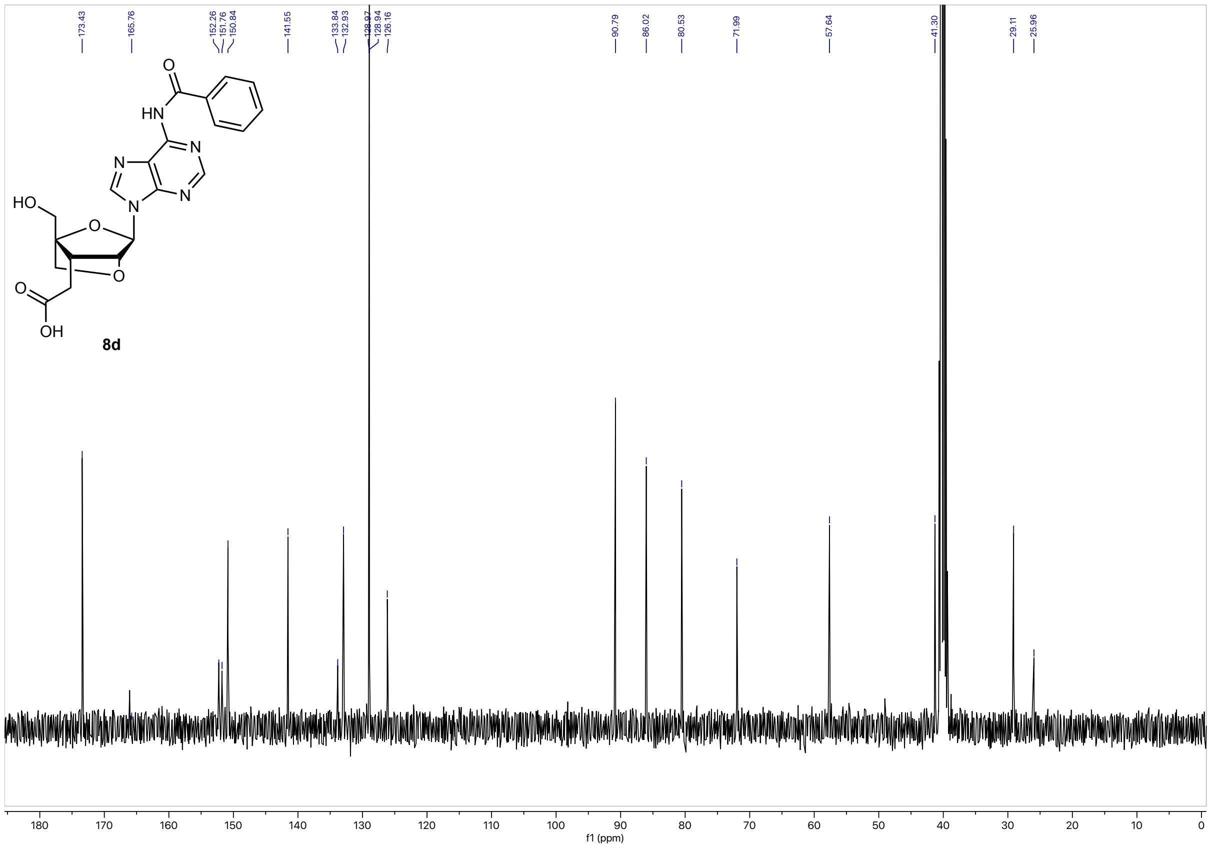

Supplementary Figure 70. ^{13}C NMR (101 MHz, d_6-DMSO) spectra of compound **8d** (crude). Residual iPrOH peak present.

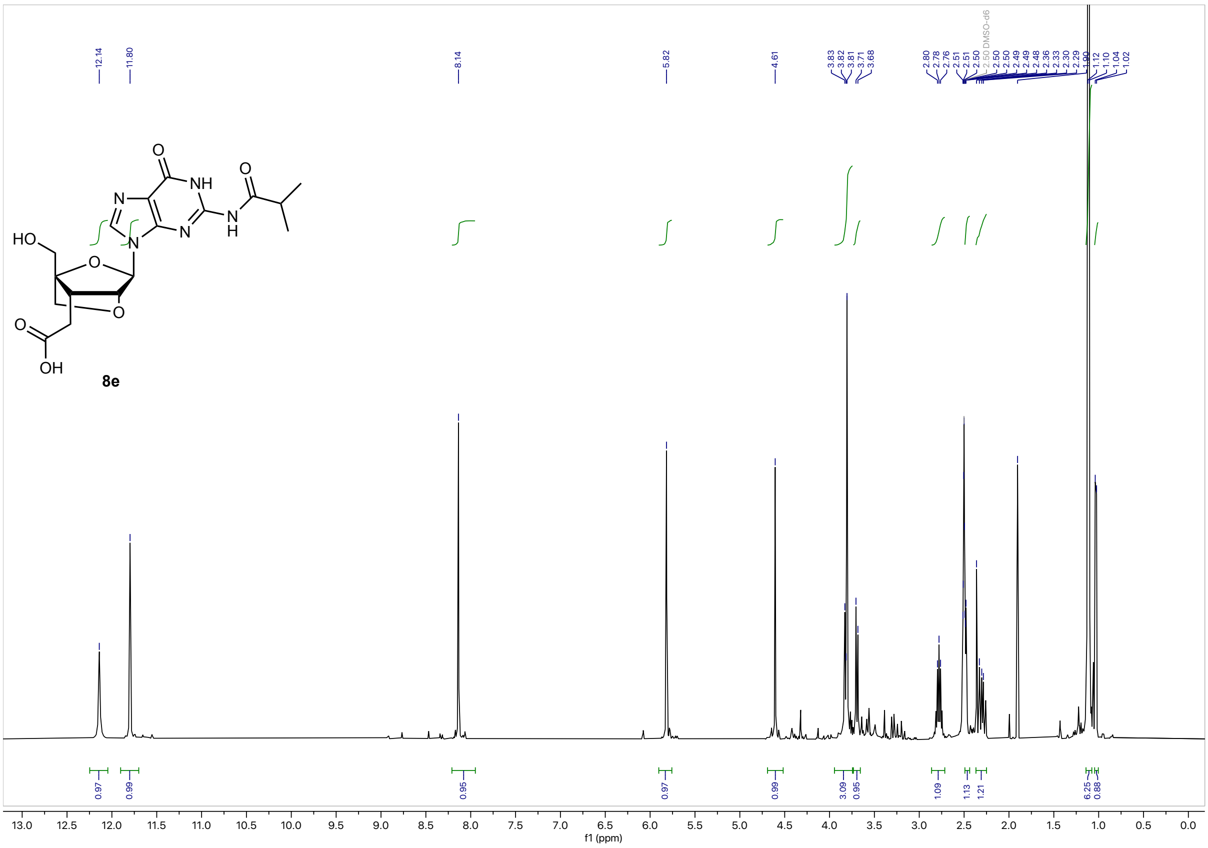

Supplementary Figure 71. ^{1}H NMR (400 MHz, d_6-DMSO) spectra of compound **8e** (crude). Residual iPrOH and AcOH.

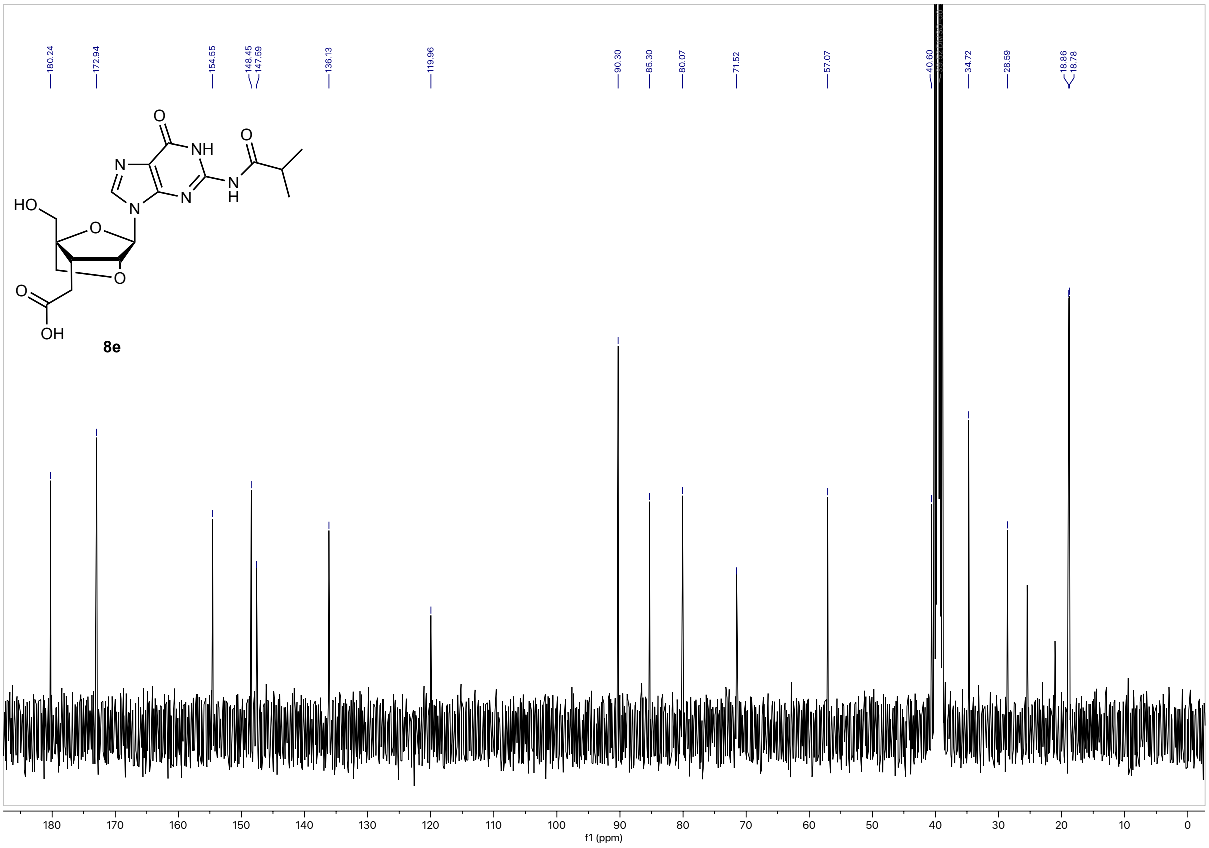

Supplementary Figure 72. ^{13}C NMR (101 MHz, d_6-DMSO) spectra of compound **8e** (crude). Residual iPrOH peak present.

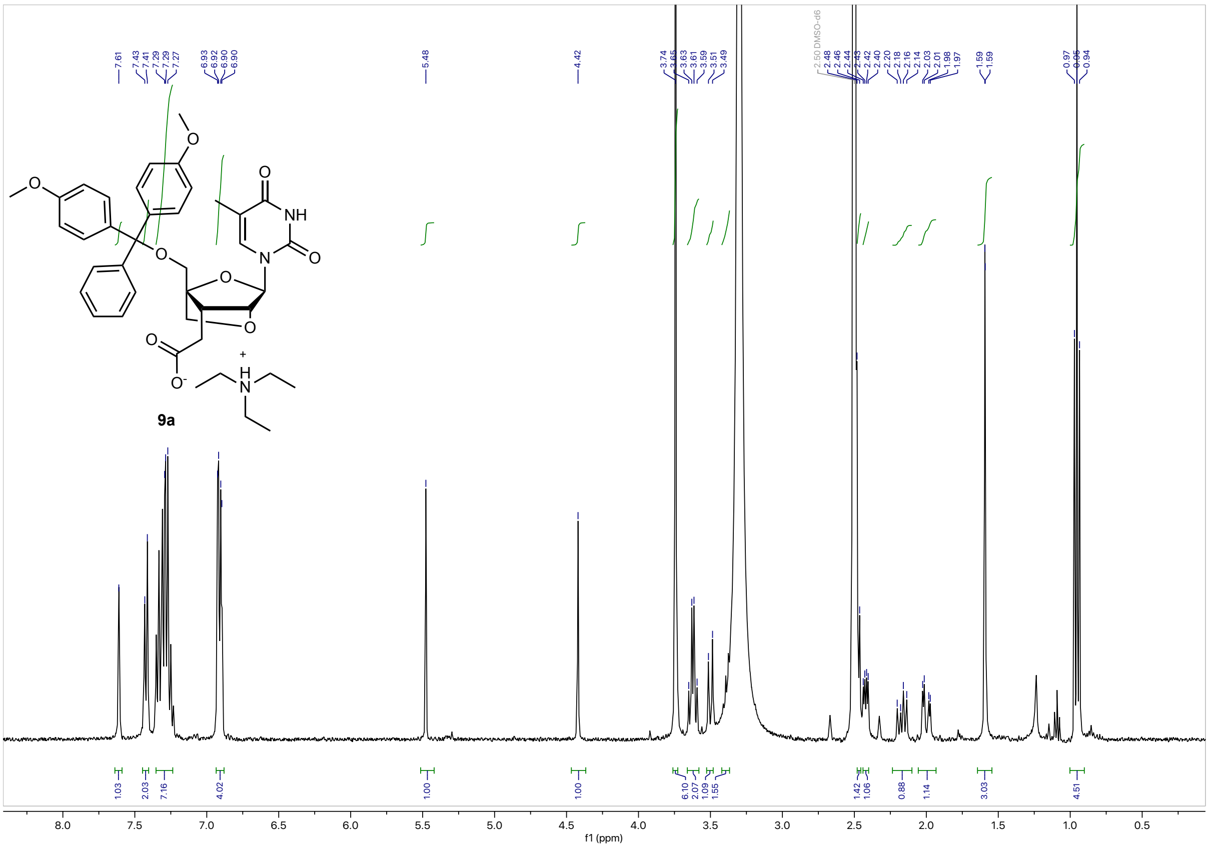

Supplementary Figure 73. ^{1}H NMR (400 MHz, d_6-DMSO) spectra of compound **9a**.

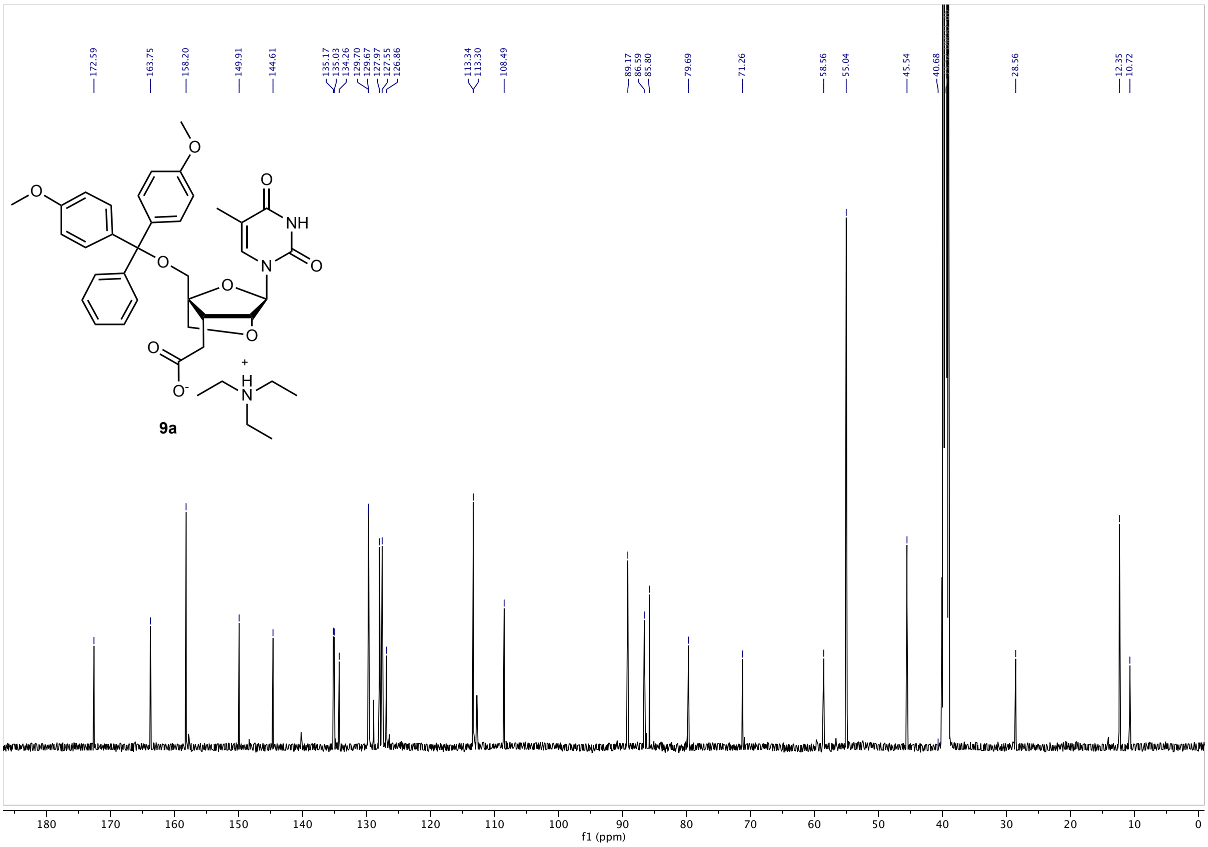

Supplementary Figure 74. ^{13}C NMR (151 MHz, d_6-DMSO) spectra of compound **9a**.

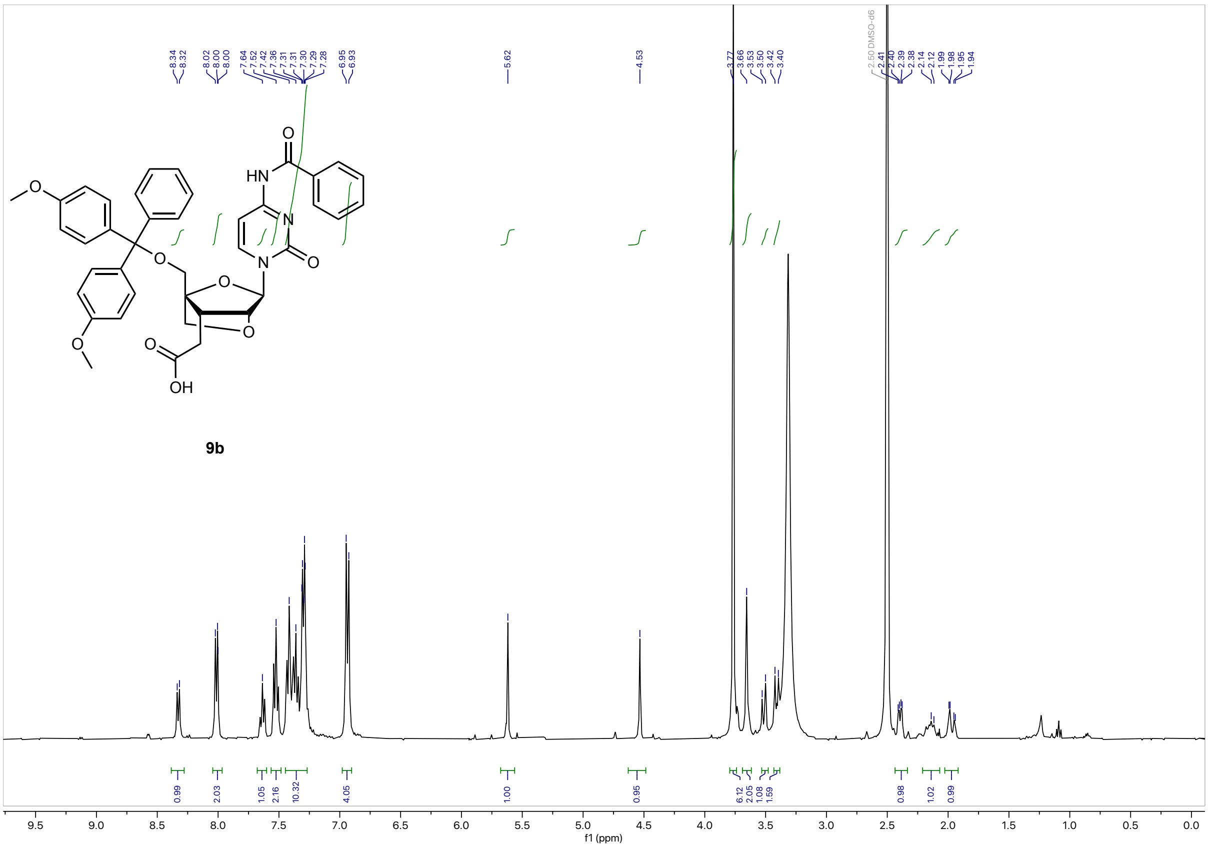

Supplementary Figure 75. ^{1}H NMR (400 MHz, d_6-DMSO) spectra of compound **9b**.

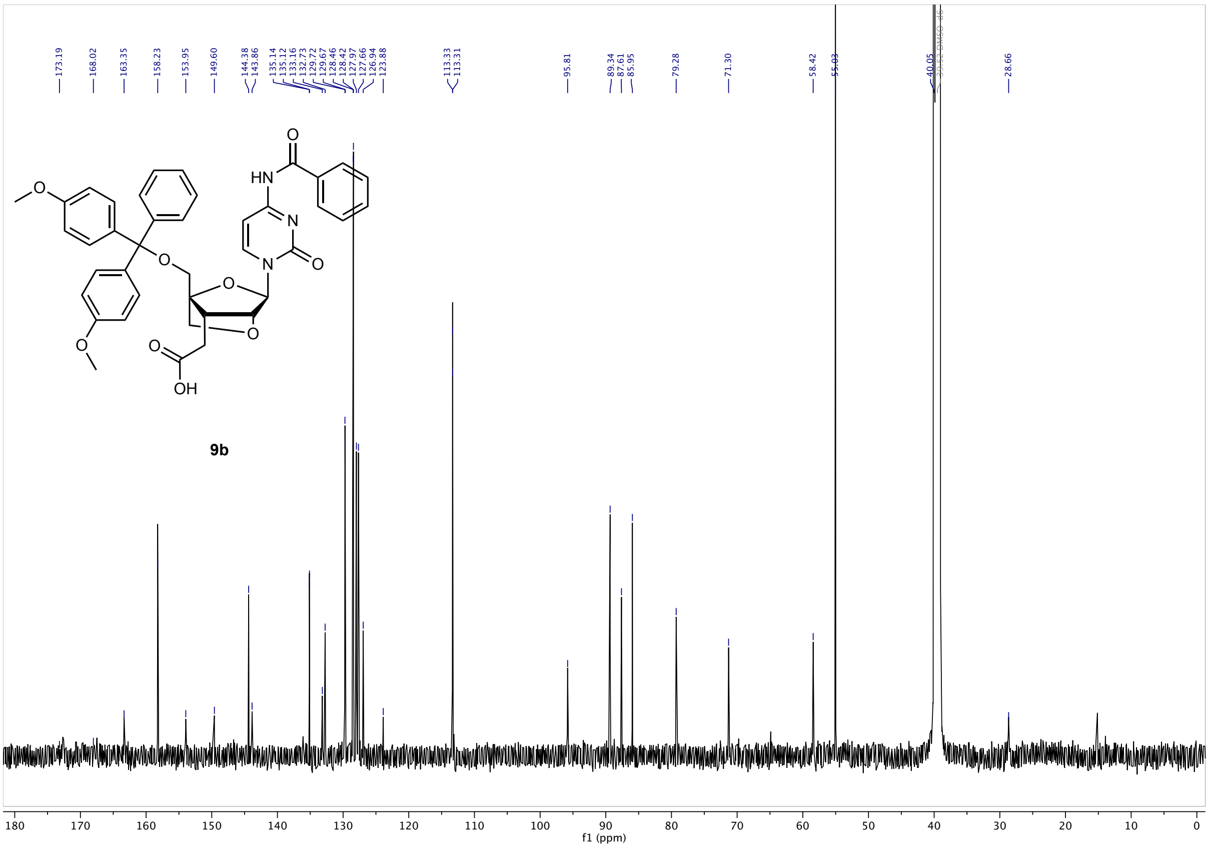

Supplementary Figure 76. ^{13}C NMR (126 MHz, d_6-DMSO) spectra of compound **9b**.

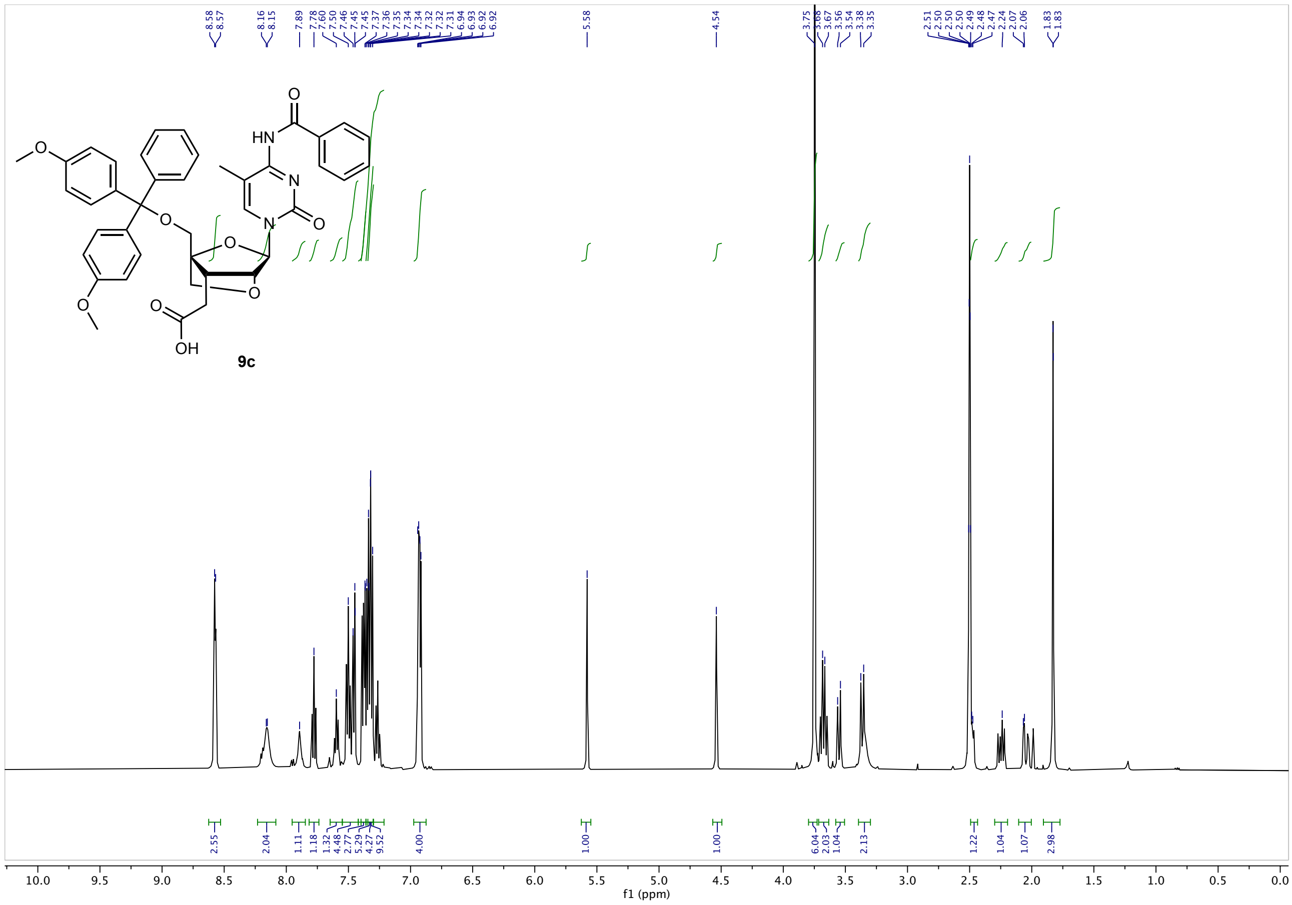

Supplementary Figure 77. ^{1}H NMR (500 MHz, d_6-DMSO) spectra of compound **9c**.

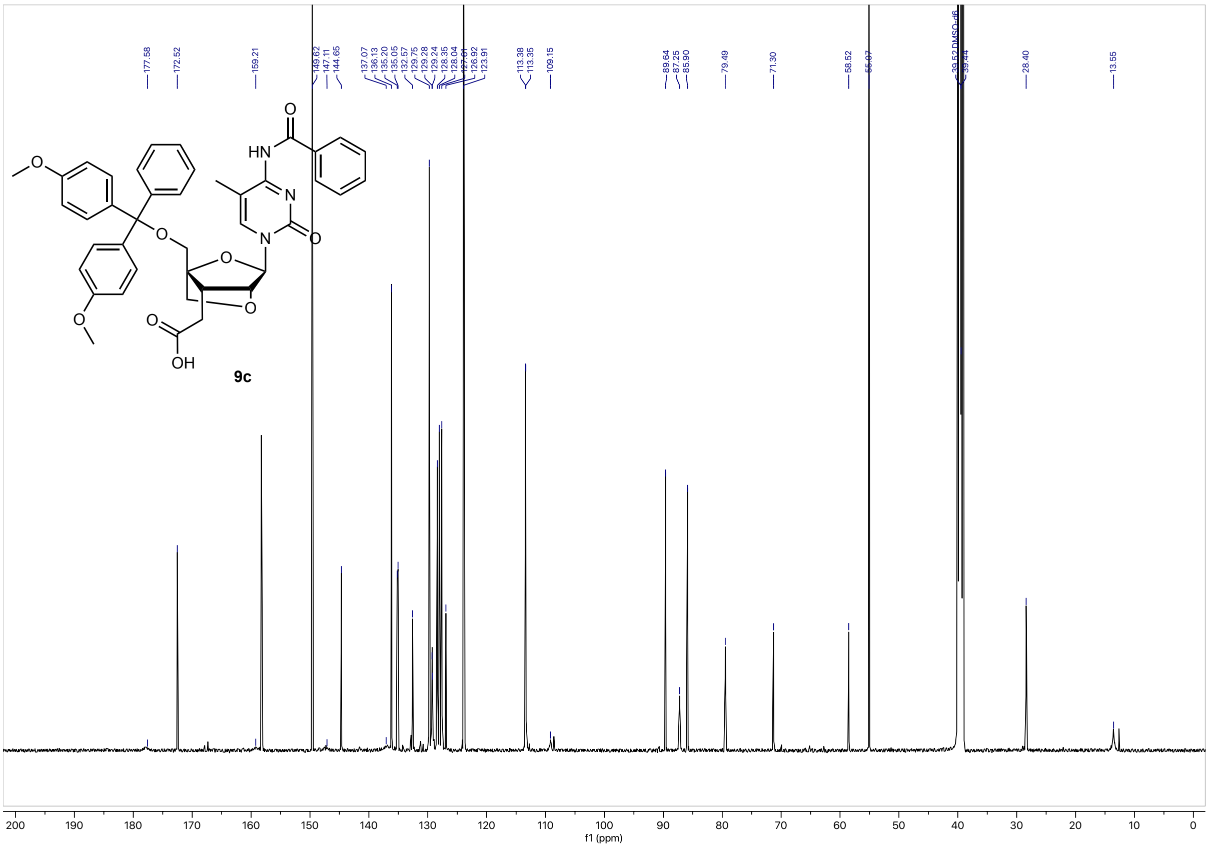

Supplementary Figure 78. ^{13}C NMR (126 MHz, d_6-DMSO) spectra of compound **9c**.

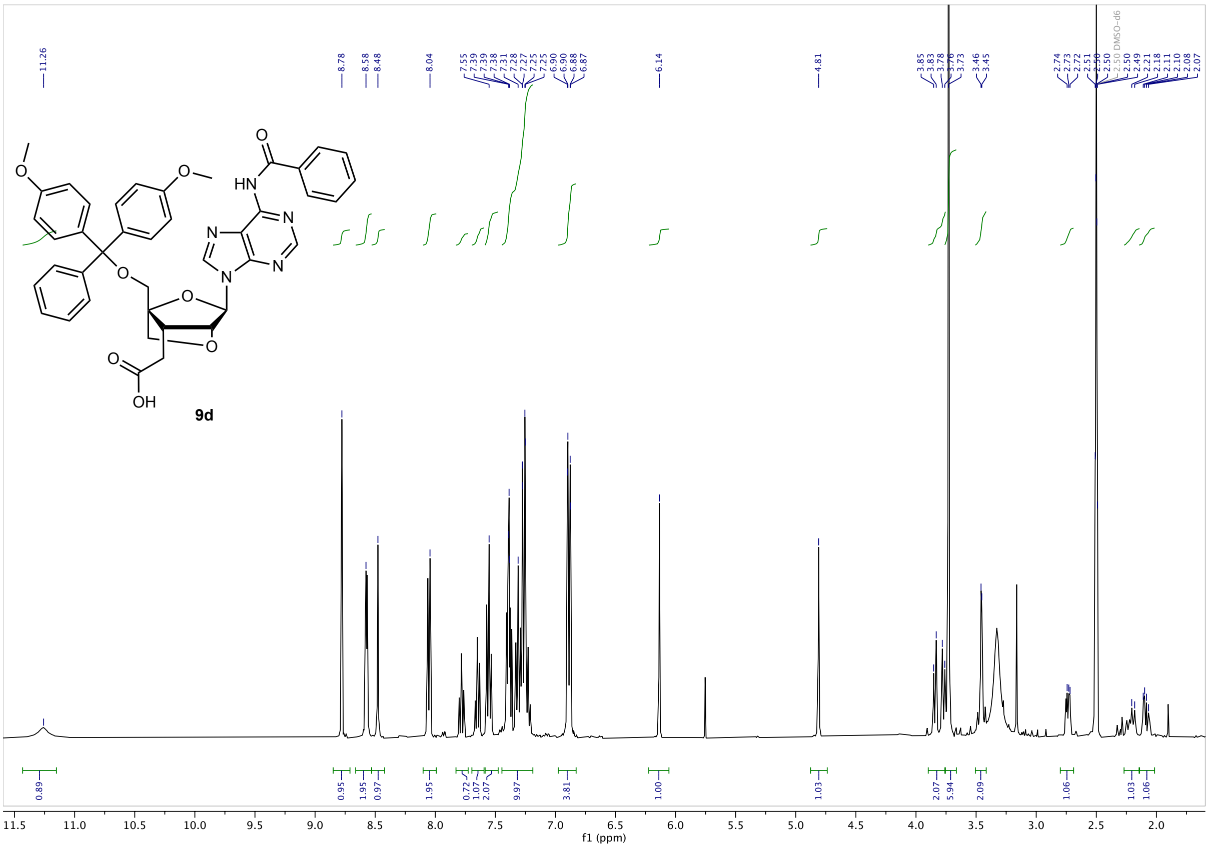

Supplementary Figure 79. ^{1}H NMR (400 MHz, d_6-DMSO) spectra of compound **9d**.

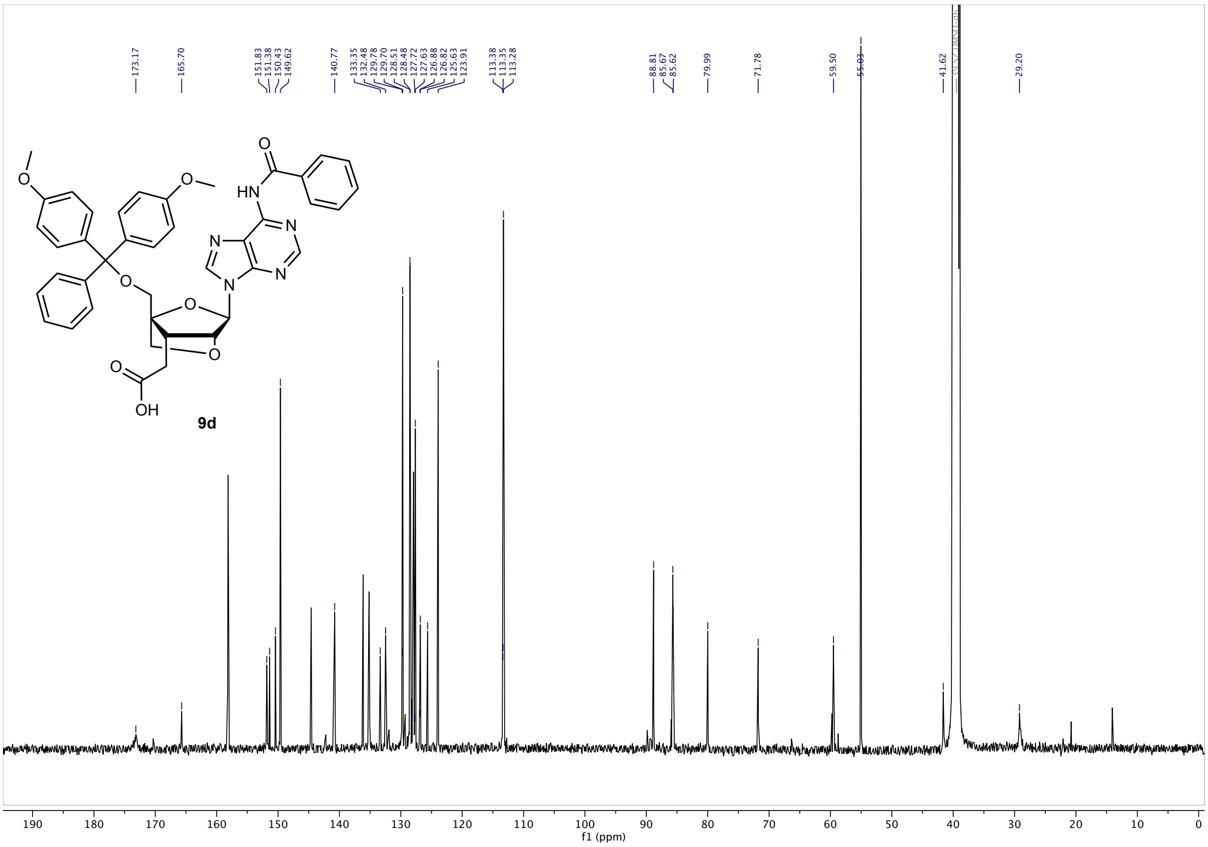

Supplementary Figure 80. ^{13}C NMR (101 MHz, d_6-DMSO) spectra of compound **9d**. Residual EtOAc at 14.4, 20.6 ppm (different sample to above NMR).

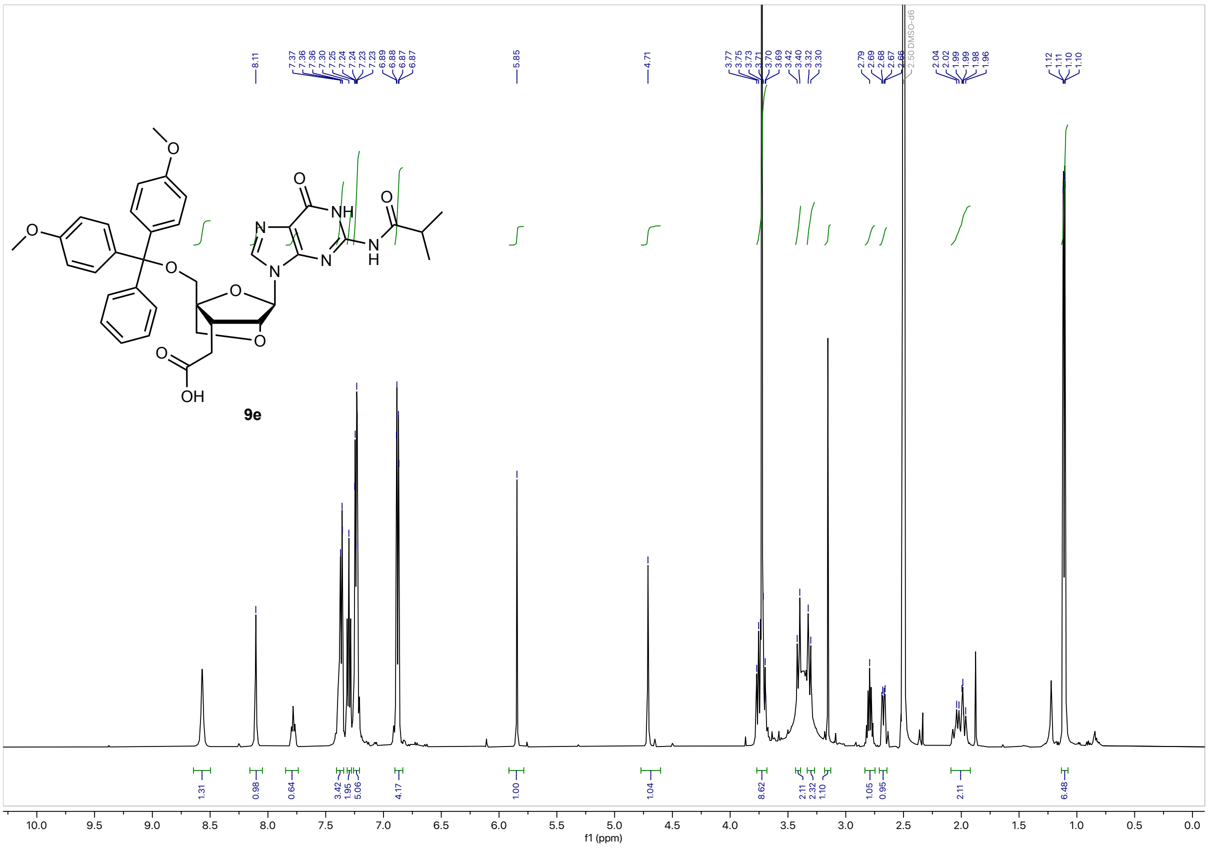

Supplementary Figure 81. ^{1}H NMR (500 MHz, d_6-DMSO) spectra of compound **9e**. Residual MeOH peak at 3.1 ppm.

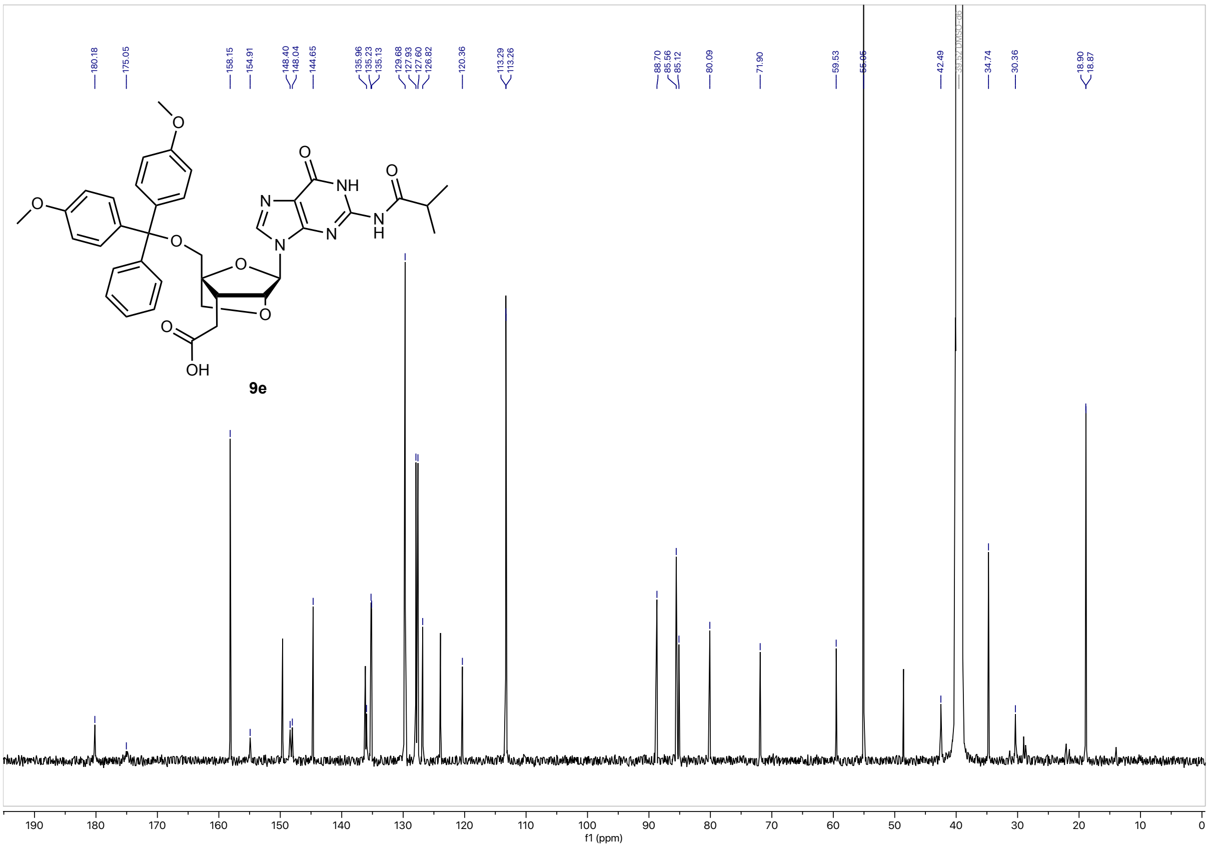

Supplementary Figure 82. ^{13}C NMR (126 MHz, d_6-DMSO) spectra of compound **9e**. Residual MeOH peak at 48.6 ppm.

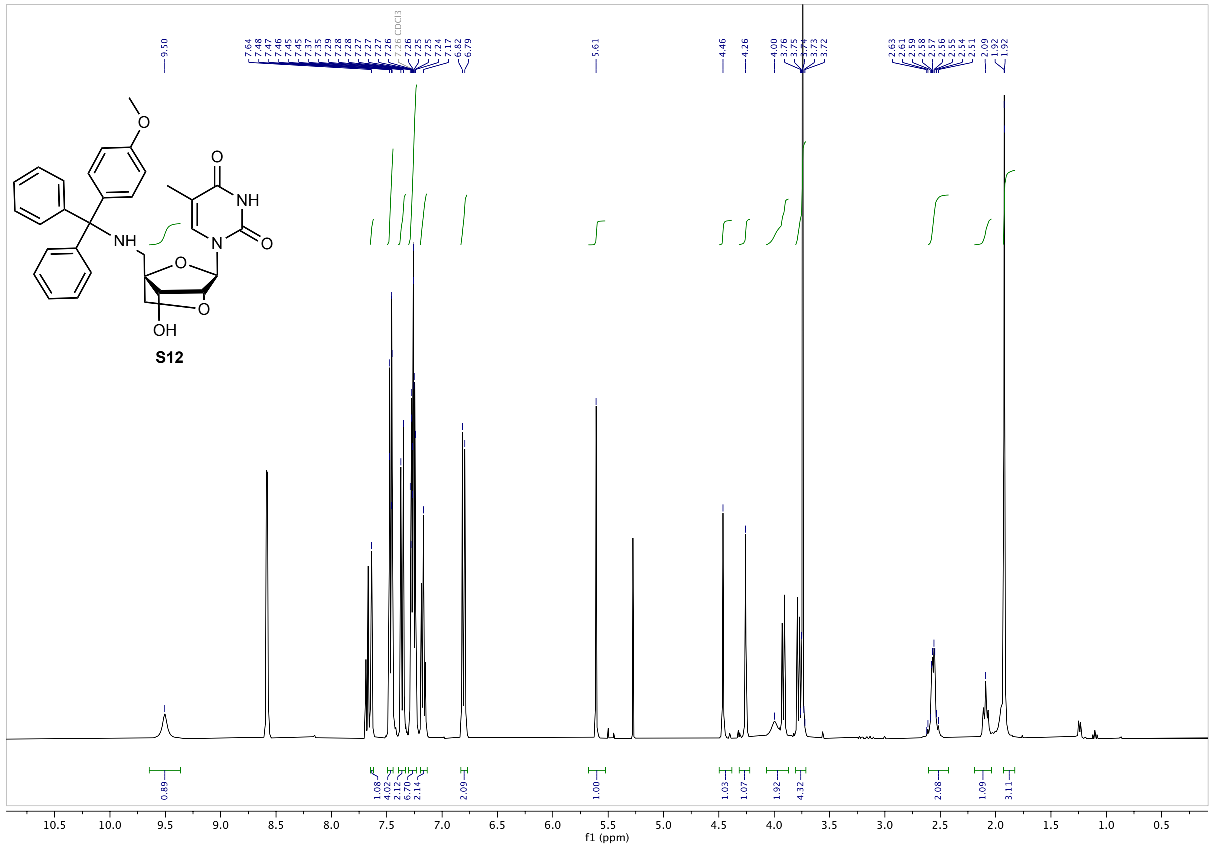

Supplementary Figure 83. ^{1}H NMR (400 MHz, $CDCl_3$) spectra of compound **S12**. Pyridine peaks at 8.62, 7.29, 7.68 ppm.

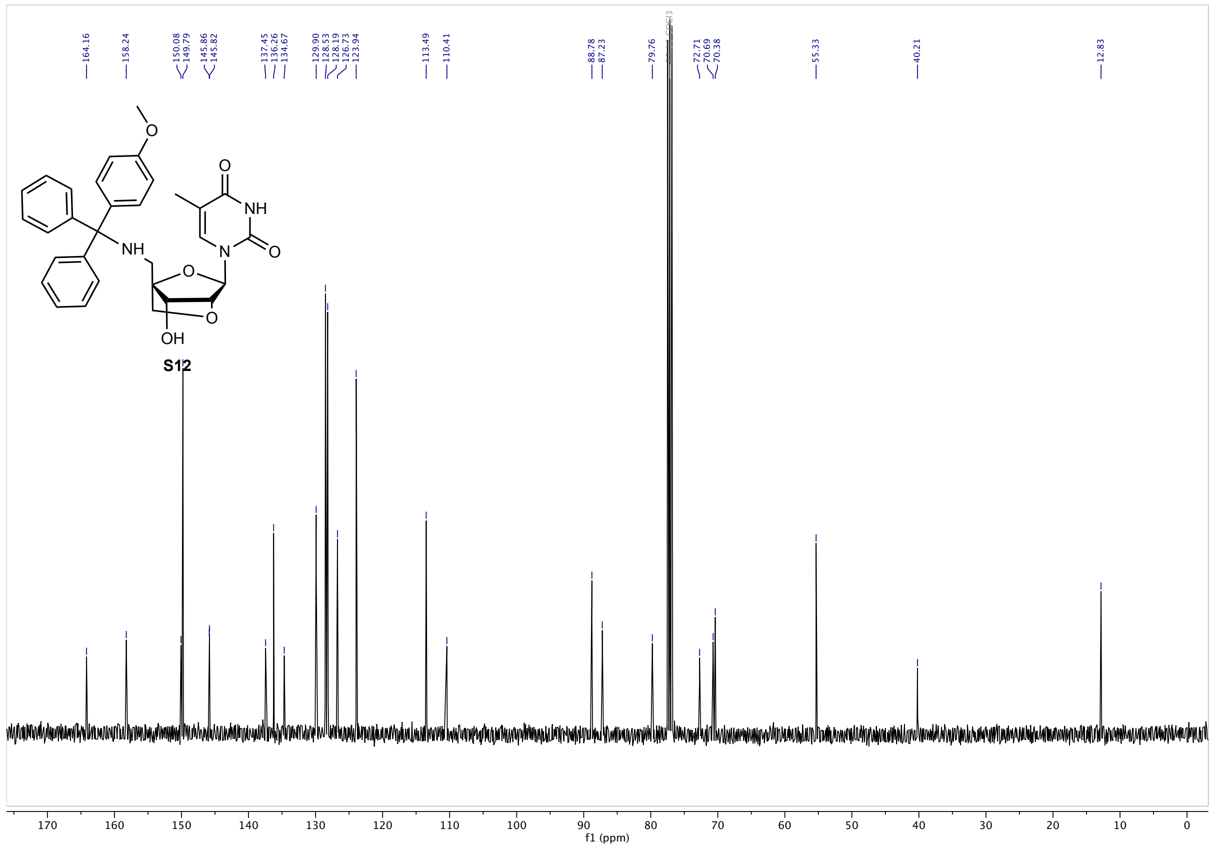

Supplementary Figure 84. ^{13}C NMR (121 MHz, $CDCl_3$) spectra of compound **S12**. Pyridine peaks at 149.8, 123.9, 136.3 ppm.

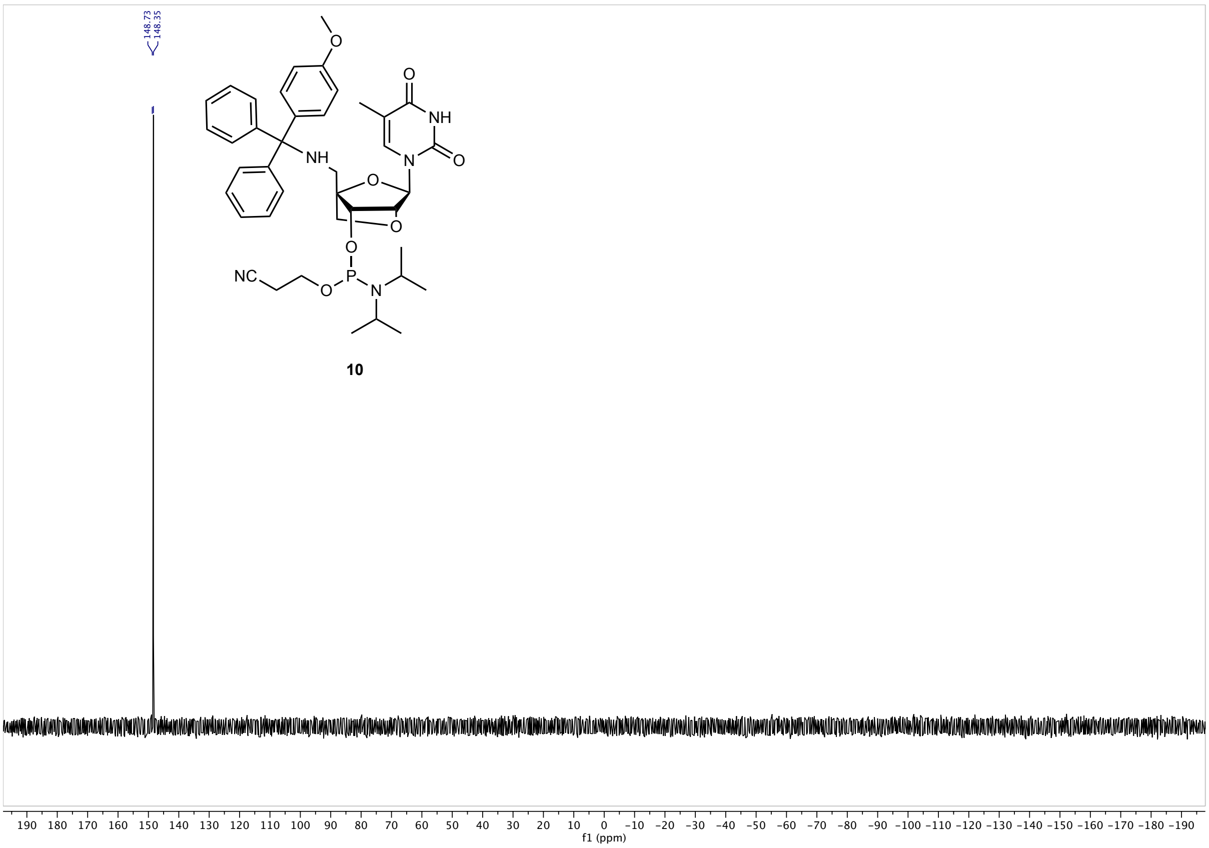

Supplementary Figure 85. ^{31}P NMR (162 MHz, $CDCl_3$) spectra of compound **10**.

Supplementary References

1. Koshkin, A.A., Fensholdt, J., Pfundheller, H.M. & Lomholt, C. A Simplified and Efficient Route to 2′-O, 4′-C-Methylene-Linked Bicyclic Ribonucleosides (Locked Nucleic Acid). *J. Org. Chem.* **66**, 8504-8512 (2001).
2. Obika, S. et al. Synthesis and conformation of 3′,4′-BNA monomers, 3′-O,4′-C-methyleneribonucleosides. *Tetrahedron* **58**, 3039-3049 (2002).
3. Obika, S., Nakagawa, O., Hiroto, A., Hari, Y. & Imanishi, T. Synthesis and properties of a novel bridged nucleic acid with a P3′ → N5′ phosphoramidate linkage, 5′-amino-2′,4′-BNA. *Chem. Commun.*, 2202-2203 (2003).
4. Thorpe, C., Epple, S., Woods, B., El-Sagheer, A.H. & Brown, T. Synthesis and biophysical properties of carbamate-locked nucleic acid (LNA) oligonucleotides with potential antisense applications. *Org. Biomol. Chem.* **17**, 5341-5348 (2019).
5. Palatinus, L. & Chapuis, G. SUPERFLIP– a computer program for the solution of crystal structures by charge flipping in arbitrary dimensions. *J. Appl. Crystallogr.* **40**, 786-790 (2007).
6. Parois, P., Cooper, R.I. & Thompson, A.L. Crystal structures of increasingly large molecules: meeting the challenges with CRYSTALS software. *Chem. Cent. J.* **9**, 30 (2015).
7. Cooper, R.I., Thompson, A.L. & Watkin, D.J. CRYSTALS enhancements: dealing with hydrogen atoms in refinement. *J. Appl. Crystallogr.* **43**, 1100-1107 (2010).
8. Kopka, M.L., Lavelle, L., Han, G.W., Ng, H.-L. & Dickerson, R.E. An Unusual Sugar Conformation in the Structure of an RNA/DNA Decamer of the Polypurine Tract May Affect Recognition by RNase H. *J. Mol. Biol.* **334**, 653-665 (2003).
9. Winter, G. & McAuley, K.E. Automated data collection for macromolecular crystallography. *Methods* **55**, 81-93 (2011).
10. Winter, G. et al. DIALS: implementation and evaluation of a new integration package. *Acta Crystallogr. D Struct. Biol.* **74**, 85-97 (2018).
11. Winter, G. xia2: An Expert System for Macromolecular Crystallography Data Reduction. *J. Appl. Crystallogr.* **43**, 186-190 (2010).
12. Lebedev, A.A. et al. JLigand: a graphical tool for the CCP4 template-restraint library. *Acta Crystallogr. D Biol. Crystallogr.* **68**, 431-440 (2012).
13. Rossmann, M.G. The molecular replacement method. *Acta Crystallogr. A* **46 (Pt 2)**, 73-82 (1990).
14. McCoy, A.J. et al. Phaser crystallographic software. *J. Appl. Crystallogr.* **40**, 658-674 (2007).
15. Murshudov, G.N., Vagin, A.A. & Dodson, E.J. Refinement of macromolecular structures by the maximum-likelihood method. *Acta Crystallogr. D Biol. Crystallogr.* **53**, 240-255 (1997).
16. Murshudov, G.N. et al. REFMAC5 for the refinement of macromolecular crystal structures. *Acta Crystallogr. D Biol. Crystallogr.* **67**, 355-367 (2011).
17. Liebschner, D. et al. Macromolecular structure determination using X-rays, neutrons and electrons: recent developments in Phenix. *Acta Crystallogr. D Struct. Biol.* **75**, 861-877 (2019).
18. Long, F. et al. AceDRG: a stereochemical description generator for ligands. *Acta Crystallogr. D Struct. Biol.* **73**, 112-122 (2017).
19. Winn, M.D. et al. Overview of the CCP4 suite and current developments. *Acta Crystallogr. D Biol. Crystallogr.* **67**, 235-242 (2011).
20. Evans, P.R. An introduction to data reduction: space-group determination, scaling and intensity statistics. *Acta Crystallogr. D Biol. Crystallogr.* **67**, 282-292 (2011).
21. Kang, S.-H., Cho, M.-J. & Kole, R. Up-Regulation of Luciferase Gene Expression with Antisense Oligonucleotides: Implications and Applications in Functional Assay Development. *Biochemistry* **37**, 6235-6239 (1998).
