# Supplementary material for: An LNA-amide modification that enhances the cell uptake and activity of phosphorothioate exon-skipping oligonucleotides
Source: Nat Commun. 2022 Jul 12;13:4036. doi: 10.1038/s41467-022-31636-2 (PMC9276774; doi:10.1038/s41467-022-31636-2)
Supplement: Supplementary file 3 — Reporting Summary [file 41467_2022_31636_MOESM3_ESM.pdf]

nature research

Corresponding author(s): Tom Brown

Last updated by author(s): May 30, 2022

Reporting Summary

Nature Research wishes to improve the reproducibility of the work that we publish. This form provides structure for consistency and transparency in reporting. For further information on Nature Research policies, see our Editorial Policies and the Editorial Policy Checklist.

Statistics

For all statistical analyses, confirm that the following items are present in the figure legend, table legend, main text, or Methods section.

n/a	Confirmed	
☐	☒	The exact sample size (*n*) for each experimental group/condition, given as a discrete number and unit of measurement
☐	☒	A statement on whether measurements were taken from distinct samples or whether the same sample was measured repeatedly
☐	☒	The statistical test(s) used AND whether they are one- or two-sided<br>*Only common tests should be described solely by name; describe more complex techniques in the Methods section.*
☒	☐	A description of all covariates tested
☒	☐	A description of any assumptions or corrections, such as tests of normality and adjustment for multiple comparisons
☐	☒	A full description of the statistical parameters including central tendency (e.g. means) or other basic estimates (e.g. regression coefficient) AND variation (e.g. standard deviation) or associated estimates of uncertainty (e.g. confidence intervals)
☐	☒	For null hypothesis testing, the test statistic (e.g. *F*, *t*, *r*) with confidence intervals, effect sizes, degrees of freedom and *P* value noted<br>*Give P values as exact values whenever suitable.*
☒	☐	For Bayesian analysis, information on the choice of priors and Markov chain Monte Carlo settings
☒	☐	For hierarchical and complex designs, identification of the appropriate level for tests and full reporting of outcomes
☒	☐	Estimates of effect sizes (e.g. Cohen's *d*, Pearson's *r*), indicating how they were calculated

Our web collection on statistics for biologists contains articles on many of the points above.

Software and code

Policy information about availability of computer code

Data collection	UV melting data was collected using Cary 4000 and analysed using Cary WinUV 3.0 software<br><br>For luciferase and viability assays, measurements were taken on a CLARIOstar microplate reader (BMG Labtech) and analysed with CLARIOstar software version 5.21.R2<br><br>Polyacrylamide gel (PAGE) was visualised by UV shadowing using G:BOX gel Imager and GeneSnap 7.12 software from SynGene<br><br>Single crystal X-ray diffraction data were collected using a (Rigaku) Oxford Diffraction SuperNova diffractometer and CrysAlisPro v41.117a<br><br>Oligonucleotide mass spectra and UPLC data were collected and analysed on a UPLC-MS Waters XEVO G2-QTOF mass spectrometer and an Acquity UPLC system with MassLynx v4.1 software<br><br>NMR data was collected using Bruker IconNMR 5.0.10 Build 19 for TopSpin 3.6.3 or Bruker IconNMR 5.0.11 Build 10 for TopSpin 3.6.3
Data analysis	DNA X-ray structural data were auto processed using either fast_dp (1.6.2), xia2_dials (xia2 0.6.475, DIALS 2.2.10) or xia2_3dii (xia2 0.6.475, XDS 20210205). (references given in Supporting Information) Model refinement was performed using REFMAC5 (5.0.32) and PHENIX.REFINE within the Phenix software suite (1.19.2). Geometric restraints for the non-standard phosphoribosyl backbones were generated using JLIGAND (1.0.40) or ACEDRG (222). Software packages and project management was handled using CCP4 (7.0.065) and Phenix (1.19.2). Images were made using PYMOL graphic software (The PyMOL Molecular Graphics System, Version 2.3.2 Schrödinger, LLC).<br><br>Small molecule crystal structures were solved using 'Superflip' Crystals 03/15/13 before refinement with CRYSTALS (v10.1.100 commit 8199)

NMR and small molecule mass data was analysed using MestReNova (v14.0.1-23559)

Cell assay data was analysed using Prism Graphpad (Prism 9 for macOS Version 9.2.0 (283))

UV melting curves were replotted in Microsoft Excel for Mac Version 16.16.27 (201012)

For manuscripts utilizing custom algorithms or software that are central to the research but not yet described in published literature, software must be made available to editors and reviewers. We strongly encourage code deposition in a community repository (e.g. GitHub). See the Nature Research guidelines for submitting code & software for further information.

Data

Policy information about availability of data

All manuscripts must include a data availability statement. This statement should provide the following information, where applicable:

- Accession codes, unique identifiers, or web links for publicly available datasets
- A list of figures that have associated raw data
- A description of any restrictions on data availability

Crystallographic data for the structures in this Article have been deposited with the Cambridge Crystallographic Data Centre (CCDC 2105684-5) and can be obtained via www. ccdc.cam.ac.uk/data_request/cif.

DNA structural data obtained by X-ray crystallography were deposited in the Protein Data Bank (PDB) and are available with the following accession codes 7NRP [http://doi.org/10.2210/pdb7NRP/pdb], 7OOS [http://doi.org/10.2210/pdb7OOS/pdb], 7OZZ [http://doi.org/10.2210/pdb7OZZ/pdb] and 7OOO [http://doi.org/10.2210/pdb7OOO/pdb].

The authors declare that all other data supporting the findings of this study are available within the paper and its supplementary information files.

Field-specific reporting

Please select the one below that is the best fit for your research. If you are not sure, read the appropriate sections before making your selection.

[x] Life sciences [ ] Behavioural & social sciences [ ] Ecological, evolutionary & environmental sciences

For a reference copy of the document with all sections, see nature.com/documents/nr-reporting-summary-flat.pdf

Life sciences study design

All studies must disclose on these points even when the disclosure is negative.

Sample size	Cell-based assays (luciferase assays and viability assays performed on HeLa pLuc/705 cells, Figure 6a-d) were performed in three biological replicates (n=3) and also repeated in technical duplicate. Sample size was determined based on similar experiments in the literature and typical variability for each experiment based on our previous experience with these techniques (Nucleic Acids Research, 2011, Vol. 39, No. 12; Molecular Therapy - Nucleic Acids, Volume 3, e212; Biochemistry, Vol. 37, No. 18, 1998 6237; Nucleic Acid Therapeutics. Dec 2016.381-391.http://doi.org/10.1089/nat.2016.0631).
Data exclusions	No data was excluded
Replication	Replication was successful. Positive and negative control oligonucleotides were used allowing us to confirm that the assay was working. The positive control had the same sequence and modifications as the oligonucleotide previously reported. All experiments were performed at least twice and all findings were reliably reproduced.
Randomization	Cells were counted and the same number of cells were seeded for each well on a 96-well plate for all the cell-based assays. Control and treated cells were analyzed equally and for luciferase assays. Given the monoclonality of the HELA pLuc/705 cells used in the study and the lack of experimental variability, there was no requirement for randomization.
Blinding	For experiments involved CLARIOstar microplate reader (Figure 6a-d, S24-25), blinding was not relevant because 96-well plate scanning and signal measurement were fully automated, with no manual intervention. Micrographs of cells (Figure 6e and S26) shown are the representations from > = 10 images taken per condition, by a blinded investigator.

Reporting for specific materials, systems and methods

We require information from authors about some types of materials, experimental systems and methods used in many studies. Here, indicate whether each material, system or method listed is relevant to your study. If you are not sure if a list item applies to your research, read the appropriate section before selecting a response.

Materials & experimental systems

n/a	Involved in the study
☒	☐ Antibodies
☐	☒ Eukaryotic cell lines
☒	☐ Palaeontology and archaeology
☒	☐ Animals and other organisms
☒	☐ Human research participants
☒	☐ Clinical data
☒	☐ Dual use research of concern

Methods

n/a	Involved in the study
☒	☐ ChIP-seq
☒	☐ Flow cytometry
☒	☐ MRI-based neuroimaging

Eukaryotic cell lines

Policy information about cell lines

Cell line source(s)	HeLa pLuc/705 cells were a gift from Professor Samir El Andaloussi. HeLa pLuc/705 cells were originally developed by Kole and colleagues (Biochemistry 1998, 37, 18, 6235–6239).
Authentication	None used. The positive and negative control oligonucleotides worked as expected in the reporter cell line
Mycoplasma contamination	Cell line tested negative for mycoplama
Commonly misidentified lines (See ICLAC register)	No commonly misidentified cell lines were used.
